# Enantioselective Synthesis of α-Chiral Bicyclo[1.1.1]pentanes via Multicomponent Asymmetric Allylic Alkylation

**DOI:** 10.1021/acs.orglett.4c00902

**Published:** 2024-04-30

**Authors:** Sergio Barbeira-Arán, Irene Sánchez-Sordo, Martín Fañanás-Mastral

**Affiliations:** Centro Singular de Investigación en Química Biolóxica e Materiais Moleculares (CiQUS), Universidade de Santiago de Compostela, 15782 Santiago de Compostela, Spain

## Abstract

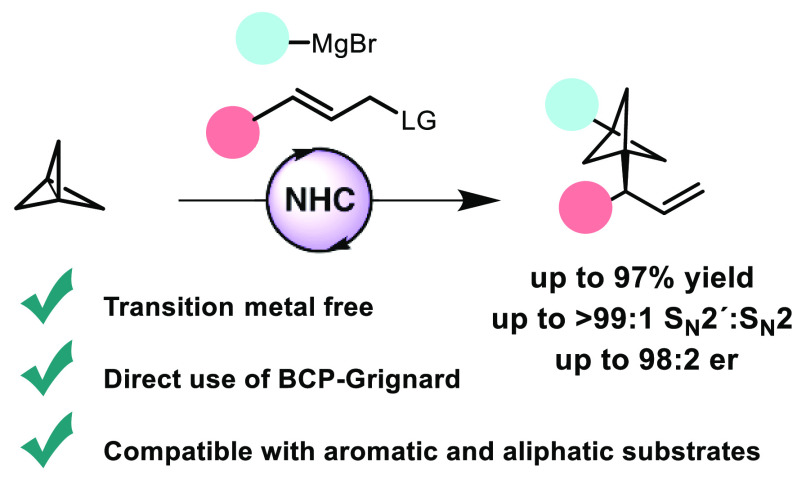

Bicyclo[1.1.1]pentanes (BCPs) have emerged as important structural motifs in drug design. However, asymmetric transformations that provide chiral BCPs bearing an adjacent stereocenter are still scarce. Here, we report a catalytic methodology for the enantioselective synthesis of α-chiral 1,3-difunctionalized BCPs from a three-component coupling of [1.1.1]propellane, a Grignard reagent, and an allylic phosphate. The reaction proceeds via the addition of the Grignard reagent to [1.1.1]propellane followed by an asymmetric *N*-heterocyclic carbene (NHC)-catalyzed allylic substitution of the resulting BCP–Grignard, providing a broad range of α-chiral BCPs with excellent levels of regioselectivity and enantioselectivity.

The creation of new drugs by structural modification of known bioactive compounds to improve their physicochemical properties represents a very important tool in the pharmaceutical industry.^1^ In this context, some studies point to the fact that drug development is more likely to be successful for compounds having a small number of aromatic rings and a more three-dimensional structure.^2^ In this sense, highly strained carbocycles, such as bicyclo[1.1.1]pentanes (BCPs), have attracted much interest because they can act as bioisosteres of *para*-substituted phenyl rings, *tert*-butyl groups, or internal alkynes, leading to new drugs with improved aqueous solubility, membrane permeability, and/or metabolic stability (Scheme 1a).^3^ Because benzylic, propargylic, or neopentylic chiral centers are common motifs in bioactive molecules, catalytic asymmetric methodologies for the synthesis of α-chiral BCPs are highly warranted. However, despite the great advances on the development of synthetic methodologies to access BCP derivatives,^4^ there is still a limited number of methods for the enantioselective synthesis of chiral BCPs bearing an α stereocenter.^5^ Those methodologies have typically relied on multistep processes where [1.1.1]propellane is first transformed into functionalized BCP, in which the stereogenic center is subsequently installed using a chiral reagent,^6^ chiral auxiliary,^7^ or asymmetric transition metal catalysis.^8^ In comparison to these multistep protocols, the direct asymmetric functionalization of [1.1.1]propellane represents a more straightforward manner to access chiral BCPs, although this area remains less explored. In this regard, few protocols based on the diastereoselective trapping of a catalytically generated BCP radical with chiral sulfinimine have been reported.^9^ To the best of our knowledge, only two examples involving the direct enantioselective functionalization of [1.1.1]propellane have been described thus far. Those methodologies include the synthesis of chiral monosubstituted BCP alcohols through the merge of asymmetric organocatalysis and photoredox catalysis, reported by Anderson and co-workers,^10^ and the synthesis of 1,3-difunctionalized BCPs by a multicomponent iridium-catalyzed asymmetric allylic substitution, described by Aggarwal and co-workers^11^ (Scheme 1b). The latter strategy involves the formation of a BCP–Grignard reagent by the addition of an aryl or alkyl Grignard reagent to [1.1.1]propellane,^3e^ followed by transmetalation with ZnCl_2_ to produce a BCP–zinc compound that undergoes an enantioselective allylic substitution of allylic carbonate catalyzed by a chiral Ir/phosphoramidite complex. Of note, the authors reported that transmetalation to ZnCl_2_ was necessary because the direct use of BCP–Grignard led to significantly diminished regio- and enantioselectivity.

**Scheme 1:**
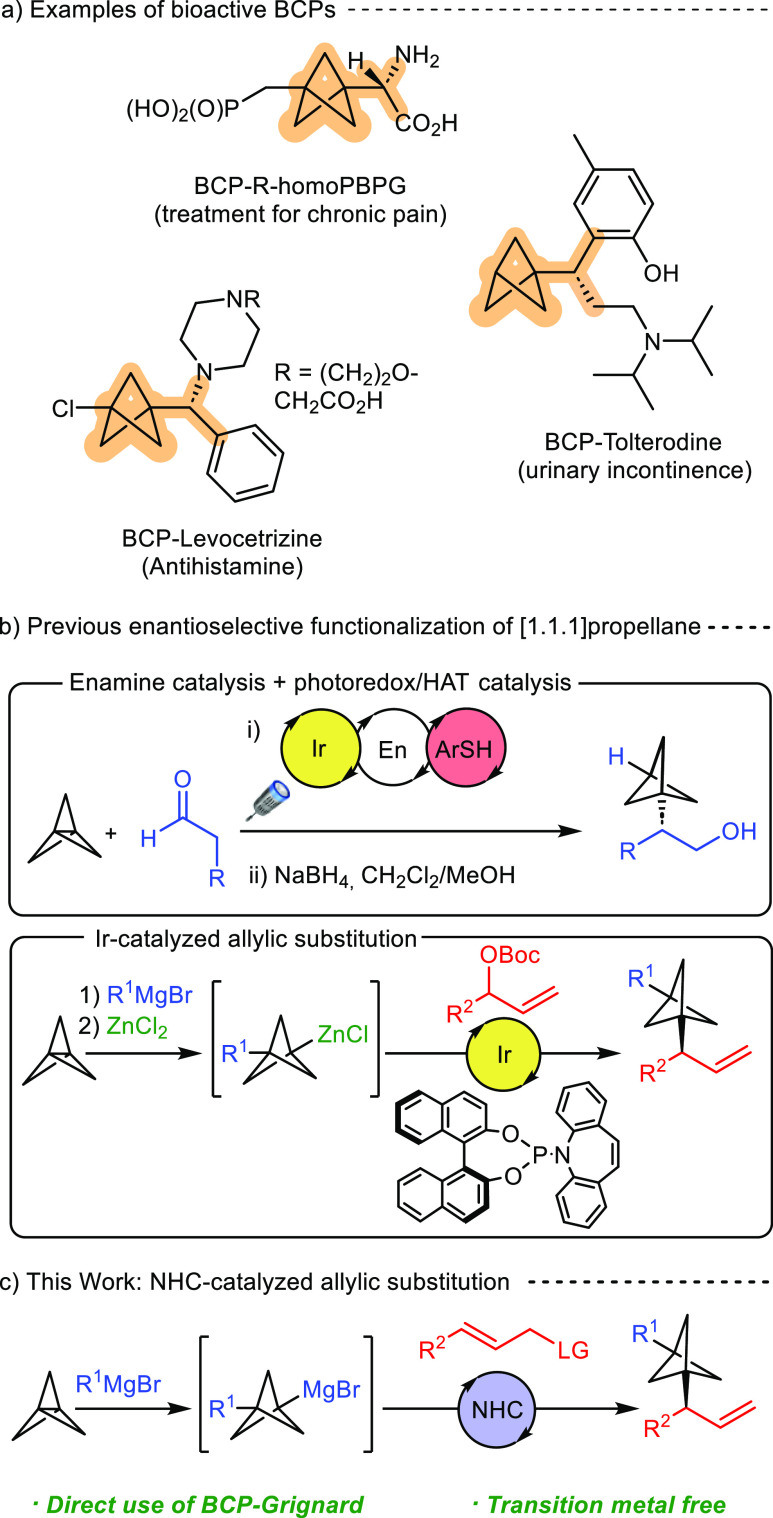
Bioactive α-Chiral BCPs and Current Methodologies for the Enantioselective Functionalization of [1.1.1]Propellane

Given the ability of chiral copper complexes^12^ and *N*-heterocyclic carbenes (NHCs)^13,14^ to efficiently catalyze the enantioselective allylic alkylation of Grignard reagents, we envisaged this type of catalysis as a potential platform for a more straightforward access to α-chiral difunctionalized BCPs that would avoid the stoichiometric use of ZnCl_2_ and the use of a precious transition metal catalyst (Scheme 1c). Importantly, while both copper and NHC catalysis have been employed in highly regio- and enantioselective allylic alkylation of primary and secondary Grignard reagents,^12^ the direct use of tertiary BCP–Grignard imposes an important selectivity challenge in the regio- and enantioselective formation of the desired branched product, as illustrated with the low selectivities observed in the few cases where the use of a tertiary Grignard reagent, such as *t*-BuMgBr, has been reported.^14a,14f^ We report here the development of a three-component catalytic transition-metal-free enantioselective direct difunctionalization of [1.1.1]propellane that proceeds through a chiral NHC-catalyzed allylic alkylation of an *in situ* prepared tertiary BCP–Grignard reagent and provides α-chiral BCPs with high levels of regio- and enantioselectivity.

We began our study by investigating the copper-catalyzed alkylation of diethyl cinnamyl phosphate **3** with BCP–Grignard, resulting from the coupling of PhMgBr and [1.1.1]propellane. Different screening studies (see the Supporting Information) revealed the system comprising CuCN and sulfonate-bearing NHC ligand **L1** as an efficient catalyst for this transformation (entry 1 in Table 1). Remarkably, in a control experiment run in the absence of the copper salt, we observed that chiral NHC **L1** is an even more efficient catalyst for this transformation, providing the product with improved regio- and enantioselectivity (>99:1 rr and 97:3 er; entry 2). This result came as a surprise because sulfonate-bearing NHCs were reported by Hoveyda and co-workers to not be efficient nor selective catalysts for the copper-free allylic alkylation of allylic phosphates with alkyl Grignard reagents, in contrast to dialkylzinc or organoaluminum compounds.^13b^ We believe that this different behavior hints at the diminished reactivity of BCP–Grignard when compared to simpler alkyl Grignard reagents. Indeed, the reaction in the absence of NHC **L1** led to a very low yield, with the linear product being the major isomer (entry 3). This result suggests that Lewis base activation is required for an efficient alkylation in the case of BCP–Grignard. Further NHC screening revealed that changes in the substitution pattern of the *N*-aryl unit caused a significant decrease in the efficiency and/or the selectivity of the reaction (entries 4–6). The presence of the sulfonate group also proved essential for the reaction because the use of other bidentate NHCs bearing other Lewis basic sites, such as ligand **L5**, led to a diminished yield and negligible enantioselectivity (entry 7). The solvent played an important role, with tetrahydrofuran (THF) being the solvent of choice. Less coordinating solvents, such as Et_2_O, led to significantly diminished regio- and enantioselectivity (entry 8). Similarly, the leaving group of the allylic substrate was important for the reaction, as illustrated with the lower regio- and enantioselectivity obtained when cinnamyl bromide was used (entry 9). Evaluation of different temperatures (see the Supporting Information) revealed 50 °C to be optimal, obtaining worse results at a lower temperature likely as a result of a lower efficiency of the NHC–BCP–Grignard complex under those conditions (entry 10). The reaction could be carried out with 1.5 equiv of PhMgBr and [1.1.1]propellane, although compound **4** was obtained in a lower yield (entry 11). Finally, the catalyst loading could be lowered to 6 mol % without affecting the efficiency or selectivity of the reaction (entry 12).

**Table 1:**
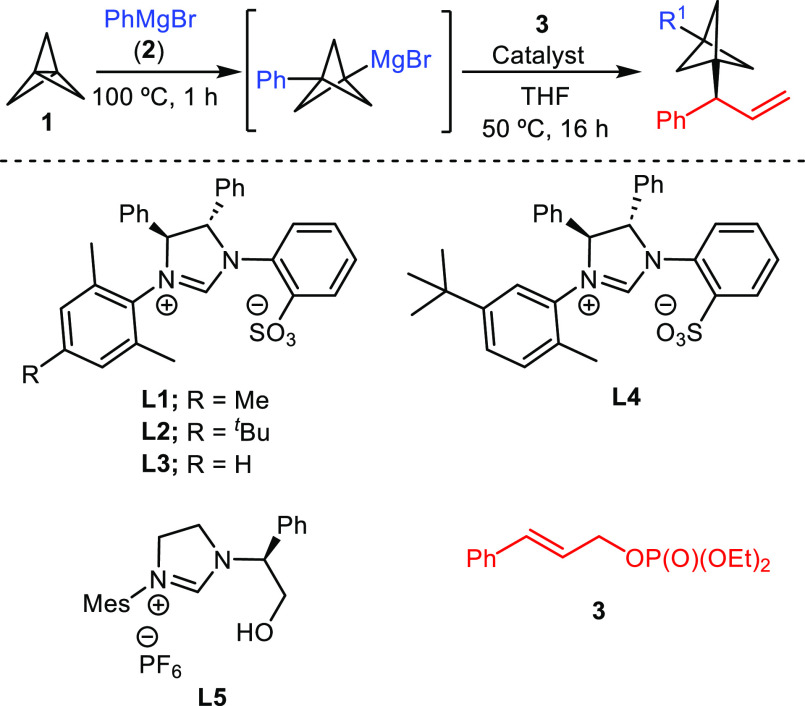
Optimization Studies^a^

entry	catalyst	yield^b^	rr^c^	er^d^
1	CuCN/**L1**^e^	70	95:5	92:8
2	**L1**	65	>99:1	97:3
3	-	10	10:90	-
4	**L2**	0	-	-
5	**L3**	44	50:50	73:27
6	**L4**	56	92:8	89:11
7	**L5**	38	83:17	50:50
8^f^	**L1**	55	90:10	78:22
9^g^	**L1**	67	92:8	72:28
10^h^	**L1**	59	85:15	91:9
11^i^	**L1**	45	>99:1	97:3
12	**L1**^j^	65	>99:1	97:3

a Reaction conditions: compound **3** (0.2 mmol), compound **1** (0.5 mmol), compound **2** (0.5 mmol), catalyst (10 mol %), THF (2 mL), and 50 °C.

b Yield of the isolated product.

c Regioisomeric ratio (rr, branched/linear) was determined by gas chromatography–mass spectrometry (GC–MS) analysis.

d Enantiomeric ratio (er) was determined by supercritical fluid chromatography (SFC) analysis.

e NaO^*t*^Bu (20 mol %) was used as a base.

f Et_2_O instead of THF.

g Br as a leaving group.

h The reaction was run at 30 °C.

i Compound **1** (1.5 equiv, 0.3 mmol) and compound **2** (1.5 equiv, 0.3 mmol).

j At 6 mol %.

Once having established the optimized conditions for this transition-metal-free enantioselective difunctionalization of [1.1.1]propellane, we set out to explore the scope of the reaction (Scheme 2). In all cases, the corresponding branched product was obtained as a sole isomer with excellent regiocontrol (>98:2 rr). Cinnamyl phosphate derivatives featuring different substitution patterns proved to be efficient for this transformation, furnishing the corresponding chiral allyl BCPs **4**–**9** in a generally high yield with good to excellent enantioselectivity (up to 98:2 er). Notably, the use of (*Z*)-allylic phosphate (*Z*-**3**) afforded chiral BCP **4** in a good yield and excellent regioselectivity, albeit with a significantly diminished enantiomeric ratio (41:59 er) in favor of the opposite enantiomer, thus showing that the enantioselectivity of the reaction is influenced by the *E*/*Z* configuration of the starting allyl substrate. Importantly, allylic phosphates bearing aliphatic substituents were also efficient partners in this transformation. The reaction was remarkably effective with substrates bearing secondary alkyl groups, such as isopropyl (**10**) or cyclohexyl (**11**), or a tetrahydropyranyl group (**12**) and furnished the corresponding chiral difunctionalized BCPs in a high yield with excellent enantioselectivity (97:3–98:2 er). Substrates bearing a methyl group (**13**) or primary alkyl substituents (**14** and **15**) were also efficient, although a slight decrease in enantioselectivity was observed in those cases. Considering that allylic substrates bearing aliphatic substituents are typically reluctant coupling partners in iridium-catalyzed enantioselective allylic substitution,^15^ as also reported by Aggarwal and co-workers in the iridium-catalyzed allylic substitution of BCP–zinc reagents,^11^ the examples presented herein involving the use of these type of allylic substrates are particularly relevant because they open a new enantioselective route to new types of fully aliphatic chiral 1,3-disubstituted BCPs.

**Scheme 2:**
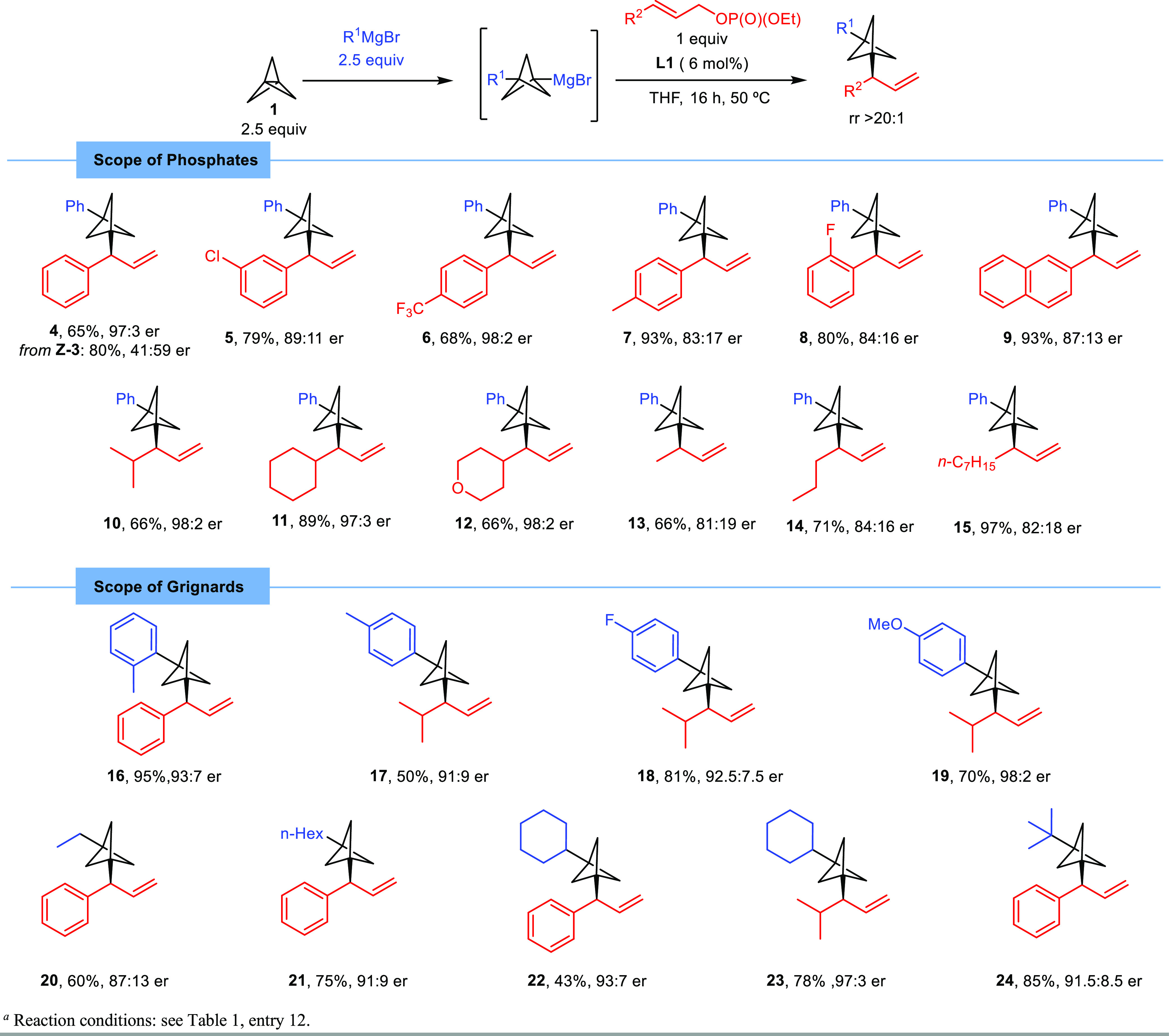
Scope of the Reaction Reaction conditions: see entry 12 in Table 1.

We next explored the scope of the reaction with respect to the Grignard reagent. Aryl Grignard reagents bearing both electron-donating and -withdrawing groups could be efficiently used and provided the corresponding chiral BCP products **16**–**19** in good yields with high enantioselectivity. Alkyl Grignard reagents were also compatible for this transformation, allowing for the enantioselective difunctionalization of [1.1.1]propellane with primary (**20** and **21**), secondary (**22** and **23**), and even tertiary (**24**) alkyl groups.

In some cases, product derivatization was necessary to achieve chiral separation for enantiomeric ratio determination (see the Supporting Information for details). This was done via cross-metathesis or hydroboration/oxidation protocols, which also illustrate the synthetic versatility of the obtained chiral BCPs (Scheme 3).

**Scheme 3:**
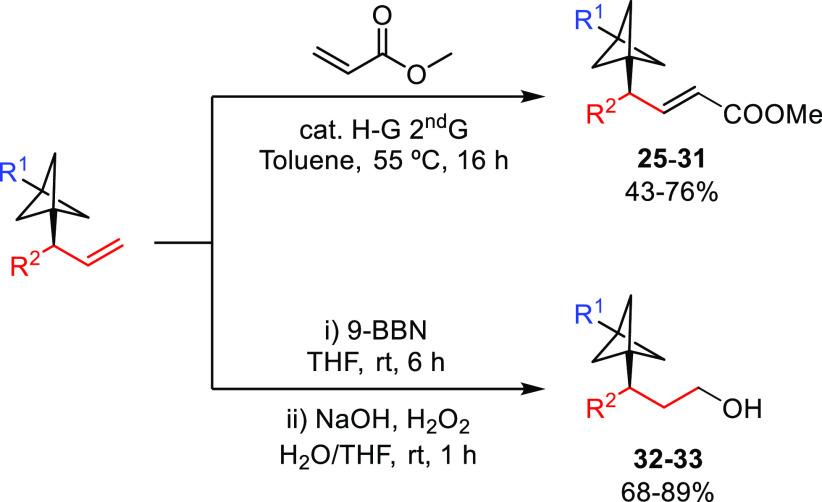
Synthetic Modifications of Products

Absolute configuration of the products was assigned by comparison of the optical rotation to the reported value for the previously synthesized product **16**.^11^ The observed stereoinduction can be rationalized according to the stereochemical model depicted in Scheme 4. On the basis of previous studies on the structure of NHC–Mg complexes generated from the reaction of sulfonate-bearing imidazolinium salts and alkylmagnesium halides,^13b^ we propose the formation of chiral bidentate magnesium complex **I** upon reaction between ligand **L1** and BCP–Grignard. Intermediate **I** may serve as a bifunctional catalyst, in which the Lewis acidity of the Mg atom is enhanced, favoring coordination to the oxygen atom of the phosphate group of the allylic substrate, while Lewis basic oxygen of the sulfonate group coordinates to the Mg center of BCP–Grignard, thus enhancing its nucleophilicity. These structural features promote a catalyst–substrate interaction that leads to intermediate **II**, where BCP–Grignard is oriented in a position that favors the S_N_2′ mode of addition through the Si face of the allylic substrate, in agreement with the observed regio- and enantioselectivity.

**Scheme 4:**
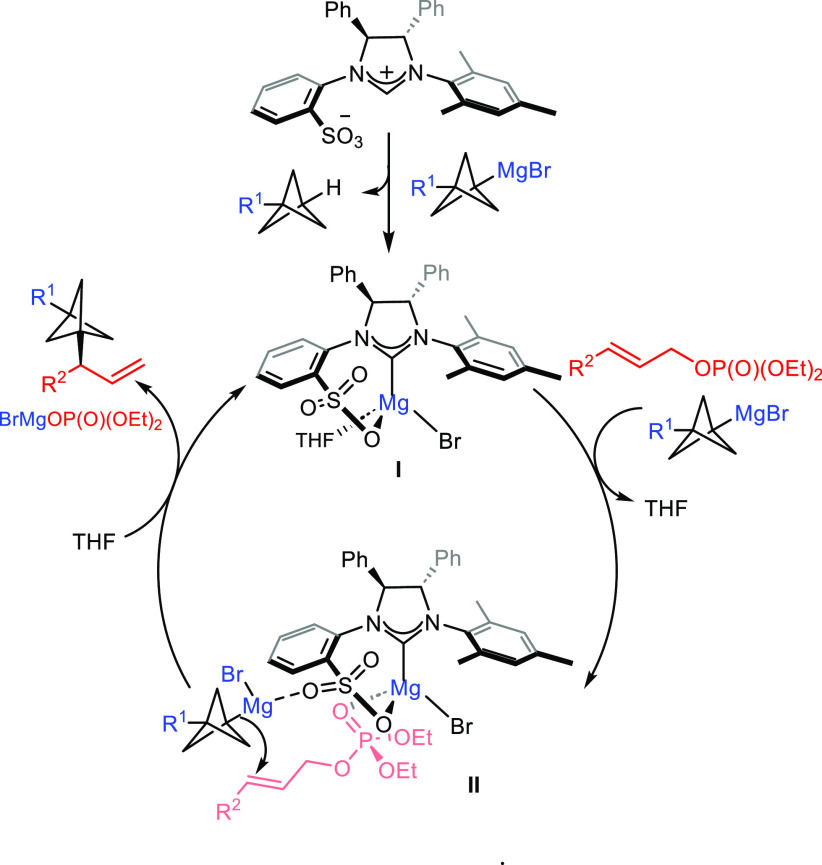
Proposed Mechanism and Stereochemical Model

In summary, we have developed a catalytic methodology for the enantioselective multicomponent coupling between [1.1.1]propellane, a Grignard reagent, and allylic phosphate. The method is based on an asymmetric NHC-catalyzed allylic substitution of an *in situ* formed BCP–Grignard by reaction of [1.1.1]propellane with a Grignard reagent and provides α-chiral 1,3-difunctionalized BCPs with excellent levels of regio- and enantioselectivity. Remarkable features of this method are the absence of transition metals and the key association of Lewis basic NHC with BCP–Grignard, which enhances its reactivity toward the allylic substrate.

## Data Availability

The data underlying this study are available in the published article and its online Supporting Information.

## Supporting Information Available

Optimization tables, synthetic procedures, and characterization data (PDF)
