# Supplementary material for: Enantioselective Synthesis of α-Chiral Bicyclo[1.1.1]pentanes via Multicomponent Asymmetric Allylic Alkylation
Source: Org Lett. 2024 Apr 30;26(18):3784–9. doi: 10.1021/acs.orglett.4c00902 (PMC11091886; doi:10.1021/acs.orglett.4c00902)
Supplement: Supplementary file 1 — ol4c00902_si_001.pdf [file ol4c00902_si_001.pdf]

Supporting information

Enantioselective synthesis of α-chiral BCPs via multicomponent asymmetric allylic alkylation

Sergio Barbeira-Arán, Irene Sánchez-Sordo and Martín Fañanás-Mastral*

Centro Singular de Investigación en Química Biolóxica e Materiais Moleculares (CiQUS), Universidade de Santiago de Compostela, 15782 Santiago de Compostela, Spain.

Correspondence to: martin.fananas@usc.es

Table of contents

1. General methods 3

2. Safety considerations 4

3. List of starting materials and chiral ligands 5

4. Synthesis of [1.1.1]propellane 6

5. Optimization of the regio- and enantioselective synthesis of chiral bicyclo[1.1.1]pentane derivatives via copper-catalyzed asymmetric allylic substitution. 7

5.1 Proposed Reaction 7

5.2 Screening of chiral Ligands 7

5.3 Screening of solvents 8

5.4 Screening of copper salts 8

5.5 Screening of reagents stoichiometry 9

5.6 Catalyst Loading 9

6. Optimization of the regio- and enantioselective synthesis of chiral bicyclo[1.1.1]pentane derivatives via transition metal free asymmetric allylic substitution. 10

6.1 Proposed Reaction 10

6.2 Screening of chiral Ligands 10

6.3 Screening of solvents 10

6.4 Screening of Leaving group 11

6.5 Screening of reagents stoichiometry 11

6.6 Screening of temperature 11

6.7 NHC Loading 12

7. General procedure for the synthesis of allylic phosphates (General Procedure A) 12

8. General Procedure for the regio- and enantioselective synthesis of α-chiral bicyclo[1.1.1]pentanes (General Procedure B) 13

9. Product characterization 13

10. Derivatization of the chiral bicyclo[1.1.1]pentanes by olefin metathesis (General procedure C) 24

11. Derivatization of chiral bicyclo[1.1.1]pentanes by hydroboration/oxidation (General procedure D) 29

12. Unsuccessful examples 30

13. NMR spectra 31

14. References 67

1. General methods

• All reactions were performed under argon atmosphere using oven dried glassware and using standard Schlenk techniques. Solvents were dried using an MBraun SPS 800 system. All chemicals were purchased from Acros Organics Ltd., Aldrich Chemical Co. Ltd., Alfa Aesar, Apollo, BLDpharm, Strem Chemicals Inc., Fluorochem Ltd. or TCI Europe N.V. chemical companies and used without further purification, except from 1,1-dibromo-2,2-bis (chloromethyl)cyclopropane which was purified by flash column chromatography in hexane prior to use.

• Analytical thin layer chromatography was carried out on silica-coated aluminium plates (silica gel 60 F254 Merck) and components were visualized by UV light and $KMnO_4$ staining. Flash column chromatography was performed on silica gel 60 (Merck, 230-400 mesh) without previous deactivation, unless otherwise stated.

• High Resolution Mass spectrometry was carried out on a Bruker microTOF spectrometer using ESI or APCI.

• ^{1}H, ^{13}C, ^{31}P and ^{19}F NMR experiments were carried out using a Bruker AVIII 500MHz or a Varian Mercury 300MHz NMR spectrometer. Chemical shift values are reported in ppm with the solvent resonance as the internal standard ($CHCl_3$: δ 7.26 for ^{1}H, δ 77.16 for ^{13}C). Coupling constants (*J*) are given in Hertz (Hz). Multiplicities are reported as follows: s = singlet, d = doublet, t = triplet, q = quartet, m = multiplet or as a combination of them.

• Melting points were determined using a Buchi-M565 apparatus.

• Optical rotation was determined in a Jasco P-2000 Polarimeter.

• Enantiomeric ratios were determined by Supercritical Fluid Chromatography (SFC) analysis in a Jasco Series 4000 instrument or by High Performance Liquid Chromatography (HPLC) analysis using a WATERS ACQUITY Arc System, consisting of a quaternary pump, column oven and autosampler coupled with a 2998 PDA detector.

2. Safety considerations

All the Grignard reagent additions to [1.1.1]propellane were performed with no more than 1.9 mL of a diethyl ether solution in a 15 mL pressure [tube height = 13 cm (15 cm including stopcock); wall thickness = 2.1 mm; outer diameter = 2.2 cm; see picture below]. The glassware used was purchased from Afora. During our studies, no incidents occurred as a result of build-up of pressure in these reactions. Nonetheless, due to the low boiling points of diethyl ether and [1.1.1]propellane and risks associated with heating this mixture to 100 °C in a sealed vessel, all reactions were performed behind a blast shield.[1]

Under the reaction conditions, the upper limit of pressure within the system can be determined from the saturated vapor pressure of diethyl ether, which is 6.5 atm at 100 °C. Consequently, it is recommended to employ pressure tubes with a pressure rating of 10 atm (150 psi). To minimize the build-up of pressure while heating the reactions, care was taken when submerging the reaction vessel in the oil bath, ensuring that the oil level only slightly exceeded that of the internal solvent level (see picture below). This promotes effective air cooling of the headspace within the reaction vessel, thereby reducing internal pressure.[1]

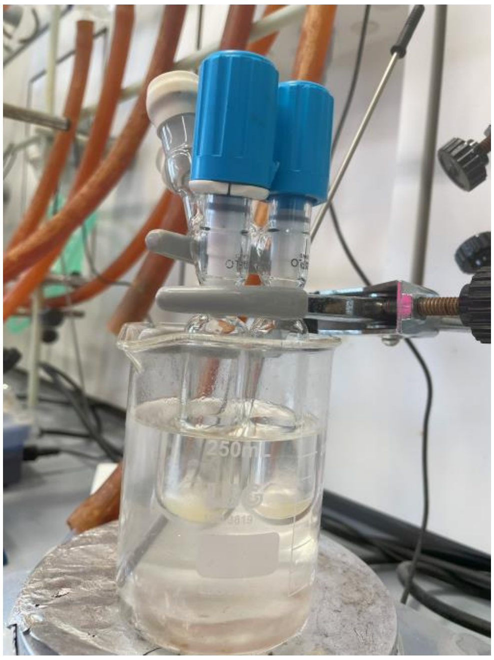

3. List of starting materials and chiral ligands

Grignard Reagents **2, 34-36, 38-44,** 3-Bromo-1-phenyl-1-propene **23, L6-11** were purchased from commercial sources. [1.1.1]Propellane **1**[2], Grignard Reagent **37**[3] allylic phosphates **3**, **45-55**[4], **56**[5], **57**,[6] **Z-3**[6] and ligands **L1**[7], **L2**[8], **L3**[8], **L4**[9], **L5**[10] were prepared according to literature procedures.

Grignard Reagents:

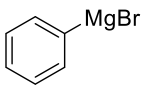

2

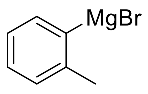

34

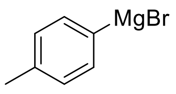

35

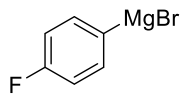

36

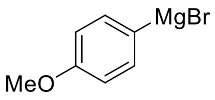

37

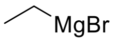

38

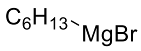

39

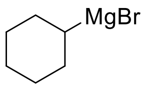

40

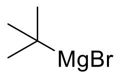

41

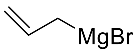

42

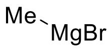

43

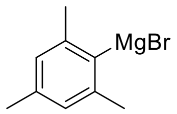

44

Allylic Phosphates:

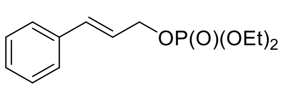

3

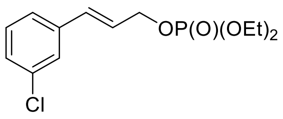

45

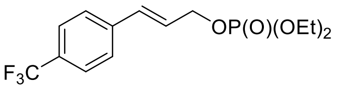

46

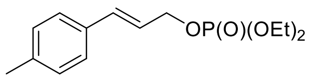

47

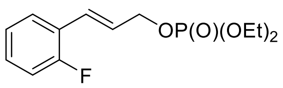

48

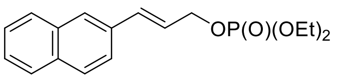

49

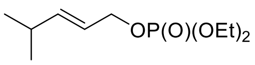

50

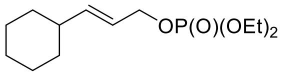

51

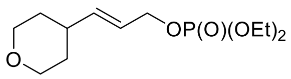

52

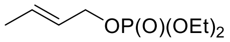

53

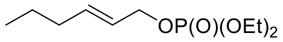

54

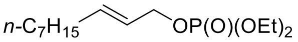

55

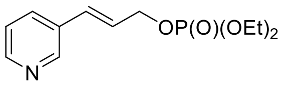

56

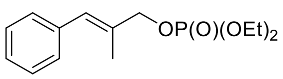

57

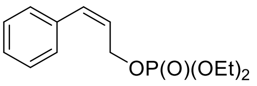

Z-3

Ligands:

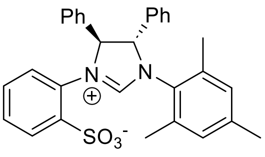

L1

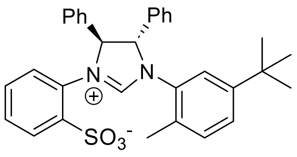

L2

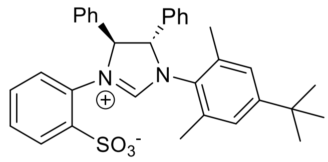

L3

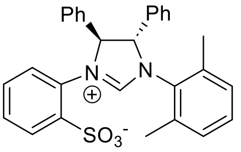

L4

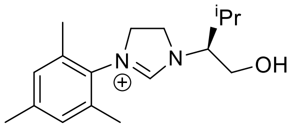

L5

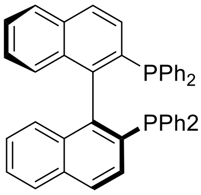

L6

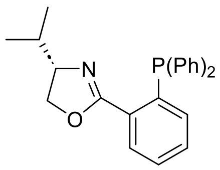

L7

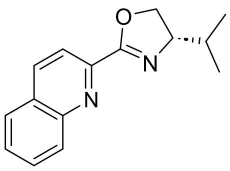

L8

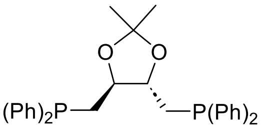

L9

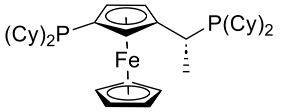

L10

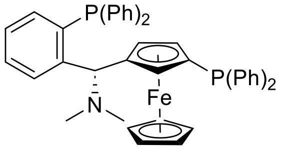

L11

4. Synthesis of [1.1.1]propellane

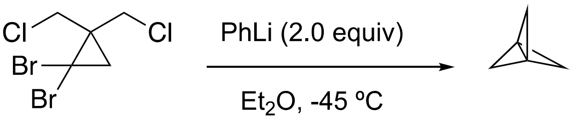

In a flame-dried Schlenk flask, 1,1-dibromo-2,2-bis (chloromethyl)cyclopropane (38 mmol, 1.0 equiv) was dissolved in Et_2O (25 mL) at -45 °C. Phenyllithium (1.9 M in dibutylether, 76 mmol) was added dropwise over 40 min. After the addition was complete, the mixture was stirred for 10 min at-45 °C and then warmed to 0 °C. After 2 h at this temperature, the [1.1.1]propellane solution was distilled under reduced pressure (~130 mbar for 40 min) at rt. A total volume of approximately 24mL of distillate was collected. An aliquot (0.05 mL) was analyzed by 'H NMR with triphenylmethane as an internal standard. The average concentration [1.1.1]propellane was 0.7-0.9 M. The solution was stored at -25 °C.

5. Optimization of the regio- and enantioselective synthesis of chiral bicyclo[1.1.1]pentane derivatives via copper-catalyzed asymmetric allylic substitution.

Note: For the optimization of regio- and enantioselective synthesis of chiral bicyclo[1.1.1]pentane derivatives via copper-catalyzed asymmetric allylic substitution, there was a variation of general procedure B. In that case, the chiral copper catalyst was pre-formed by mixing the copper salt, the base and the imidazolium salt in a vial with THF (1 ml) under inert atmosphere and agitation for 20 minutes. Subsequently, it was added to the sealed tube.

5.1 Proposed Reaction

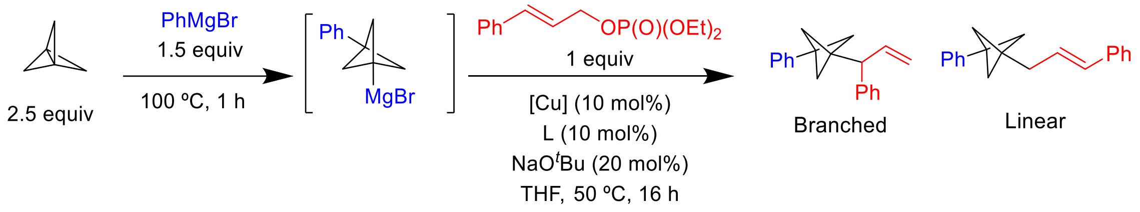

5.2 Screening of chiral Ligands

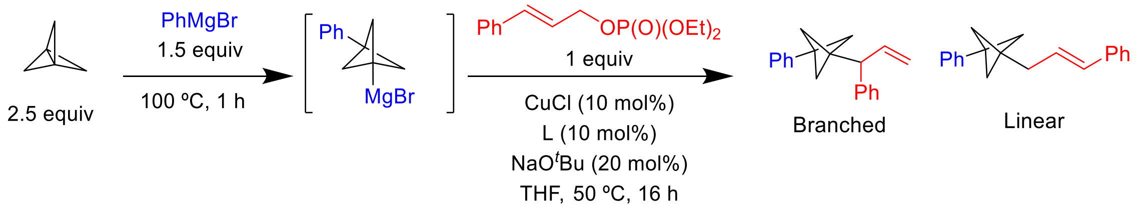

Entry [a]	Ligand	Conv [b]	Yield [b]	r.r [c]	e.r [d]
1	**L1***	**>99%**	**38**	**88:12**	**87:13**
2	**L2***	**>99%**	**60**	**68:32**	**58:42**
3	**L3***	**>99%**	**46**	**68:32**	**65:35**
4	**L4***	**>99%**	**70**	**92:08**	**86:14**
5	**L5***	**>99%**	**24**	**55:35**	**54:46**
6[e]	**L6***	**>99%**	**12**	**77:23**	**58:42**
7[e]	**L7***	**>99%**	**11**	**85:15**	**54:46**
8[e]	**L8***	**>99%**	**13**	**55:45**	**70:30**
9 [e]	**L9***	**>99%**	**11**	**58:42**	**63:37**
10[e]	**L10***	**>99%**	**27**	**59:41**	**56:44**
11[e]	**L***	**>99%**	**24**	**62:38**	**59:41**

[a] Conditions: Propellane (0.5 mmol), Grignard reagent (0.3 mmol), Allylic Phosphate (0.2 mmol), [Cu] (10 mol%) and solvent (2 ml). [b] Isolated yield. [c] rr: (Branched:Linear), ratio was determined by GC-MS. [d] er was determined by SFC [e] NaO^{t}Bu was not used

5.3 Screening of solvents

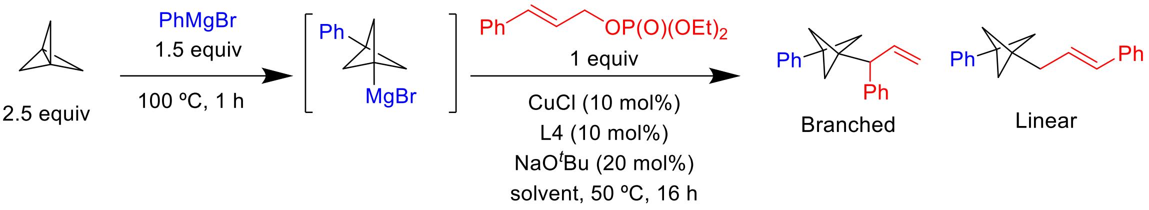

Entry [a]	Solvent	Conv [b]	Yield [b]	r.r [c]	e.r [d]
**1**	**DCM**	**>99%**	**-**	**-**	**-**
**2**	**Et_2O**	**>99%**	**42**	**89:11**	**84:16**
**3**	**Me-THF**	**>99%**	**32**	**92:08**	**82:18**
**4**	**Dioxane**	**>99%**	**14**	**91:09**	**78:22**
**5**	**Toluene**	**>99%**	**52**	**91:09**	**80:20**
**6**	**THF**	**>99%**	**70**	**92:08**	**86:14**

[a] Conditions: Propellane (0.5 mmol), Grignard reagent (0.3 mmol), Allylic Phosphate (0.2 mmol), [Cu] (10 mol%) and solvent (2 ml). [b]Isolated Yield. [c] rr: (Branched:Linear), ratio was determined by GC-MS. [d] er was determined by SFC

5.4 Screening of copper salts

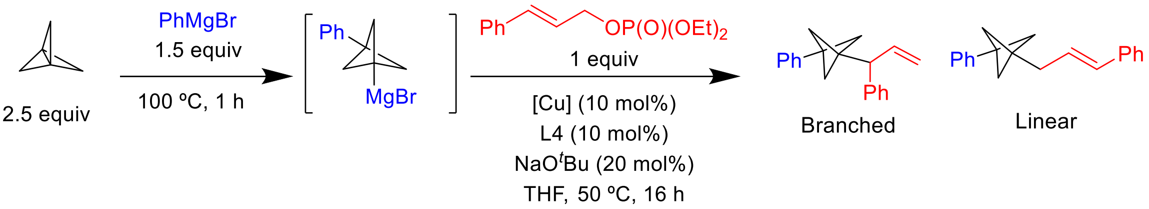

Entry [a]	[Cu]	Conv [b]	Yield [b]	r.r [c]	e.r [d]
**1**	**CuCl**	**>99%**	**70**	**92:08**	**87:13**
**2**	**CuBr·SMe_2**	**>99%**	**63**	**92:08**	**86:14**
**3**	**CuCN**	**>99%**	**47**	**94:06**	**90:10**
**4[e]**	**CuCN**	**>99%**	**62**	**94:06**	**92:08**
**5**	**$Cu(OTf)_2$**	**>99%**	**16**	**91:09**	**83:17**
**6**	**CuI**	**>99%**	**25**	**91:09**	**86:14**

[a] Conditions: Propellane (0.5 mmol), Grignard reagent (0.3 mmol), Allylic Phosphate (0.2 mmol), [Cu] (10 mol%) and solvent (2 ml). [b] Isolated yield. [c] rr: (Branched:Linear), ratio was determined by GC-MS. [d] er was determined by SFC [e] L1* instead of L4*

5.5 Screening of reagents stoichiometry

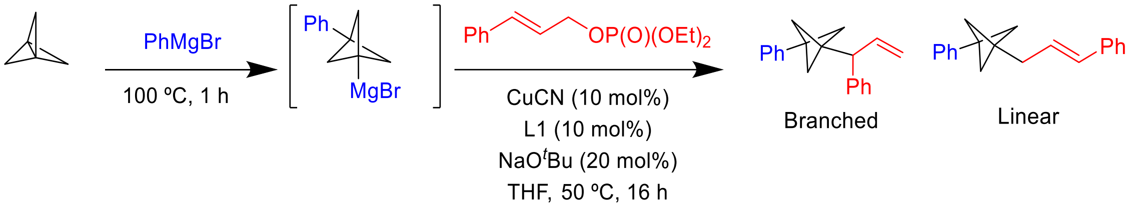

Entry [a]	Equiv. Phosphate	Equiv. PhMgBr	Equiv.Propellane	Yield [b]
**1**	**1**	**1,5**	**2,5**	**47**
**2**	**2**	**1**	**1,67**	**-**
**3**	**1**	**1**	**1**	**25**
**4**	**1**	**2,5**	**2,5**	**70**
**5**	**1**	**2**	**2**	**50**

[a] Conditions: [Cu] (10 mol%) and solvent (2 ml). [b] Determined by ^{1}H NMR analysis of reaction crude mixture using trimethyl benzene-1,3,5-tricarboxilate as an internal standard.

5.6 Catalyst Loading

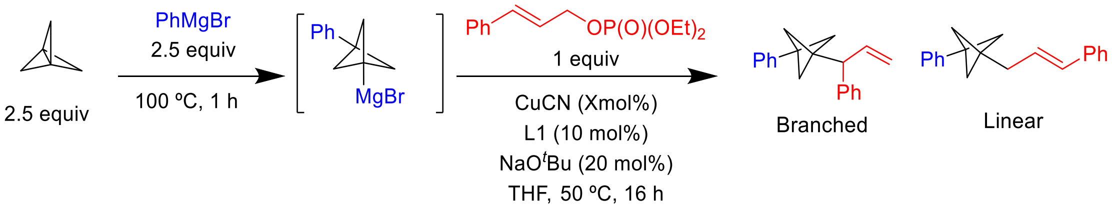

Entry [a]	Mol % copper	Conv [b]	Yield [b]	r.r [c]	e.r [d]
**1**	**10**	**>99%**	**70**	**95:05**	**92:08**
**2**	**5**	**>99%**	**63**	**95:05**	**85:15**
**3**	**0**	**>99%**	**65**	**>99:01**	**97:03**
**4[e]**	**0**	**>99%**	**10**	**10:90**	**-**

[a] Conditions: Propellane (0.5 mmol), Grignard reagent (0.5 mmol), Allylic Phosphate (0.2 mmol), and solvent (2 ml). [b] Isolated yield. [c] rr: (Branched:Linear), ratio was determined by GC-MS. [d] er was determined by SFC. [e] Ligand was not used.

6. Optimization of the regio- and enantioselective synthesis of chiral bicyclo[1.1.1]pentane derivatives via transition metal free asymmetric allylic substitution.

6.1 Proposed Reaction

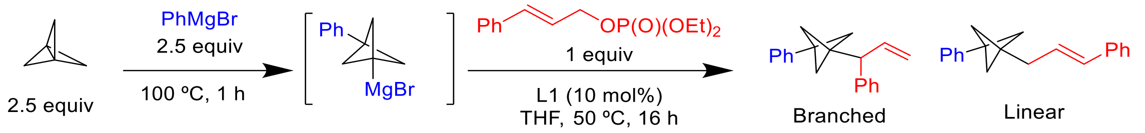

6.2 Screening of chiral Ligands

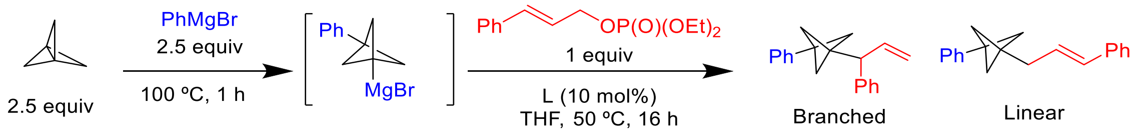

Entry [a]	Ligand	Conv [b]	Yield [b]	r.r [c]	e.r [d]
**1**	**L1**	**>99%**	**65**	**>99:01**	**97:03**
**2**	**L2**	**>99%**	**-**	**-**	**-**
**3**	**L3**	**>99%**	**44**	**50:50**	**73:27**
**4**	**L4**	**>99%**	**56**	**92:08**	**89:11**
**5**	**L5**	**>99%**	**38**	**83:17**	**50:50**

[a] Conditions: Propellane (0.5 mmol), Grignard reagent (0.5 mmol), Allylic Phosphate (0.2 mmol), Ligand (10 mol%) and solvent (2ml). [b] Isolated yield [c] rr: (Branched:Linear), ratio was determined by GC-MS. [d] er was determined by SFC.

6.3 Screening of solvents

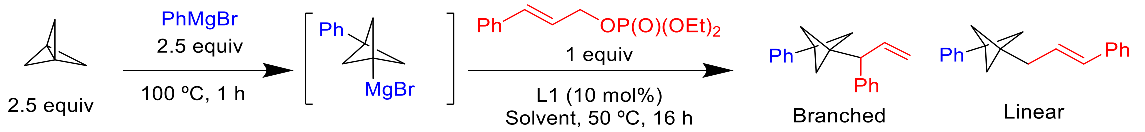

Entry [a]	Solvent	Conv [b]	Yield	r.r [c]	e.r [d]
**1**	**1,4-Dioxane**	**>99%**	**45**	**91:09**	**72:28**
**2**	**Diethyl ether**	**>99%**	**55**	**90:10**	**78:22**
**3**	**THF**	**>99%**	**65**	**>99:01**	**97:03**

[a] Conditions: Propellane (0.5 mmol), Grignard reagent (0.5 mmol), Allylic Phosphate (0.2 mmol), Ligand (10 mol%) and solvent (2ml). [b]Isolated yield. [c] rr: (Branched:Linear), ratio was determined by GC-MS. [d] er was determined by SFC.

6.4 Screening of Leaving group

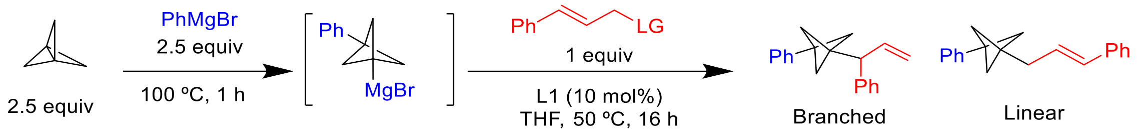

Entry[a]	LG	Conv [b]	Yield	r.r [c]	e.r [d]
**1**	**$OPO(OEt)_2$**	**>99%**	**65**	**>99:01**	**97:03**
**2**	**Br**	**>99%**	**67**	**92:08**	**72:28**

[a] Conditions: Propellane (0.5 mmol), Grignard reagent (0.5 mmol), Allylic Phosphate (0.2 mmol), Ligand (10 mol%) and solvent (2 ml). [b]Isolated yield. [c] rr: (Branched:Linear), ratio was determined by GC-MS. [d] er was determined by SFC.

6.5 Screening of reagents stoichiometry

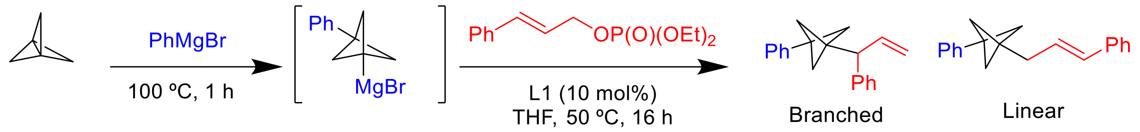

Entry	Equiv. Phosphate	Equiv. PhMgBr	Equiv.Propellane	Yield [a]
**1**	**1**	**1,5**	**1,5**	**45**
**2**	**2**	**1**	**1**	**-**
**3**	**1**	**1**	**1**	**20**
**4**	**1**	**2,5**	**2,5**	**65**

[a] Conditions: Ligand (10 mol%) and solvent (2 ml). [b] Determined by ^{1}H NMR analysis of reaction crude mixture using Trimethyl benzene-1,3,5-tricarboxilate as an internal standard.

6.6 Screening of temperature

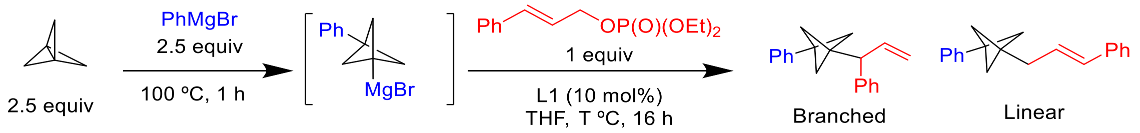

Entry [a]	T °C	Conv [b]	Yield	r.r [c]	e.r [d]
**1**	**30**	**>99%**	**59**	**85:15**	**91:09**
**2**	**50**	**>99%**	**65**	**>99:01**	**97:03**
**3**	**70**	**>99%**	**55**	**96:04**	**91:09**

[a] Conditions: Propellane (0.5 mmol), Grignard reagent (0.5 mmol), Allylic Phosphate (0.2 mmol), Ligand (10 mol%) and solvent (2 ml). [b] Isolated yield [c] rr: (Branched:Linear), ratio was determined by GC-MS. [d] er was determined by SFC.

6.7 NHC Loading

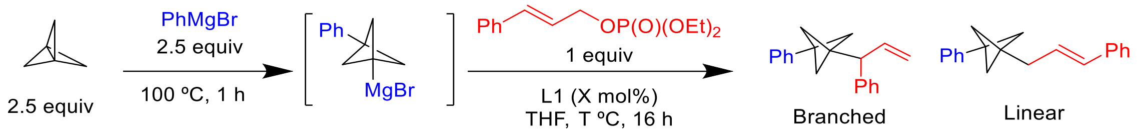

Entry [a]	L1 mol%	Conv [b]	Yield	r.r [c]	e.r [d]
**1**	**10**	**>99%**	**65**	**>99:01**	**97:03**
**2**	**6**	**>99%**	**65**	**>99:01**	**97:03**
**3**	**3**	**>99%**	**50**	**98:02**	**84:16**

[a] Conditions: Propellane (0.5 mmol), Grignard reagent (0.5 mmol), Allylic Phosphate (0.2 mmol), Ligand (X mol%) and solvent (2 ml). [b] Isolated yield. [c] rr: (Branched:Linear), ratio was determined by GC-MS. [d] er was determined by SFC.

7. General procedure for the synthesis of allylic phosphates (General Procedure A)

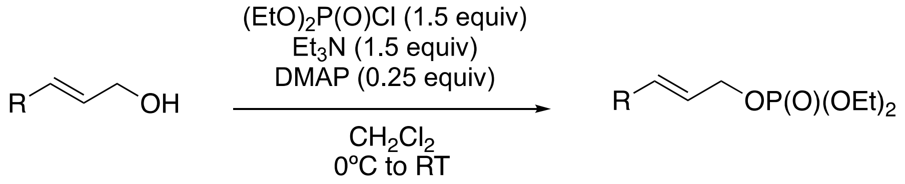

In a 100 mL flame-dried flask equipped with a magnetic bar was added DMAP (2.5 mmol, 0.25 equiv). The flask was then evacuated and backfilled with argon three times. Dry CH_2Cl_2 (0.1M), Et_3N (2.0 equiv) and allylic alcohol (10 mmol, 1.0 equiv) were added in turn to the flask. The reaction mixture was cooled to 0 °C and was stirred for 1 h then diethyl chlorophosphate (10 mmol, 1.0 equiv) was added dropwise to the solution. After completion (TLC monitoring), the reaction was carefully quenched with saturated aqueous NH_4Cl solution, and the aqueous phase extracted with CH_2Cl_2 (×3). Combined organic phases were dried over anhydrous Na_2SO_4 and filtered. The solvent was removed under reduced pressure. Crude product was purified through flash column chromatography using the indicated mixture of solvents as eluent.

Diethyl (*E*)-(3-(tetrahydro-*2H*-pyran-4-yl)allyl)phosphonate (25)

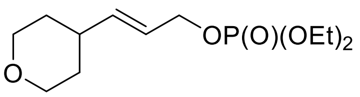

Synthesized from (*E*)-3-(tetrahydro-*2H*-pyran-4-yl)prop-2-en-1-ol according to general procedure A. Colorless oil obtained after column chromatography (Hexane:AcOEt 6:4). **^{1}H NMR** (500 MHz, $CDCl_3$) δ 5.65 (dd, *J* = 15.5, 6.3 Hz, 1H), 5.54 – 5.46 (m, 1H), 4.39 (t, *J* = 8.4 Hz, 2H), 4.06 – 3.96 (m, 4H), 3.86 (dd, *J* = 11.7, 4.5 Hz, 2H), 3.31 (td, *J* = 11.7, 2.1 Hz, 2H), 2.14 (ddq, *J* = 15.5, 6.8, 4.1 Hz, 1H), 1.58 – 1.45 (m, 2H), 1.42 – 1.30 (m, 2H), 1.23 (t, *J*= 7,1, 6H). **^{13}C NMR** (126 MHz, $CDCl_3$) δ 135.4, 125.7 (d, *J* = 6.4 Hz), 68.3 (d, *J* = 5.4 Hz), 63.8 (d, *J* = 5.9 Hz), 41.6, 28.2, 22.4, 16.3 (d, *J* = 6.8 Hz). **^{31}P NMR** (202 MHz, $CDCl_3$) δ 0.2. **HRMS (APCI)** Calc. for $C_{12}H_{24}O_5P$ [M+H$^+$] 279.1356; found 279.1358.

8. General Procedure for the regio- and enantioselective synthesis of α-chiral bicyclo[1.1.1]pentanes (General Procedure B)

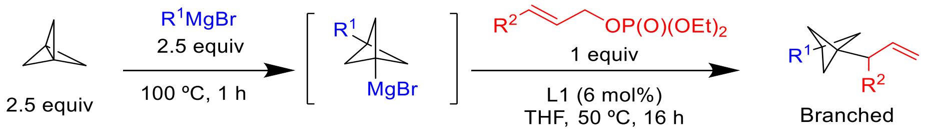

In a sealed tube, the corresponding Grignard reagent (0.5 mmol, 2.5 equiv) and the solution of [1.1.1]propellane in diethyl ether (0.5 mmol, 2.5 equiv) were added at room temperature and under inert atmosphere. The sealed tube was sealed and heated at 100 °C in an oil bath with stirring for 1 hour. It was allowed to cool, and the solvent was removed under high vacuum (about 30 minutes and making sure it was all evaporated). Ligand **L1** (0.012 mmol, 0.06 equiv) was dissolved in a vial with THF (1 ml) under inert atmosphere and was added to the sealed tube. The corresponding allylic phosphate (0.2 mmol, 1 equiv) was dissolved in THF (1 ml) and added to the sealed tube and stirred overnight at 50 °C in an oil bath.

Then, the mixture was diluted with CH_2Cl_2 (5 ml) and washed with saturated aqueous solution of NH_4Cl (2x5 ml). The aqueous layer was extracted with CH_2Cl_2 (5 ml). Combined organic layers were dried over anhydrous Na_2SO_4 and filtered. The solvent was removed under reduced pressure and the crude product was purified through flash column chromatography using the indicated mixture of solvents as eluent.

Note: Racemic products were synthesized by using IMesCuCl instead of L1. In that case, after evaporation of the Et_2O, IMesCuCl was dissolved in a vial with THF (1 ml) under inert atmosphere and was added to the sealed tube. The corresponding allylic phosphate was was dissolved in THF (1 ml) and added to the sealed tube and stirred overnight at 50 °C in an oil bath.

The substrates on which the enantiomeric excess could not be measured directly were derivatized by olefin metathesis or hydroboration/oxidation for subsequent analysis (see sections 10 and 11).

9. Product characterization

(*R*)-1-Phenyl-3-(1-phenylallyl)bicyclo[1.1.1]pentane (4)

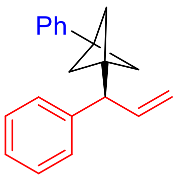

Synthesized from **1**, **2** and **3** according to general procedure B. Colorless oil obtained in 65% yield with >99:1 rr and 97:3 er after column chromatography (Pentane 100%). **^{1}H NMR** (500 MHz, $CDCl_3$) δ 7.34 – 7.30 (m, 2H), 7.29 – 7.27 (m, 2H), 7.24 – 7.22 (m, 1H), 7.21 – 7.16 (m, 5H), 6.13 (ddd, *J* = 17.0, 10.3, 8.3 Hz, 1H), 5.17 – 5.08 (m, 2H), 3.50 (d, *J* = 8.3 Hz, 1H), 1.93 – 1.77 (m, 6H). **^{13}C NMR** (126 MHz, $CDCl_3$) 142.2, 141.4, 138.5, 128.4, 128.2 (2C), 128.1 (2C), 126.4 (2C), 126.4 (2C), 126.3, 115.9, 52.0, 51.1 (3C), 42.6, 41.8. **HRMS (APCI)** Calc. for $C_{20}H_{21}$ [M+H$^+$] 261.1638, found 261.1641. **Optical rotation:** $[\alpha]_D^{21}$+ 2.7 (c=0.66, $CHCl_3$).

Enantiomeric purity was determined by chiral SFC analysis [Lux i-Amylose-1, 100 bar, T_{oven}: 40 °C, Flow: 0.6 mL/min; 1% MeOH, λ =220 nm, major enantiomer t_R = 19.30 min, minor enantiomer t_R = 17.33 min].

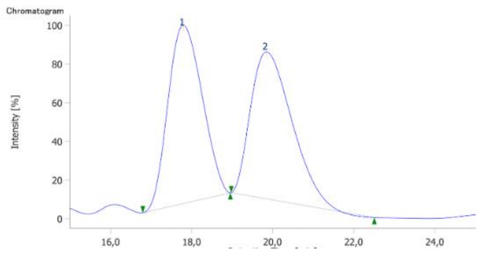

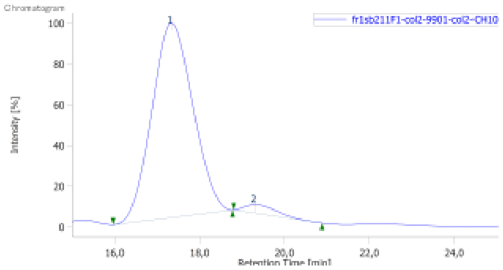

Peak Information

#	Peak Name	CH	tR [min]	Area [µV·sec]	Area%
1	Unknown	10	17.793	13688661	49.896
2	Unknown	10	19.847	13745590	50.104

Peak Information

#	Peak Name	CH	tR [min]	Area [µV·sec]	Area%
1	Unknown	10	17.333	14375161	96.693
2	Unknown	10	19.303	491672	3.307

(*R*)-1-(1-(3-Chlorophenyl)allyl)-3-phenylbicyclo[1.1.1]pentane (5)

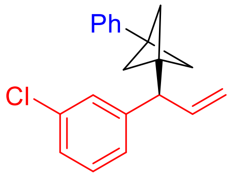

Synthesized from **1**, **2** and **45** according to general procedure B. Colorless oil obtained in 79% yield with >99:1 rr and 89:11 er after column chromatography (Pentane 100%). **^{1}H NMR** (500 MHz, $CDCl_3$) δ 7.34 – 7.27 (m, 3H), 7.26 – 7.20 (m, 5H), 7.19-7.10 (m, 1H), 6.16 (ddd, *J* = 17.1, 10.3, 8.3 Hz, 1H), 5.21 (d, *J* = 10.3 Hz, 1H), 5.15 (d, *J* = 17.1, 1H), 3.52 (d, *J* = 8.3 Hz, 1H), 1.96 – 1.89 (m, 6H). **^{13}C NMR** (126 MHz, $CDCl_3$) δ 144.2, 141.1, 137.7, 134.2, 129.7, 128.2, 128.2, 126.6 (2C), 126.5 (2C), 126.4, 126.1, 116.6, 51.6, 51.1(3C), 42.6, 41.7. **HRMS (APCI)** Calc. for $C_{20}H_{20}Cl$ [M+H$^+$] 295.1248, found 295.1252. **Optical rotation:** $[\alpha]_D^{21}$+ 3.9 (c=0.57, $CHCl_3$).

Enantiomeric purity was determined by chiral SFC analysis [Lux i-Amylose-1, 100 bar, T_{oven}: 40 °C, Flow: 0.6 mL/min; 1% MeOH, λ =220 nm, major enantiomer t_R = 22.89 min, minor enantiomer t_R = 18.66 min].

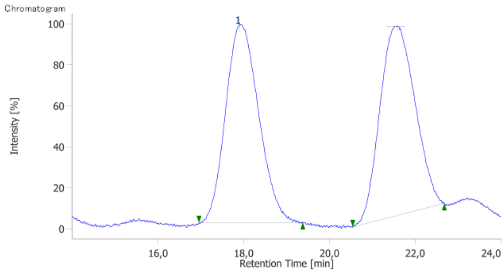

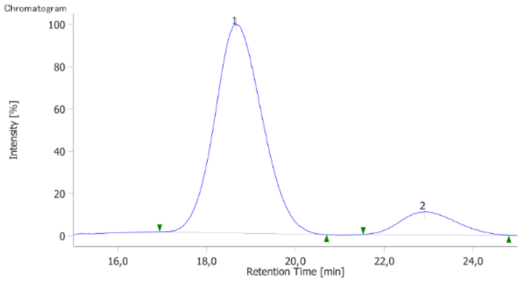

Peak Information

#	Peak Name	CH	tR [min]	Area [µV·sec]	Area%
1	Unknown	10	17.897	329441	49.624
2	Unknown	10	21.577	334437	50.376

Peak Information

#	Peak Name	CH	tR [min]	Area [µV·sec]	Area%
1	Unknown	10	18.663	7619774	88.506
2	Unknown	10	22.893	989583	11.494

Note: the other peak in racemic chromatogram corresponds to the linear regioisomer.

(*R*)-1-Phenyl-3-(1-(4-(trifluoromethyl)phenyl)allyl)bicyclo[1.1.1]pentane (6)

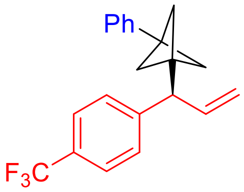

Synthesized from **1**, **2** and **46** according to general procedure B. Colorless oil obtained in 68% yield with 98:2 rr and 98:2 er after column chromatography (Pentane 100%). **^{1}H NMR** (500 MHz, $CDCl_3$) δ 7.45 (d, *J* = 8.3 Hz, 2H), 7.19 – 7.12 (m, 4H), 7.08 – 7.02 (m, 3H), 5.98 (ddd, *J* = 17.1, 10.3, 8.3 Hz, 1H), 5.05 (d, *J* = 10.3 Hz, 1H), 4.98 (d, *J* = 17.1 Hz, 1H), 3.44 (d, *J* = 8.3 Hz, 2H), 1.78 – 1.71 (m, 6H). **^{13}C NMR** (126 MHz, $CDCl_3$) δ 146.2, 141.0, 137.5, 128.9 (q, *J* = 30.4 Hz), 128.4 (2C), 128.3 (2C), 126.6, 126.1 (2C), 125.4 (q, *J* = 3.9 Hz, 2C), 124.4 (q, *J* = 271.4 Hz) 116.8, 51.8, 51.1 (3C), 42.7, 41.5. **^{19}F NMR** (282 MHz, $CDCl_3$) δ -67.1. **HRMS (APCI)** Calc. for $C_{21}H_{20}F_3$ [M+H$^+$] 329.1512, found 329.1503. **Optical rotation:** $[\alpha]_D^{21}$+ 7.3 (c=0.54, $CHCl_3$).

Enantiomeric purity was determined by chiral SFC analysis [Lux i-Cellulose-1, 100 bar, T_{oven}: 40 °C, Flow: 1 mL/min; 1% MeOH, λ =220 nm, major enantiomer t_R = 23.60 min, minor enantiomer t_R = 22.13 min].

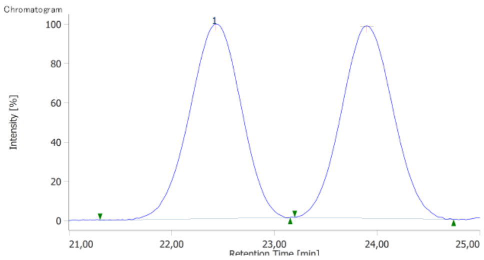

Peak Information

#	Peak Name	CH	tR [min]	Area [μV sec]	Area%
1	Unknown	10	22.430	3662373	50.285
2	Unknown	10	23.897	3620821	49.715

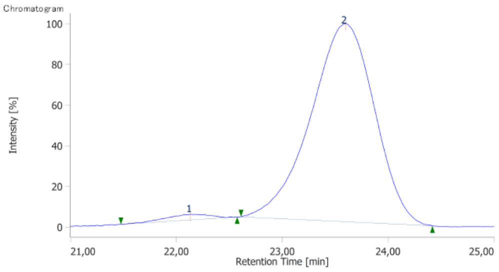

Peak Information

#	Peak Name	CH	tR [min]	Area [μV sec]	Area%
1	Unknown	10	22.133	47365	1.943
2	Unknown	10	23.597	2390167	98.057

(*R*)-1-Phenyl-3-(1-(p-tolyl)allyl)bicyclo[1.1.1]pentane (7)

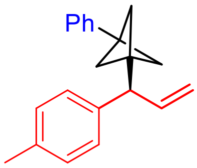

Synthesized from **1**, **2** and **47** according to general procedure B. Yellow oil obtained in 94% yield with >99:1 rr and 83:17 er after column chromatography (Pentane 100%). **^{1}H NMR** (500 MHz, CDCl3) δ 7.31 – 7.27 (m, 2H), 7.21 – 7.17 (m, 3H), 7.16 – 7.12 (m, 2H), 7.11 – 7.08 (m, 2H), 6.20 – 6.06 (m, 1H), 5.16 – 5.06 (m, 2H), 3.47 (d, *J* = 8.3, 1H), 2.35 (s, 3H), 1.92 – 1.85 (m, 6H).**^{13}C NMR** (126 MHz, $CDCl_3$) δ 141.5, 139.1, 138.7, 135.8, 129.1 (2C), 128.2 (2C), 128.0 (2C), 126.4, 126.1 (2C), 115.8, 51.6, 51.1 (3C), 42.5, 41.8, 21.2. **HRMS (APCI)** Calc. for $C_{21}H_{23}$ [M+H$^+$] 275.1794, found 275.1800. **Optical rotation:** $[\alpha]_D^{21}$+ 2.93 (c=1, $CHCl_3$).

Enantiomeric purity was determined by chiral SFC analysis [Lux i-Amylose-1, 100 bar, T_{oven}: 40 °C, Flow: 0.6 mL/min; 1% MeOH, λ =220 nm, major enantiomer t_R = 16.40 min, minor enantiomer t_R = 18.43 min].

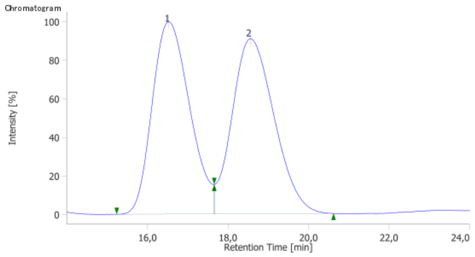

Peak Information

#	Peak Name	CH	tR [min]	Area [μV·sec]	Area%
1	Unknown	10	16,523	4458785	49,509
2	Unknown	10	18,550	4547202	50,491

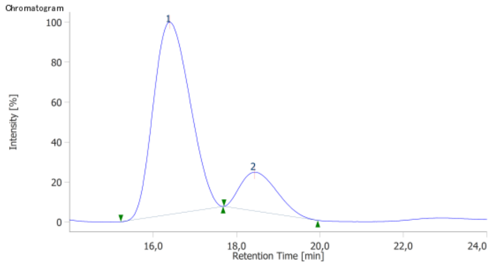

Peak Information

#	Peak Name	CH	tR [min]	Area [μV·sec]	Area%
1	Unknown	10	16,397	15173725	83,098
2	Unknown	10	18,430	3086212	16,902

(*R*)-1-(1-(2-Fluorophenyl)allyl)-3-phenylbicyclo[1.1.1]pentane (8)

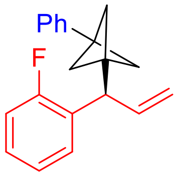

Synthesized from **1**, **2** and **48** according to general procedure B. Colorless oil obtained in 80% yield with >99:01 rr and 84:16 er after column chromatography (Pentane 100%). **^{1}H NMR** (500 MHz, $CDCl_3$) δ 7.30 – 7.26 (m, 2H), 7.24 – 7.21 (m, 2H), 7.20 – 7.17 (m, 3H), 7.16-7.10 (m, 1H), 7.09-7.04 (m, 1H), 6.16 (dddd, *J* = 16.9, 10.2, 8.4, 1.5 Hz, 1H), 5.19 – 5.14 (m, 2H), 3.92 (d, *J* = 8.4 Hz, 1H), 1.94 – 1.86 (m, 6H). **^{13}C NMR** (126 MHz, $CDCl_3$) δ 160.7 (d, *J* = 244.8 Hz), 141.3, 137.2, 129.4 (d, *J* = 5.0 Hz), 129.0 (d, *J* = 15.0 Hz), 128.2 (2C), 127.8 (d, *J* = 8.2 Hz), 126.4, 126.1 (2C), 124.0 (d, *J* = 3.2 Hz), 116.6, 115.6 (d, *J* = 22.7 Hz), 51.2 (3C), 44.4 (d, *J* = 2.3 Hz), 42.3 (d, *J* = 1.2 Hz), 41.3. **^{19}F NMR** (282 MHz, $CDCl_3$) δ -114.0. **HRMS (APCI)** Calc. for $C_{20}H_{20}F$ [M+H$^+$] 279.1544, found 279.1547. **Optical rotation:** $[\alpha]_D^{21}$+ 4.1 (c=0.62, $CHCl_3$).

Enantiomeric purity was determined by chiral SFC analysis [Lux i- Amylose-1, 100 bar, T_{oven}: 40 °C, Flow: 0.6 mL/min; 1% MeOH, λ =220 nm, major enantiomer t_R = 21.56 min, minor enantiomer t_R = 23.10 min].

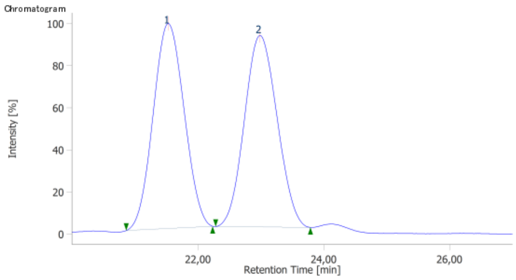

Peak Information

#	Peak Name	CH	tR [min]	Area [μV·sec]	Area%
1	Unknown	10	21,527	3516822	50,351
2	Unknown	10	22,987	3467726	49,649

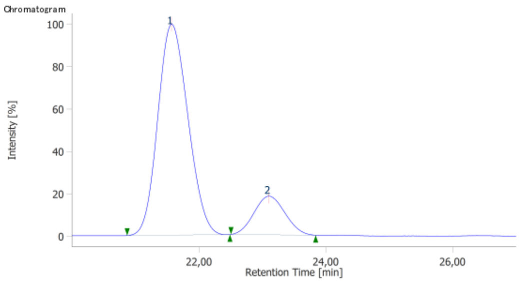

Peak Information

#	Peak Name	CH	tR [min]	Area [μV·sec]	Area%
1	Unknown	10	21,567	6942842	84,152
2	Unknown	10	23,103	1307556	15,848

(*R*)-1-(1-(Naphthalen-2-yl)allyl)-3-phenylbicyclo[1.1.1]pentane (9)

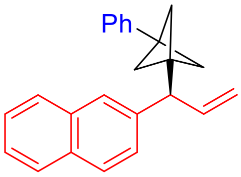

Synthesized from **1**, **2** and **49** according to general procedure B. Yellow oil obtained in 93% yield with >99:1 rr and 87:13 er after column chromatography (Pentane 100%). **^{1}H NMR** (500 MHz, $CDCl_3$) δ 7.90 – 7.83 (m, 3H), 7.71 – 7.67 (m, 1H), 7.55 – 7.47 (m, 2H), 7.41 (d, *J* = 8.5 Hz, 1H), 7.34 – 7.29 (m, 2H), 7.25 – 7.20 (m, 3H), 6.34 – 6.25 (m, 1H), 5.25 – 5.18 (m, 2H), 3.73 (d, *J* = 8.1 Hz, 1H), 2.02 – 1.93 (m, 6H).**^{13}C NMR** (126 MHz, $CDCl_3$) δ 141.2, 139.7, 138.2, 133.7, 132.3, 128.1 (2C), 127.8, 127.7, 127.6, 126.8, 126.3, 126.3, 126.0 (2C), 125.9, 125.4, 116.2, 51.9, 51.2 (3C), 42.5, 41.8 **HRMS (APCI)** Calc. for $C_{24}H_{23}$ [M+H$^+$] 311.1794, found 311.1790. **Optical rotation:** $[\alpha]_D^{21}$+ 33.6 (c=0.59, $CHCl_3$).

Enantiomeric purity was determined by chiral uHPLC analysis [Chiralpak IB N-3, T_{oven}: 40 °C, Flow: 1 mL/min; 100% Hexane, λ =225.5 nm, major enantiomer t_R = 3.99 min, minor enantiomer t_R = 3.17 min].

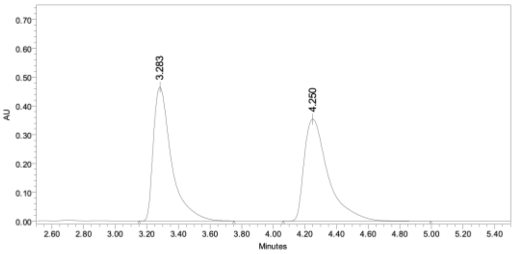

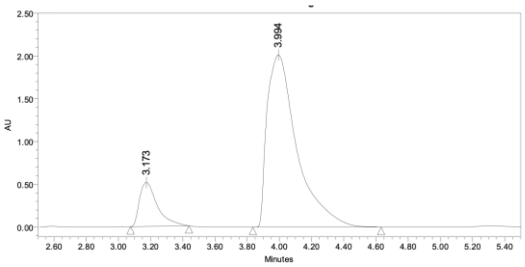

Peak Results

	RT	Area	% Area	Height
1	3.283	3703542	49.98	464466
2	4.250	3705820	50.02	353580

Peak Results

	RT	Area	% Area	Height
1	3.173	3953592	13.23	514042
2	3.994	25919081	86.77	2012002

(*R*)-1-(4-Methylpent-1-en-3-yl)-3-phenylbicyclo[1.1.1]pentane (10)

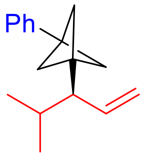

Synthesized from **1**, **2** and **50** according to general procedure B. Yellow oil obtained in 66% yield with 99:1 rr and 98:2 er after column chromatography (Pentane 100%). **^{1}H NMR** (500 MHz, $CDCl_3$) δ 7.34 – 7.27 (m, 2H), 7.23 – 7.16 (m, 3H), 5.63 (dt, *J* = 17.0, 10.0 Hz, 1H), 5.06 (dd, *J* = 10.2, 2.3 Hz, 1H), 4.98 (ddd, *J* = 17.1, 2.4, 0.7 Hz, 1H), 1.99 (dd, *J* = 9.9, 5.0 Hz, 1H), 1.96 – 1.91 (m, 6H), 1.85-1.78 (m, 1H), 0.94 (d, *J* = 6.7 Hz, 3H), 0.88 (d, *J* = 6.9 Hz, 3H).**^{13}C NMR** (126 MHz, $CDCl_3$) δ 140.5, 136.5, 127.1 (2C), 125.2, 125.0 (2C), 115.1, 50.9, 50.8, 41.1 (3C), 40.0, 28.7, 21.1, 18.1. **HRMS (APCI)** Calc. for $C_{17}H_{23}$ [M+H$^+$] 227.1794, found 227.1794. **Optical rotation:** $[\alpha]_D^{20}$- 72.6 (c=0.5, $CHCl_3$).

Enantiomeric ratio was determined on derivatization product **25** (see section 10).

(*R*)-1-(1-Cyclohexylallyl)-3-phenylbicyclo[1.1.1]pentane (11)

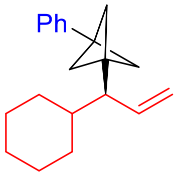

Synthesized from **1**, **2** and **51** according to general procedure B. Colorless oil obtained in 89% yield with >99:1 rr and 97:3 er after column chromatography (Pentane 100%). **^{1}H NMR** δ 7.33 – 7.28 (m, 2H), 7.25 – 7.19 (m, 3H), 5.64 (dt, *J* = 17.1, 10.0 Hz, 1H), 5.04 (dd, *J* = 10.0, 2.3 Hz, 1H), 4.95 (ddd, *J* = 17.1, 2.3, 0.8 Hz, 1H), 1.99 (dd, J = 9.9, 5.1 Hz, 1H), 1.96 – 1.91 (m, 6H), 1.79 – 1.66 (m, 5H), 1.49 – 1.40 (m, 1H), 1.33 – 1.21 (m, 2H), 1.16 – 1.08 (m, 2H), 0.98 (m, 1H). **^{13}C NMR** (126 MHz, $CDCl_3$) δ 141.6, 137.7, 128.5 (2C), 126.7, 126.3 (2C), 116.8, 68.6, 68.5, 52.4 (3C), 51.3, 42.8, 40.9, 37.6, 32.5, 30.7(2C). **HRMS (APCI)** Calc. for $C_{20}H_{27}$ [M+H$^+$] 267.2107, found 267.2114. **Optical rotation:** $[\alpha]_D^{21}$- 83.8 (c=0.58, $CHCl_3$).

Enantiomeric purity was determined by chiral SFC analysis [Lux i-Amylose-1, 100 bar, T_{oven}: 40 °C, Flow: 0.8 mL/min; 1% MeOH, λ =220 nm, major enantiomer t_R = 16.47 min, minor enantiomer t_R = 18.84 min].

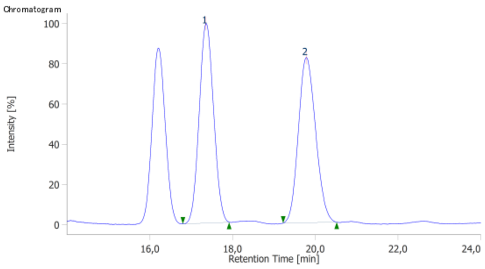

Peak Information

#	Peak Name	CH	tR [min]	Area [µV·sec]	Area%
1	Unknown	10	17,357	929755	50,150
2	Unknown	10	19,780	924193	49,850

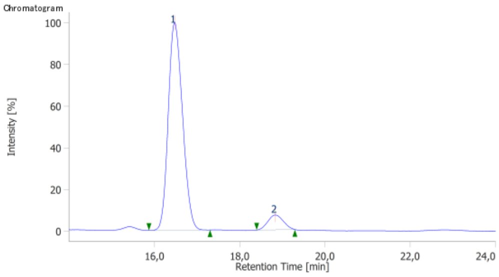

Peak Information

#	Peak Name	CH	tR [min]	Area [µV·sec]	Area%
1	Unknown	10	16,470	3947459	92,795
2	Unknown	10	18,837	306497	7,205

Note: the other peak in racemic chromatogram corresponds to the linear regioisomer.

(*R*)-4-(1-(3-Phenylbicyclo[1.1.1]pentan-1-yl)allyl)tetrahydro-2H-pyran (12)

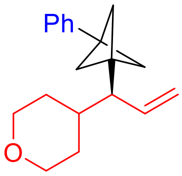

Synthesized from **1**, **2** and **52** according to general procedure. Yellow oil obtained in 66% yield with 99:1 rr and 98:2 er after column chromatography (Pentane 100%). **^{1}H NMR** (500 MHz, $CDCl_3$) δ 7.41 – 7.24 (m, 2H), 7.23 – 7.15 (m, 3H), 5.58 (dt, *J* = 17.0, 10.0 Hz, 1H), 5.07 (dd, *J* = 10.0, 2.2 Hz, 1H), 4.97 (ddd, *J* = 17.1, 2.2, 0.7 Hz, 1H), 4.01 – 3.91 (m, 2H), 3.44-3.33 (m, 2H), 2.00 (dd, *J* = 9.9, 6.0 Hz, 1H), 1.93 (s, 6H), 1.68 – 1.60 (m, 3H), 1.50 – 1.45 (m, 1H), 1.37 – 1.27 (m, 1H).**^{13}C NMR** (126 MHz, $CDCl_3$). δ 141.4, 137.5, 128.2 (2C), 126.4, 126.1 (2C), 116.6, 68.4, 68.3, 52.2 (3C), 51.0, 42.5, 40.6, 37.4, 32.2, 30.4. **HRMS (APCI)** Calc. for $C_{19}H_{25}O$ [M+H$^+$] 269.1900, found 269.1901. **Optical rotation**: $[\alpha]_D^{21}$ - 83.2 (c=0.52, $CHCl_3$).

Enantiomeric purity was determined by chiral uHPLC analysis [Chiralpak IB N-3, T_{oven}: 40 °C, Flow: 0.8 mL/min; 99:01 Hexane: *i*PrOH, λ =220.7 nm, major enantiomer t_R = 9.59 min, minor enantiomer t_R = 10.15 min].

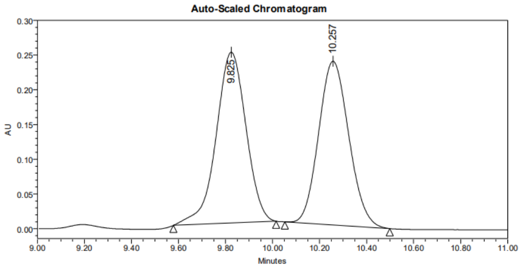

Peak Results

	RT	Area	% Area	Height
1	9.825	2172257	50.83	245957
2	10.257	2101644	49.17	235754

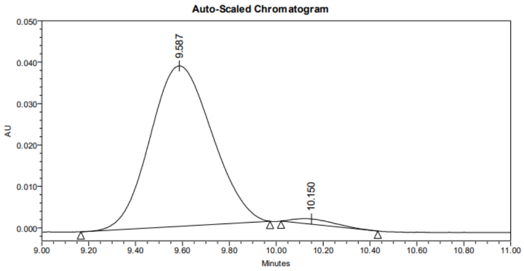

Peak Results

	RT	Area	% Area	Height
1	9.587	712277	97.88	38717
2	10.150	15413	2.12	1278

(*R*)-1-(But-3-en-2-yl)-3-phenylbicyclo[1.1.1]pentane (13)

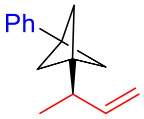

Synthesized from **1**, **2** and **53** according to general procedure B. Yellow oil obtained in 66% yield with 99:1 rr and 81:19 er after column chromatography (Pentane 100%). **^{1}H NMR** (500 MHz, $CDCl_3$) δ 7.31 – 7.28 (m, 2H), 7.23 – 7.18 (m, 3H), 5.74 (ddd, *J* = 17.2, 10.3, 7.4 Hz, 1H), 5.03 – 4.97 (m, 2H), 2.40-2.30 (m, 1H), 1.85 (s, 6H), 1.00 (d, J = 6.9 Hz, 3H).**^{13}C NMR** (126 MHz, $CDCl_3$) δ 141.7, 141.3, 128.2 (2C), 126.4, 126.2 (2C), 113.5, 50.2 (3C), 42.0, 41.6, 38.7, 16.1. **HRMS (APCI)** Calc. for $C_{15}H_{19}$ [M+H^{+}] 199.1481, found 199.1479. **Optical rotation:** $[\alpha]_D^{21}$- 11.7 (c=0.5, $CHCl_3$).

Enantiomeric ratio was determined on derivatization product **26** (see section 10).

(*R*)-1-(Hex-1-en-3-yl)-3-phenylbicyclo[1.1.1]pentane (14)

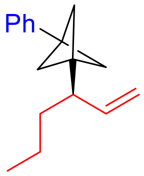

Synthesized from **1**, **2** and **54** according to general procedure B. Colorless oil obtained in 73% yield with 97:3 rr and 84:16 er after column chromatography (Pentane 100%). **^{1}H NMR** (500 MHz, $CDCl_3$) δ 7.34 – 7.26 (m, 2H), 7.26 – 7.15 (m, 3H), 5.52 (ddd, *J* = 17.0, 10.2, 9.1 Hz, 1H), 5.12 – 4.93 (m, 2H), 2.20 – 2.09 (m, 1H), 1.88 – 1.82 (m, 6H), 1.46 – 1.32 (m, 2H), 1.30 – 1.16 (m, 2H), 0.90 (t, *J* = 6.9 Hz, 3H). **^{13}C NMR** (126 MHz, $CDCl_3$) δ 140.6, 139.0, 127.0 (2C), 125.2, 125.0 (2C), 114.0, 49.5 (3C), 43.9, 40.7, 40.3, 32.3, 19.6, 13.1. **HRMS (APCI)** Calc. for $C_{17}H_{22}$ [M^{+}] 226.1716, found 226.1714. **Optical rotation**: $[\alpha]_D^{20}$ - 35.9 (c=0.76, $CHCl_3$).
Enantiomeric ratio was determined on derivatization product **32** (see section 11).

(*R*)-1-(Dec-1-en-3-yl)-3-phenylbicyclo[1.1.1]pentane (15)

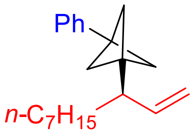

Synthesized from **1**, **2** and **55** according to general procedure B. Yellow oil obtained in 97% yield with >99:1 rr and 82:18 er after column chromatography (Pentane 100%). **^{1}H NMR** (500 MHz, CDCl3 δ 7.33 – 7.27 (m, 2H), 7.24 – 7.19 (m, 3H), 5.54 (dt, *J* = 17.0, 9.6 Hz, 1H), 5.07 – 4.96 (m, 2H), 2.17 – 2.09 (m, 1H), 1.89 – 1.83 (m, 6H), 1.35 – 1.24 (m, 12H), 0.90 (t, *J* = 6.7 Hz, 3H).**^{13}C NMR** (126 MHz, $CDCl_3$) δ 141.7, 140.1, 128.1 (2C), 126.2, 126.0 (2C), 115.0, 50.5 (3C), 45.2, 41.8, 41.4, 31.9, 31.1, 29.6, 29.3, 27.6, 22.7, 14.1. **HRMS (APCI)** Calc. for $C_{21}H_{31}$ [M+H^{+}] 283.2420, found 283.2424. **Optical rotation:** $[\alpha]_D^{21}$ - 30.1 (c=1, $CHCl_3$).

Enantiomeric purity was determined by chiral SFC analysis [Lux i-Amylose-1, 100 bar, T_{oven}: 40 °C, Flow: 1 mL/min; 1% MeOH, λ =220 nm, major enantiomer t_R = 7.35 min, minor enantiomer t_R = 8.92 min].

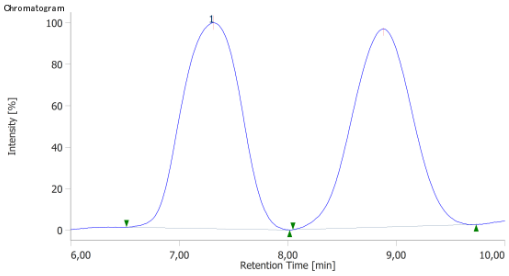

Peak Information

#	Peak Name	CH	tR [min]	Area [µV·sec]	Area%
1	Unknown	10	7,310	1442003	50,377
2	Unknown	10	8,880	1420424	49,623

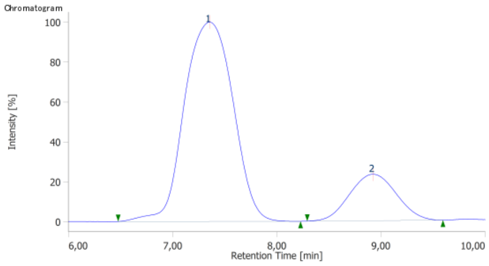

Peak Information

#	Peak Name	CH	tR [min]	Area [µV·sec]	Area%
1	Unknown	10	7,353	6648838	81,733
2	Unknown	10	8,923	1485989	18,267

(*R*)-1-(1-Phenylallyl)-3-(o-tolyl)bicyclo[1.1.1]pentane (16)

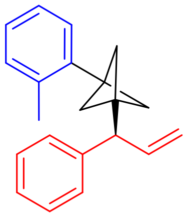

Synthesized from **1**, **34** and **3** according to general procedure. Yellow oil obtained in 95% yield with >99:1 rr and 93:7 er after column chromatography (Pentane 100%). **^{1}H NMR** (500 MHz, CDCl3) δ 7.40 – 7.35 (m, 2H), 7.30 – 7.23 (m, 3H), 7.18 – 7.09 (m, 4H), 6.24 – 6.15 (m, 1H), 5.22 – 5.13 (m, 2H), 3.55 (d, *J* = 8.5, 1H), 2.40 (s, 3H), 2.08 – 1.99 (m, 6H). **^{13}C NMR** (126 MHz, $CDCl_3$) δ 142.0, 138.5, 138.4, 136.9, 130.4, 128.3 (2C), 128.0 (2C), 127.7, 126.8 , 126.2 , 125.7, 115.9, 51.9, 51.0 (3C), 43.4, 42.9 , 20.7 . **HRMS (APCI)** Calc. for $C_{21}H_{23}$ [M+H$^+$] 275.1794, found 275.1798. **Optical rotation:** $[\alpha]_D^{21}$ + 44.1 (c=0.71, $CHCl_3$).

Enantiomeric purity was determined by chiral SFC analysis [Lux i-Amylose-1, 100 bar, T_{oven}: 40 °C, Flow: 0.6 mL/min; 1% MeOH, λ =220 nm, major enantiomer t_R = 40.01 min, minor enantiomer t_R = 43.32 min].

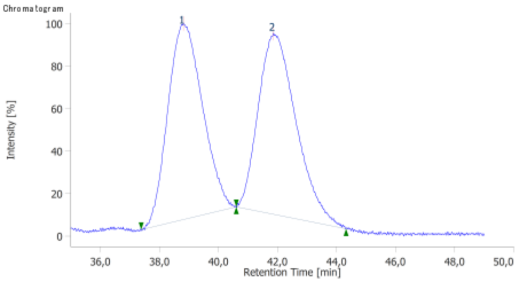

Peak Information

#	Peak Name	CH	tR [min]	Area [µV·sec]	Area%
1	Unknown	10	38,810	718642	49,812
2	Unknown	10	41,863	724060	50,188

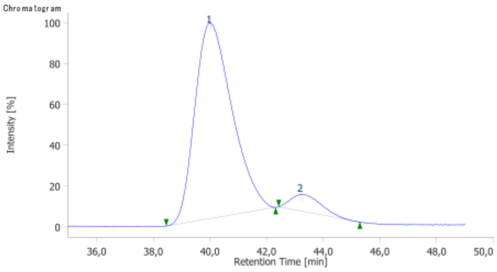

Peak Information

#	Peak Name	CH	tR [min]	Area [µV·sec]	Area%
1	Unknown	10	40,017	5691123	93,369
2	Unknown	10	43,230	404204	6,631

(*R*)-1-(4-Methylpent-1-en-3-yl)-3-(p-tolyl)bicyclo[1.1.1]pentane (17)

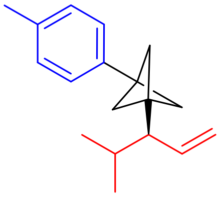

Synthesized from **1**, **35** and **50** according to general procedure B. Colorless oil obtained in 50 % yield with >99:1 rr and 91:9 er after column chromatography (Pentane 100%). **^{1}H NMR** (500 MHz, $CDCl_3$) δ 7.12 – 7.09 (m, 4H), 5.62 (dt, *J* = 17.0, 10.0 Hz, 1H), 5.05 (dd, *J* = 10.0, 2.3 Hz, 1H), 4.96 (ddd, *J* = 17.0, 2.3, 0.7 Hz, 1H), 2.32 (s, 3H), 1.97 (dd, *J* = 9.9, 5.0 Hz, 1H), 1.94 – 1.88 (m, 6H), 1.81 – 1.76 (m, 1H), 0.93 (d, *J* = 6.7 Hz, 3H), 0.87 (d, *J* = 6.7 Hz, 3H). **^{13}C NMR** (126 MHz, $CDCl_3$) δ 138.8, 137.7, 135.9, 128.9 (2C), 126.1 (2C), 116.2, 52.1 (3C), 51.9, 42.0, 41.1, 29.0, 22.3, 21.2, 19.3. **HRMS (APCI)** Calc. for $C_{18}H_{25}$ [$M+H^+$] 241.1951, found 241.1940. **Optical rotation**: $[\alpha]_D^{21}$+ 8.5 (c=0.62, $CHCl_3$).

Enantiomeric purity was determined by chiral uHPLC [Lux i-Amylose-3, T_{oven}: 40 °C, Flow: 0.4 mL/min; 100% Hexane, λ =223nm, major enantiomer t_R = 8.40 min, minor enantiomer t_R = 7.85 min].

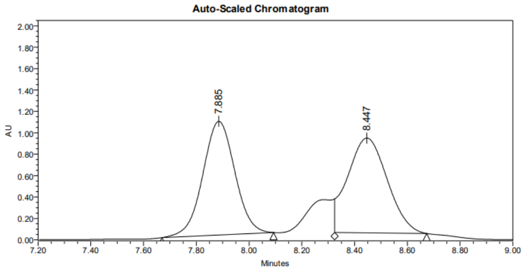

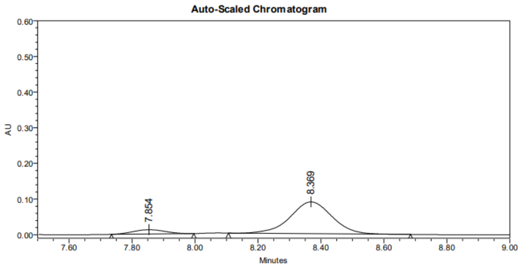

Peak Results

	RT	Area	% Area	Height
1	7.885	8988973	49.26	1062198
2	8.447	9260340	50.74	885736

Peak Results

	RT	Area	% Area	Height
1	7.854	84012	9.20	11970
2	8.369	828701	90.80	89276

Note: the other peak in racemic chromatogram corresponds to the linear regioisomer.

(*S*)-1-(4-Fluorophenyl)-3-(4-methylpent-1-en-3-yl)bicyclo[1.1.1]pentane (18)

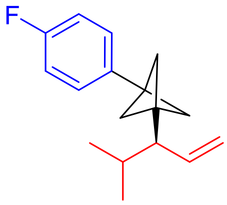

Synthesized from **1**, **36** and **50** according to general procedure B. Colorless oil obtained in 81% yield with >99:1 rr and 92.5:7.5 er after column chromatography (Pentane 100%). **^{1}H NMR** (500 MHz, $CDCl_3$) δ 7.16-7.12 (m, 2H), 6.98-6.92 (m, 2H), 5.61 (dt, *J* = 17.0, 10.0 Hz, 1H), 5.05 (dd, *J* = 10.0, 2.3 Hz, 1H), 4.97 (dd, *J* = 17.0, 2.3 Hz, 1H), 1.97 (dd, *J* = 10.0, 5.0 Hz, 1H), 1.93 – 1.90 (m, 6H), 1.83 – 1.74 (m, 1H), 0.93 (d, *J* = 6.8 Hz, 3H), 0.87 (d, *J* = 6.8 Hz, 3H). **^{13}C NMR** (126 MHz, $CDCl_3$) δ 161.6 (d, *J* = 243.8 Hz), 137.6, 137.6, 127.5 (d, *J* = 8.0 Hz,2C), 116.4, 114.8 (d, *J* = 21.3 Hz, 2C), 52.2 (3C), 51.9, 41.8, 41.2, 30.0, 22.3, 19.4. **^{19}F NMR-** (282 MHz, $CDCl_3$) δ -116.9. **HRMS (APCI)** Calc. for $C_{17}H_{22}F$ [$M+H^+$] 245.1700, found 245.1704. **Optical rotation**: $[\alpha]_D^{21}$ -31.9 (c=0.35, $CHCl_3$).
Enantiomeric ratio was determined on derivatization product **33** (see section 11).

(*R*)-1-(4-Methoxyphenyl)-3-(4-methylpent-1-en-3-yl)bicyclo[1.1.1]pentane (19)

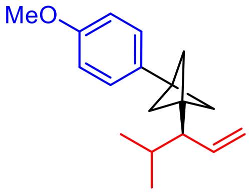

Synthesized from **1**, **37** and **50** according to general procedure B. Colorless oil obtained as a mixture 1:1 with the protonation of the Grignard-BCP in 70% yield with >99:1 rr and 98:02 er after column chromatography (Pentane 100%). **^{1}H NMR** (500 MHz, $CDCl_3$) δ 7.18 – 7.11 (m, 2H), 6.90 – 6.82 (m, 2H), 5.63 (dt, *J* = 17.0, 10.0 Hz, 1H), 5.06 (dd, *J* = 10.2, 2.4 Hz, 1H), 4.97 (dd, *J* = 17.0, 2.4 Hz, 1H), 3.79 (s, 3H), 1.98 (dd, *J* = 9.8, 4.9 Hz, 1H), 1.91 (s, 6H), 0.94 (d, *J* = 6.8 Hz, 3H), 0.88 (d, *J* = 6.8 Hz, 3H). **^{13}C NMR** (126 MHz, $CDCl_3$) δ 158.3, 137.7, 134.1, 127.1 (2C), 116.2, 113.7 (2C), 55.4, 52.3 (3C), 51.9, 41,8, 41.0, 29.9, 22.3, 19.3. **HRMS (APCI)** Calc. for $C_{18}H_{25}O$ [M+H^{+}] 257.1900, found 257.1901. **Optical rotation**: $[\alpha]_D^{21}$-28.6 (c=0.58, $CHCl_3$).

Enantiomeric purity was determined by chiral uHPLC [Lux i-Cellulose-5, T_{oven}: 40 °C, Flow: 1 mL/min; 100% Hexane, λ =227nm, major enantiomer t_R = 4.79 min, minor enantiomer t_R = 4.51 min].

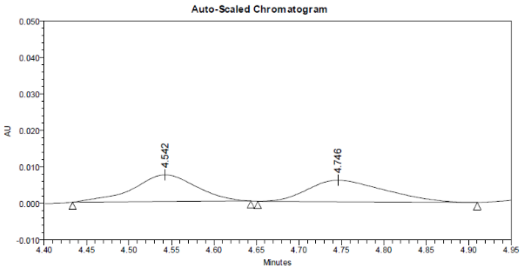

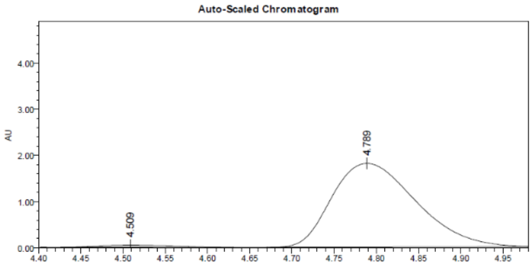

Peak Results

	RT	Area	% Area	Height
1	4.542	37838	50.65	7311
2	4.746	36869	49.35	5898

Peak Results

	RT	Area	% Area	Height
1	4.509	287695	2.24	45795
2	4.789	12558144	97.76	1812140

(*R*)-1-Ethyl-3-(1-phenylallyl)bicyclo[1.1.1]pentane (20)

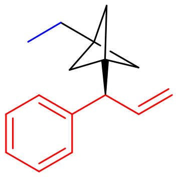

Synthesized from **1**, **38** and **3** according to general procedure B. Colorless oil obtained in 60% yield with >99:1 rr and 87:13 after column chromatography (Pentane 100%). Er was determined in derivatization product **51.** **^{1}H NMR** (500 MHz, $CDCl_3$) δ 7.34 – 7.27 (m, 2H), 7.24 – 7.17 (m, 1H), 7.17 – 7.11 (m, 2H), 6.08 (ddd, *J* = 17.1, 10.3, 8.3 Hz, 1H), 5.13 – 5.00 (m, 2H), 3.39 (d, *J* = 8.3 Hz, 1H), 1.45 – 1.40 (m, 6H), 1.38 – 1.35 (m, 2H), 0.79 (t, *J* = 7.4 Hz, 3H). **^{13}C NMR** (126 MHz, $CDCl_3$) δ 142.6, 138.9, 128.3 (2C), 128.1 (2C), 126.1, 115.5, 52.3, 48.5 (3C), 42.1, 41.5, 25.0, 10.7. **HRMS (APCI)** Calc. for $C_{16}H_{21}$ [M+H^{+}] 213.1638, found 213.1630. **Optical rotation**: $[\alpha]_D^{21}$+ 6.8 (c=0.81, $CHCl_3$).

Enantiomeric ratio was determined on derivatization product **27** (see section 10).

(*R*)-1-(3-(1-Phenylallyl)bicyclo[1.1.1]pentan-1-yl)hexane (21)

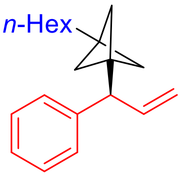

Synthesized from **1**, **39** and **3** according to general procedure B. Colorless oil obtained in 75% yield with >99:1 rr and 91:9 er after column chromatography (Pentane 100%). **^{1}H NMR** (500 MHz, $CDCl_3$) δ 7.32 – 7.27 (m, 2H), 7.22 – 7.17 (m, 1H), 7.16 – 7.11 (m, 2H), 6.07 (ddd, *J* = 17.1, 10.2, 8.3 Hz, 1H), 5.13 – 4.99 (m, 2H), 3.38 (d, *J* = 8.3 Hz, 1H), 1.46 – 1.38 (m, 6H), 1.29 – 1.15 (m, 10H), 0.87

(t, J = 7.0 Hz, 3H). **^{13}C NMR** (126 MHz, $CDCl_3$) δ 142.4, 138.8, 128.1 (2C), 128.0 (2C), 126.0, 115.3, 52.1, 49.0 (3C), 42.2, 40.6, 31.9, 31.9, 29.5, 26.6, 22.6, 14.1. **HRMS (APCI)** Calc. for $C_{20}H_{29}$ [M+H$^+$] 269.2264, found 269.2234. **Optical rotation**: $[\alpha]_D^{21}$ + 6.6 (c=0.57, $CHCl_3$).

Enantiomeric ratio was determined on derivatization product **28** (see section 10).

(*R*)-1-Cyclohexyl-3-(1-phenylallyl)bicyclo[1.1.1]pentane (22)

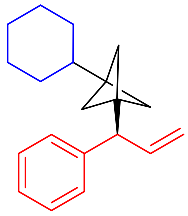

Synthesized from **1**, **40** and **3** according to general procedure B. Yellow oil obtained in 43% yield with >99:1 rr and 93:7 er after column chromatography (Pentane 100%). **^{1}H NMR** (500 MHz, $CDCl_3$) δ 7.31 – 7.26 (m, 2H), 7.21 – 7.17 (m, 1H), 7.15 – 7.11 (m, 2H), 6.14 – 6.00 (ddd, J = 17.1, 10.3, 8.3 Hz 1H), 5.07 (d, J = 10.3 Hz, 1H), 5.03 (d, J = 17.1 Hz, 1H), 3.37 (d, J = 8.3 Hz, 1H), 1.38 – 1.32 (m, 6H), 1.26 – 1.14 (m, 6H), 1.11 – 1.02 (m, 2H), 0.85 – 0.75 (m, 3H).**^{13}C NMR** (126 MHz, $CDCl_3$) δ 141.4, 137.8, 127.1 (2C), 127.0 (2C), 124.9, 114.3, 48.1, 45.7 (3C), 45.7, 43.2, 40.4, 37.2, 28.3 (2C), 25.1 (2C). **HRMS (APCI)** Calc. for $C_{20}H_{27}$ [M+H$^+$] 267.2107, found 267.2104. **Optical rotation**: $[\alpha]_D^{21}$+ 1.2 (c=0.69, $CHCl_3$).

Enantiomeric ratio was determined on derivatization product **29** (see section 10).

(*R*)-1-Cyclohexyl-3-(4-methylpent-1-en-3-yl)bicyclo[1.1.1]pentane (23)

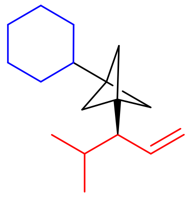

Synthesized from **1**, **40** and **50** according to general procedure B. Colorless oil obtained in 78% yield with >99:1 rr and 97:3 er after column chromatography (Pentane 100%). **^{1}H NMR** (500 MHz, $CDCl_3$) δ 5.49 (dt, J = 17.0, 10.0 Hz, 1H), 4.92 (dd, J = 10.0, 2.4 Hz, 1H), 4.82 (dd, J = 17.0, 2.4 Hz, 1H), 1.79 (dd, J = 9.9, 4.9 Hz, 1H), 1.68 – 1.59 (m, 3H), 1.57-1.52 (m, 3H), 1.38-1.30 (m, 6H), 1.21-1.16 (m, 1H), 1.16-1.04 (m, 3H), 1.01 (m, 2H), 0.81 (d, J = 6.7 Hz, 3H), 0.74 (d, J = 6.8 Hz, 3H). **^{13}C NMR** (126 MHz, $CDCl_3$) δ 137.0, 114.5, 47.0, 46.6 (3C), 42.8, 39.7, 37.2, 28.7, 28.3 (2C), 25.3, 25.1 (2C), 21.1, 18.1. **HRMS (APCI)** Calc. for $C_{17}H_{28}$ [M$^+$] 232.2186, found 232.2180. **Optical rotation**: $[\alpha]_D^{21}$ -21.0 (c=0.20, $CHCl_3$).

Enantiomeric ratio was determined on derivatization product **30** (see section 10).

(*R*)-1-(Tert-butyl)-3-(1-phenylallyl)bicyclo[1.1.1]pentane (24)

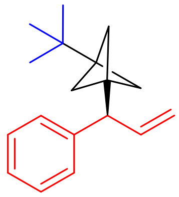

Synthesized from **1**, **41** and **3** according to general procedure B. White solid obtained in 85% yield with >99:1 rr and 91.5:8.5 er after column chromatography (Pentane 100%). **^{1}H NMR** (500 MHz, $CDCl_3$) δ 7.38 – 7.30 (m, 2H), 7.26 – 7.22 (m, 1H), 7.19 – 7.13 (m, 2H), 6.21 – 6.01 (m, 1H), 5.16 – 5.01 (m, 2H), 3.42 (d, J = 8.4 Hz, 1H), 1.46 – 1.35 (m, 6H), 0.82 (s, 9H).**^{13}C NMR** (126 MHz, $CDCl_3$) δ 142.6, 138.9, 128.3 (2C), 128.1 (2C), 126.1, 115.5, 48.0, 45.5 (3C), 45.4, 40.0, 29.7, 26.1 (3C).**HRMS (APCI)** Calc. for $C_{18}H_{25}$ [M+H$^+$] 241.1951, found 241.1952. **Optical rotation**: $[\alpha]_D^{21}$+ 4,7 (c=0.48, $CHCl_3$).

Enantiomeric ratio was determined on derivatization product **31** (see section 10).

10. Derivatization of the chiral bicyclo[1.1.1]pentanes by olefin metathesis (General procedure C)

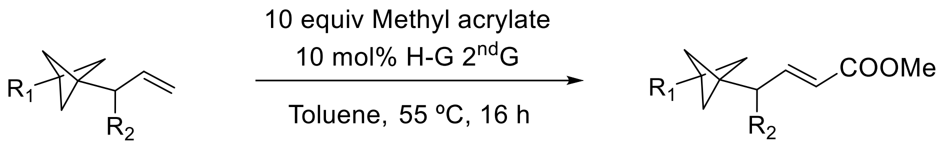

A flame-dried Schlenk tube charged with the 2nd generation Hoveyda-Grubbs catalyst (0.01 mmol, 0.1 equiv) was put under vacuum and backfilled with argon for three times. Afterwards, a solution of the corresponding bicyclo[1.1.1]pentane (1 equiv) and methyl acrylate (10 equiv) in dry toluene(0.1 M) was added the reaction was heated for 16 h in an oil bath. The volatiles were removed, and the resulting residue was purified by silica gel column chromatography with a mixture of Hexanes:AcOEt as eluent.

Methyl (*R,E*)-5-methyl-4-(3-phenylbicyclo[1.1.1]pentan-1-yl)hex-2-enoate (25)

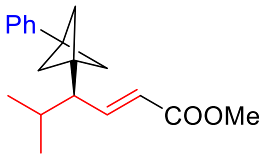

Synthesized from **10** according to general procedure C. Yellow oil obtained in 43 % yield with 98:2 er after column chromatography (Hex:AcOEt 98:2) **^{1}H NMR** (500 MHz, $CDCl_3$) δ 7.33 – 7.25 (m, 2H), 7.23-7.17 (m, 3H), 6.82 (dd, *J* = 15.5, 10.5 Hz, 1H), 5.81 (dd, *J* = 15.5, 0.8 Hz, 1H), 3.75 (s, 3H), 2.21 – 2.14 (m, 1H), 1.99 – 1.92 (m, 6H), 1.91 – 1.85 (m, 1H), 0.96 (d, *J* = 6.9 Hz, 3H), 0.90 (d, *J* = 6.7 Hz, 3H). **^{13}C NMR** (126 MHz, $CDCl_3$) δ 167.2, 149.2, 140.5, 128.5 (2C), 128.2 (2C), 126.7, 121.7, 51.6, 50.9, 48.7 (3C), 41.9, 41.2, 24.9, 10.7 (2C). **HRMS (APCI)** Calc. for $C_{19}H_{25}O_2$ [M+H$^+$] 285.1849, found 285.1856. **Optical rotation**: $[\alpha]_D^{18}$– 23.26 (c=0.90, $CHCl_3$).

Enantiomeric purity was determined by chiral uHPLC [Chiralpak IB N-3, T_{oven}: 40 °C, Flow: 0.5 mL/min; Hexane:*i*PrOH 99:1, λ =217nm, major enantiomer t_R = 2.97 min, minor enantiomer t_R = 2.82 min].

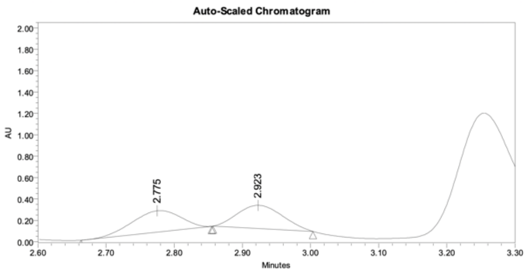

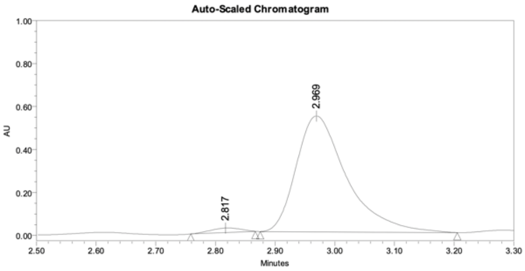

Peak Results

	RT	Area	% Area	Height
1	2.775	981896	49.63	199535
2	2.923	996491	50.37	216587

Peak Results

	RT	Area	% Area	Height
1	2.817	74190	2.29	20837
2	2.969	3168469	97.71	539202

Note: the other peak in racemic chromatogram corresponds to the linear regioisomer.

Methyl (*R,E*)-4-(3-phenylbicyclo[1.1.1]pentan-1-yl)pent-2-enoate (26)

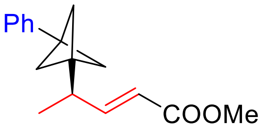

Synthesized from **13** according to general procedure C. Yellow oil obtained in 76 % yield with 81:19 er after column chromatography (Hex:AcOEt 98:2) ^{1}**H NMR** (500 MHz, $CDCl_3$) δ 7.32 – 7.27 (m, 2H), 7.22 – 7.20 (m, 2H), 7.20 – 7.19 (m, 1H), 6.90 (dd, *J* = 15.7, 7.8 Hz, 1H), 5.83 (dd, *J* = 15.7, 1.2 Hz, 1H), 3.75 (s, 3H), 2.56-2.49 (m, 1H), 1.90 (s, 6H), 1.07 (d, *J* = 6.8 Hz, 3H).^{13}**C NMR** (126 MHz, $CDCl_3$) δ 167.3, 151.6, 141.2, 128.3 (2C), 126.5, 126.2 (2C), 120.3, 51.6, 50.5 (3C), 41.9, 41.3, 37.7, 15.7. **HRMS (APCI)** Calc. for $C_{17}H_{21}O_2$ [M+H$^+$] 257.1536, found 257.1534. **Optical rotation**: $[\alpha]_D^{21}$+ 2.0 (c=0.49, $CHCl_3$).

Enantiomeric purity was determined by chiral uHPLC [Chiralpak IE-3, T_{oven}: 40 °C, Flow: 0.5 mL/min; Hexane:*i*PrOH 98:2, λ =222.5 nm, major enantiomer t_R = 3.45 min, minor enantiomer t_R = 2.94 min]

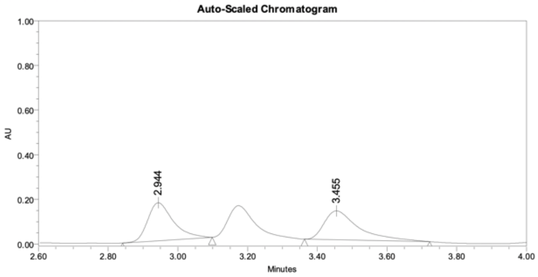

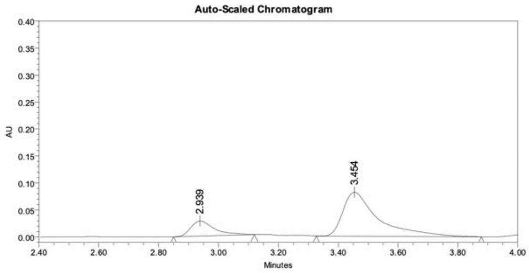

Peak Results

	RT	Area	% Area	Height
1	2.944	871675	49.41	170428
2	3.455	892548	50.59	129708

Peak Results

	RT	Area	% Area	Height
1	2.939	154343	18.90	27931
2	3.454	662277	81.10	81294

Note: the other peak in racemic chromatogram corresponds to the linear regioisomer.

Methyl (*R,E*)-4-(3-ethylbicyclo[1.1.1]pentan-1-yl)-4-phenylbut-2-enoate (27)

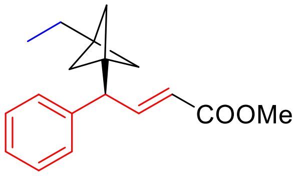

Synthesized from **20** according to general procedure C. Yellow oil obtained in 50 % yield with >99:1 rr and 87:13 er after column chromatography (Hex:AcOEt 98:2) ^{1}**H NMR** (500 MHz, $CDCl_3$) δ 7.34 – 7.26 (m, 2H), 7.24 – 7.17 (m, 2H), 7.16 – 7.10 (m, 2H), 5.81 (dd, *J* = 15.6, 1.3 Hz, 1H), 3.72 (s, 3H), 3.53 (d, *J* = 8.4 Hz, 1H), 1.52 – 1.30 (m, 8H), 0.78 (t, *J* = 7.5 Hz, 3H).^{13}**C NMR** (126 MHz, CDCl3) δ 167.2, 149.2, 140.5, 128.5 (2C), 128.2 (2C), 126.7, 121.7, 51.6, 50.9, 48.7 (3C), 41.9, 41.6, 24.9, 10.7. **HRMS (APCI)** Calc. for $C_{18}H_{23}O_2$ [M+H$^+$] 271.1693, found 271.1692. **Optical rotation**: $[\alpha]_D^{21}$+ 23.32 (c=0.55, $CHCl_3$).

Enantiomeric purity was determined by chiral uHPLC [Chiralpak IB N-3, T_{oven}: 40 °C, Flow: 0.5 mL/min; Hexane:*i*PrOH 98:2, λ =236.6 nm, major enantiomer t_R = 7.85 min, minor enantiomer t_R = 7.55 min].

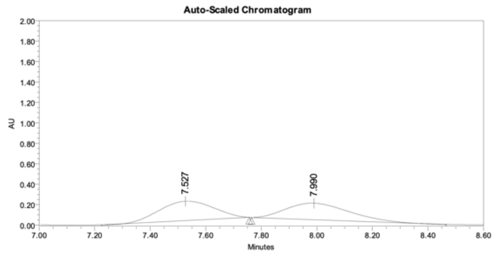

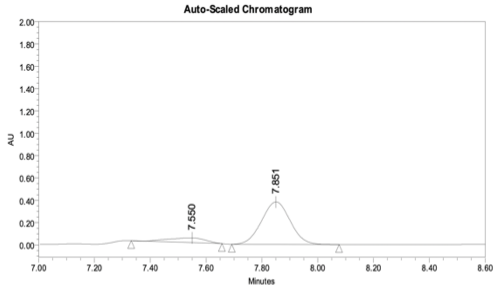

Peak Results

	RT	Area	% Area	Height
1	7.527	2597429	50.29	190261
2	7.990	2567247	49.71	161312

Peak Results

	RT	Area	% Area	Height
1	7.550	390136	12.60	42836
2	7.851	2706514	87.40	379803

Methyl (*R,E*)-4-(3-hexylbicyclo[1.1.1]pentan-1-yl)-4-phenylbut-2-enoate (28)

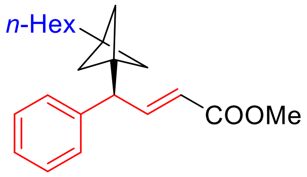

Synthesized from **21** according to general procedure C. Yellow oil obtained in 55 % yield with 91:9 er after column chromatography (Hex:AcOEt 98:2) **^{1}H NMR** (500 MHz, $CDCl_3$) δ 7.28 – 7.20 (m, 2H), 7.17 – 7.14 (m, 1H), 7.11 (dd, *J* = 15.6, 8.4 Hz, 1H), 7.07 – 7.04 (m, 2H), 5.74 (dd, *J* = 15.6, 1.2 Hz, 1H), 3.65 (s, 3H), 3.45 (d, *J* = 8.4 Hz, 1H), 1.44 – 1.34 (m, 6H), 1.22 – 1.08 (m, 10H), 0.79 (t, *J* = 7.1 Hz, 3H). **^{13}C NMR** (126 MHz, $CDCl_3$) δ 166.0, 148.0, 139.4, 127.4 (2C), 127.0 (2C), 125.5, 120.5, 50.4, 49.7, 48.2 (3C), 40.6, 39.9, 30.8, 30.8, 28.4, 25.6, 21.6, 13.1. **HRMS (APCI)** Calc. for $C_{22}H_{31}O_2$ [$M+H^+$] 327.2319, found 327.2319. **Optical rotation**: $[\alpha]_D^{21}$+ 9.3 (c=0.51, $CHCl_3$).

Enantiomeric purity was determined by chiral uHPLC analysis [Chiralpak IB N -3, T_{oven}: 40 °C, flow: 1.0 mL/min; Hexane:*i*PrOH 99:1, λ = 220.5 nm, major enantiomer t_R = 3.95 min, minor enantiomer t_R = 3.80 min].

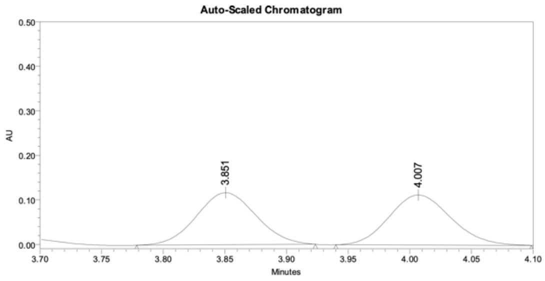

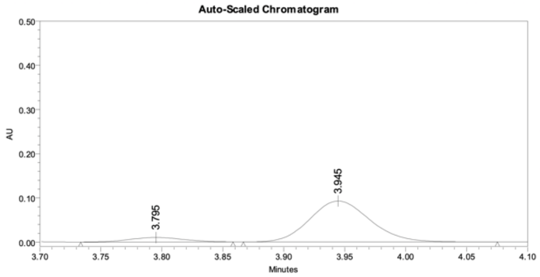

Peak Results

	RT	Area	% Area	Height
1	3.851	394040	50.17	116993
2	4.007	391424	49.83	112379

Peak Results

	RT	Area	% Area	Height
1	3.795	31853	9.07	10303
2	3.945	319348	90.93	92871

Methyl (*R,E*)-4-(3-cyclohexylbicyclo[1.1.1]pentan-1-yl)-4-phenylbut-2-enoate (29)

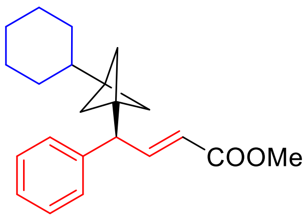

Synthesized from **22** according to general procedure C. Yellow oil obtained in 50 % yield with 93:7 er after column chromatography (Hex:AcOEt 98:2) ^{1}**H NMR** (500 MHz, $CDCl_3$) δ 7.32-7.24 (m, 2H), 7.25 – 7.17 (m, 2H), 7.15 – 7.10 (m, 2H), 5.81 (dd, *J* = 15.6, 1.2 Hz, 1H), 3.72 (s, 3H), 3.53 (d, *J* = 8.4 Hz, 1H), 2.00-1.60 (m, 4H), 1.52 (s, 1H), 1.46 – 1.36 (m, 6H), 1.26 – 0.88 (m, 4H), 0.85-0.69 (m, 2H).^{13}**C NMR** (126 MHz, $CDCl_3$) δ 167.4, 149.5, 140.8, 128.8 (2C), 128.4 (2C), 127.0, 122.0, 51.9, 51.2, 47.4 (3C), 45.0, 41.3, 38.5, 29.9 (2C), 26.7, 26.4 (2C). **HRMS (APCI)** Calc. for $C_{22}H_{29}O_2$ [$M+H^+$] 325.2162, found 325.2160. **Optical rotation**: $[\alpha]_D^{21}$+ 6.73 (c=0.46, $CHCl_3$).

Enantiomeric purity was determined by chiral uHPLC [Lux i-Amylose-3, T_{oven}: 40 °C, Flow: 1.0 mL/min; Hexane:*i*PrOH 98:2, λ =233.7 nm, major enantiomer t_R = 4.46 min, minor enantiomer t_R = 4.22 min].

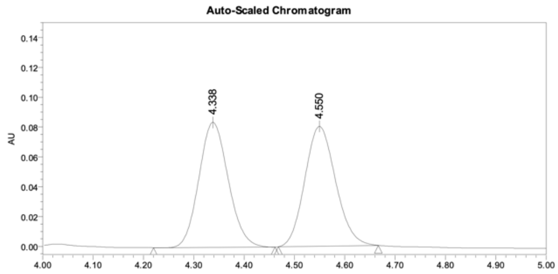

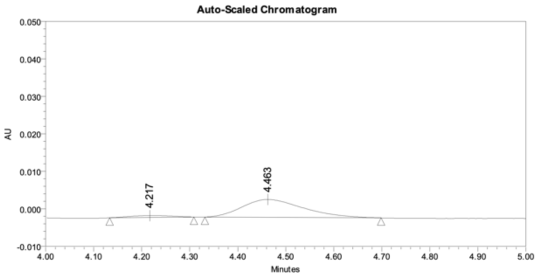

Peak Results

	RT	Area	% Area	Height
1	4.338	339826	49.88	83842
2	4.550	341501	50.12	80266

Peak Results

	RT	Area	% Area	Height
1	4.217	3053	6.71	510
2	4.463	42423	93.29	4752

Methyl (*R,E*)-4-(3-cyclohexylbicyclo[1.1.1]pentan-1-yl)-5-methylhex-2-enoate (30)

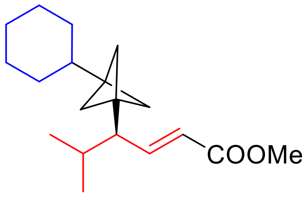

Synthesized from **23** according to general procedure C. Colorless oil obtained in 50% yield with >99:1 rr and 93:07 er after column chromatography (Hexane:AcOEt 100:0 to 99:1). ^{1}**H NMR** (500 MHz, $CDCl_3$) δ 6.76 (dd, *J* = 15.6, 10.5 Hz, 1H), 5.74 (dd, *J* = 15.5, 0.7 Hz, 1H), 3.73 (s, 3H), 2.05 (dd, *J* = 10.5, 5.3 Hz, 1H), 1.85 – 1.74 (m, 1H), 1.74 – 1.66 (m, 2H), 1.62 – 1.53 (m, 3H), 1.50 – 1.41 (m, 6H), 1.23 – 1.12 (m, 4H), 1.13 – 1.01 (m, 2H), 0.90 (d, *J* = 6.8 Hz, 3H), 0.84 (d, *J* = 6.8 Hz, 3H). ^{13}**C NMR** (126 MHz, $CDCl_3$) *δ* 165.9, 148.5, 120.9, 50.3, 49.7, 46.9 (3C), 43.3, 39.0, 37.1, 29.1, 28.3 (2C), 25.3 (2C), 25.1, 21.1, 18.4. **HRMS (APCI)** Calc. for $C_{19}H_{31}O_2$ [$M+H^+$] 291.2319, found 291.2318. **Optical rotation**: $[\alpha]_D^{21}$-22.9 (c=0.50, $CHCl_3$).

Enantiomeric purity was determined by chiral uHPLC analysis [Lux i-Amyllose -3, T_{oven}: 40 °C, flow: 1.0 mL/min; Hexane:*i*PrOH 99:1, λ = 218.5 nm, major enantiomer t_R = 3.81 min, minor enantiomer t_R = 3.68 min].

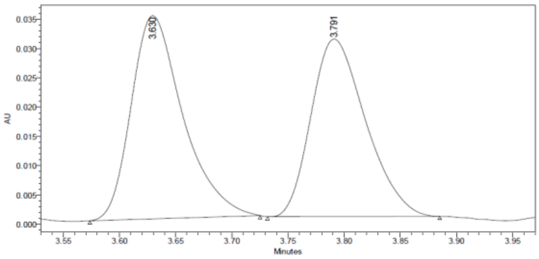

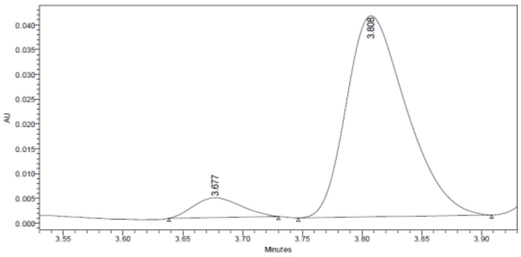

	Peak Type	RT	Area	% Area	Height
1	Unknown	3.630	111120	52.05	34660
2	Unknown	3.791	102354	47.95	30286

	Peak Type	RT	Area	% Area	Height
1	Unknown	3.677	10983	7.21	3985
2	Unknown	3.808	141396	92.79	40563

Methyl (*R,E*)-4-(3-(*tert*-butyl)bicyclo[1.1.1]pentan-1-yl)-4-phenylbut-2-enoate (31)

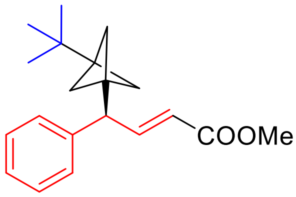

Synthesized from **24** according to general procedure C. Yellow oil obtained in 62 % yield with 91.5:8.5 er after column chromatography (Hex:AcOEt 98:2) ^{1}**H NMR** (500 MHz, $CDCl_3$) δ 7.35 – 7.28 (m, 2H), 7.25 – 7.22 (m, 1H), 7.19 (dd, *J* = 15.6, 8.5 Hz, 1H), 7.15 – 7.12 (m, 2H), 5.82 (dd, *J* = 15.6, 1.2 Hz, 1H), 3.73 (s, 3H), 3.53 (dd, *J* = 8.5, 1.2 Hz, 1H), 1.53 – 1.38 (m, 6H), 0.78 (d, *J* = 1.5 Hz, 9H). ^{13}**C NMR** (126 MHz, $CDCl_3$) δ 167.2, 149.4, 140.6, 128.6 (2C), 128.2 (2C), 126.8, 121.9, 51.6, 50.9, 49.0, 45.8 (3C), 39.5, 29.7, 26.1 (3C). **HRMS (APCI)** Calc. for $C_{20}H_{27}O_2$ [M+H$^+$] 299.2006, found 299.2004. **Optical rotation**: $[\alpha]_D^{21}$+ 3.4 (c=0.5, $CHCl_3$).

Enantiomeric purity was determined by chiral uHPLC [Chiralpak IB N-3, T_{oven}: 40 °C, Flow: 1.0 mL/min; Hexane:*i*PrOH 98:2, λ =238.3 nm, major enantiomer t_R = 1.20 min, minor enantiomer t_R = 2.14 min]

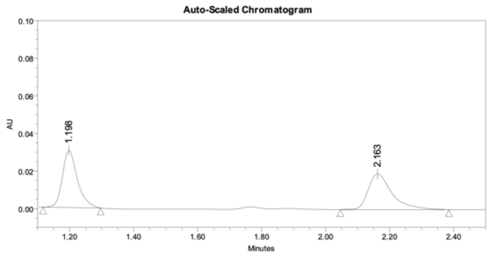

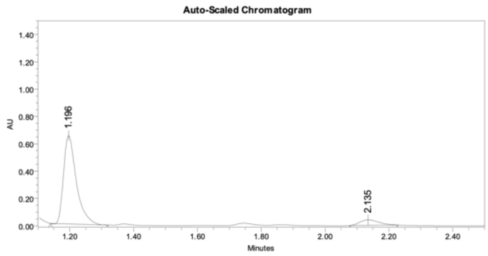

Peak Results

	RT	Area	% Area	Height
1	1.198	99101	50.55	30455
2	2.163	96957	49.45	18907

Peak Results

	RT	Area	% Area	Height
1	1.196	1801108	91.55	646734
2	2.135	166262	8.45	40402

11. Derivatization of chiral bicyclo[1.1.1]pentanes by hydroboration/oxidation (General procedure D)

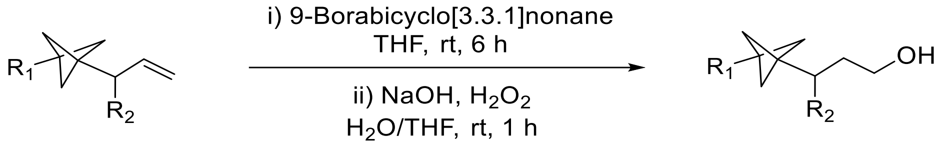

BCP-alcohols were obtained by a modified literature procedure. [1]

Allylic-BCP (1.0 equiv, 0.1 mmol) and THF (1 mL) were added to a flame-dried Schlenck. The Schlenck was cooled down to 0 ^{o}C and 9-borabicyclo[3.3.1]nonane (4.0 equiv, 0.4 mmol) was added under Ar atmosphere. The mixture was warmed up to rt and stirred for 6 hours at this temperature. Then, it was cooled down again to 0 ^{o}C and NaOH (2N, 4.0 equiv, 0.4 mmol) and H_2O_2 (10.0 equiv, 1 mmol) were added. The mixture was warmed up to rt and stirred for 1 h. The aqueous phase was extracted with ethyl acetate (10 ml x3), dried over Na_2SO_4 and filtered. The solvents were removed under vacuum and the crude was purified by a flash column chromatography using the indicated mixture of solvents.

(*R*)-3-(3-Phenylbicyclo[1.1.1]pentan-1-yl)hexan-1-ol (32)

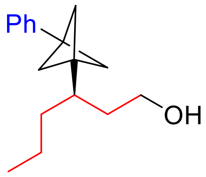

Synthesized from **14** according to general procedure D. Colorless oil obtained in 68% yield and 84:16 er after column chromatography (Hexane: ACOEt 100:0 to 90:10). **^{1}H NMR** (500 MHz, $CDCl_3$) δ 7.35 – 7.27 (m, 2H), 7.25 – 7.17 (m, 3H), 3.78 – 3.66 (m, 2H), 1.90 (s, 6H), 1.77 – 1.58 (m, 1H), 1.55 – 1.43 (m, 2H), 1.46 – 1.29 (m, 3H), 1.24 – 1.17 (m, 1H), 0.91 (t, *J* = 7.2 Hz, 3H). **^{13}C NMR**.δ 140.4, 127.1 (2C), 125.2, 125.0 (2C), 60.7, 50.0 (3C), 41.6, 40.2, 33.9, 33.8, 33.1, 19.8, 13.5. **HRMS (APCI)** Calc. for $C_{17}H_{25}O$ [M+H$^+$] 245.1900, found 245.1890. **Optical rotation**: $[\alpha]_D^{20}$- 22.8 (c=0.41, $CHCl_3$).

Enantiomeric purity was determined by chiral uHPLC analysis [Chiralpak IF-3, T_{oven}: 40 °C, flow: 1.0 mL/min; Hexane:*i*PrOH 99:1, λ = 216.5 nm, major enantiomer t_R = 4.42 min, minor enantiomer t_R = 4.84 min].

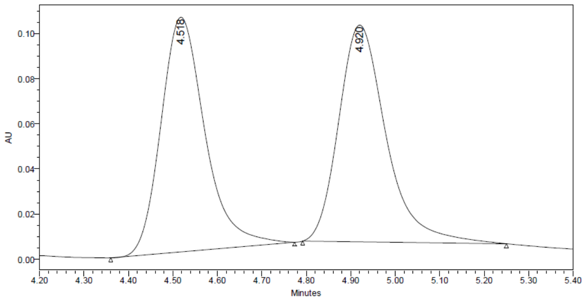

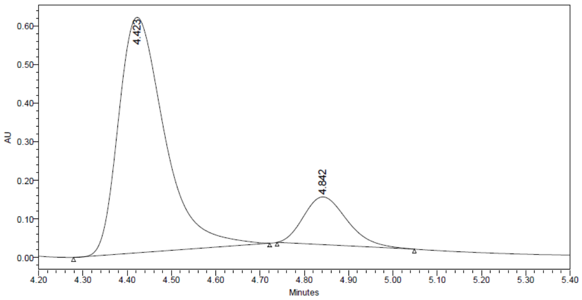

	Peak Type	RT	Area	% Area	Height
1	Unknown	4.518	716850	50.11	103992
2	Unknown	4.920	713591	49.89	96088

	Peak Type	RT	Area	% Area	Height
1	Unknown	4.423	4421332	83.93	610595
2	Unknown	4.842	846597	16.07	123728

(*S*)-3-(3-(4-Fluorophenyl)bicyclo[1.1.1]pentan-1-yl)-4-methylpentan-1-ol (33)

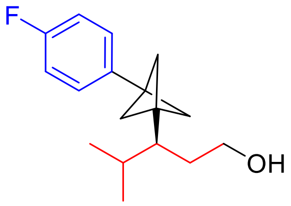

Synthesized from **18** according to general procedure D. Colorless oil obtained in 89% yield and 92.5:7.5 er after column chromatography (Hexane:AcOEt 100:0 to 90:10). **^{1}H NMR** (500 MHz, $CDCl_3$) δ 7.19 – 7.10 (m, 2H), 7.03 – 6.90 (m, 2H), 3.79 – 3.70 (m, 1H), 3.69 – 3.59 (m, 1H), 2.01 – 1.90 (m, 6H), 1.84 (m, 1H), 1.70 – 1.49 (m, 2H), 1.52 – 1.46 (m, 1H), 1.26 (b*s*, 1H), 0.94 (d, *J* = 7.0 Hz, 3H), 0.89 (d, *J* = 6.9 Hz, 3H). **^{13}C NMR** (126 MHz, $CDCl_3$) δ 161.6 (d, *J* = 244.2 Hz), 137.1 (d, *J* = 3.2 Hz), 127.5 (d, *J* = 7.8 Hz, 2C), 114.84 (d, *J* = 21.3 Hz, 2C), 62.6, 52.5 (3C), 42.1, 41.1, 40.6, 31.1, 29.7, 20.9, 18.6. **^{19}F NMR {1H}** (282 MHz, $CDCl_3$) δ -116.9. **HRMS (APCI)** Calc. for $C_{17}H_{24}FO$ [M+H$^+$] 263.1806, found 263, 1796. **Optical rotation**: $[\alpha]_D^{20}$ - 2.50 (c= 1.17, $CHCl_3$).

Enantiomeric purity was determined by chiral uHPLC analysis [Lux i-Amyllose-3, T_{oven}: 40 °C, flow: 1.0 mL/min; Hexane:*i*PrOH 99:1, λ = 220 nm, major enantiomer t_R = 16.61 min, minor enantiomer t_R = 17.52 min].

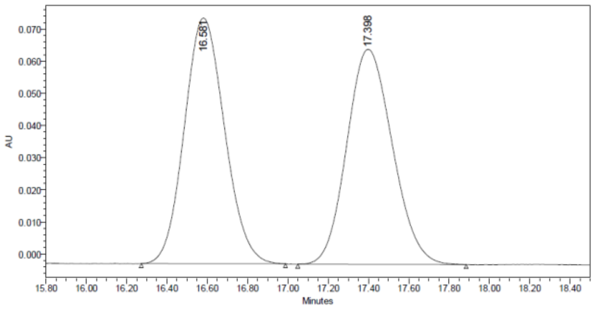

	Peak Type	RT	Area	% Area	Height
1	Unknown	16.581	1063030	50.24	76361
2	Unknown	17.398	1053071	49.76	66899

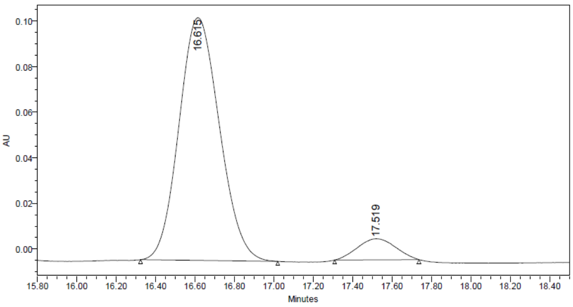

	Peak Type	RT	Area	% Area	Height
1	Unknown	16.615	1520375	92.45	106520
2	Unknown	17.519	124225	7.55	9240

12. Unsuccessful examples

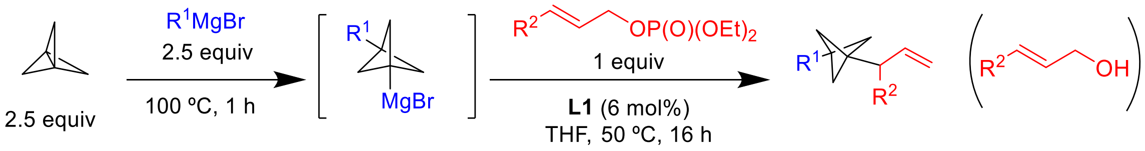

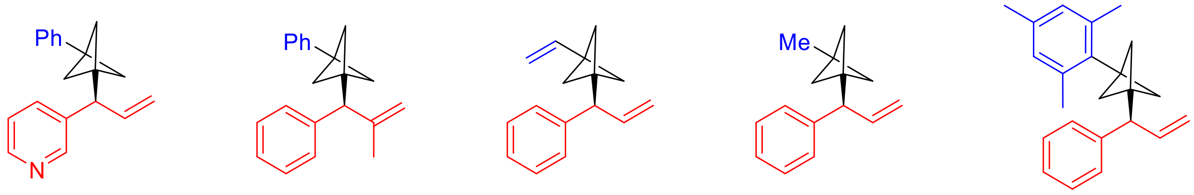

60%
r.r: >99:1
e.r: 72:28

<5%

7%

<5%

<5%

13. NMR spectra

1H-NMR (CDCl3, 500 MHz)

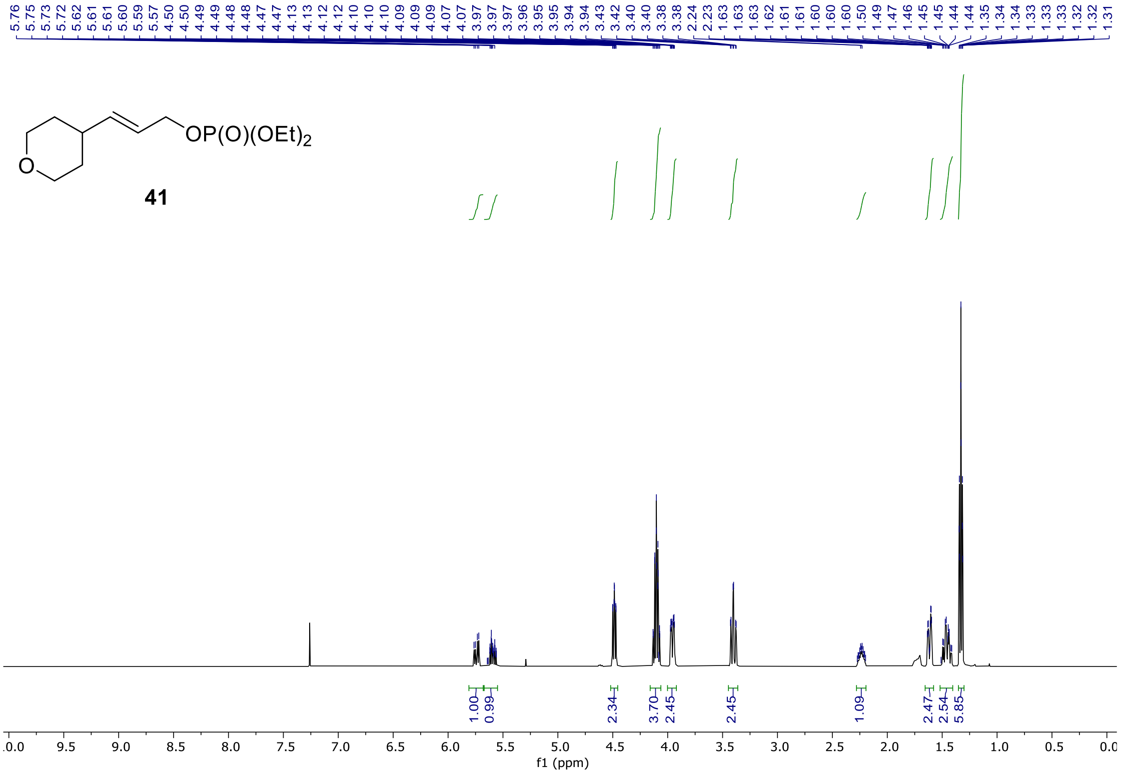

13C-NMR (CDCl3, 126 MHz)

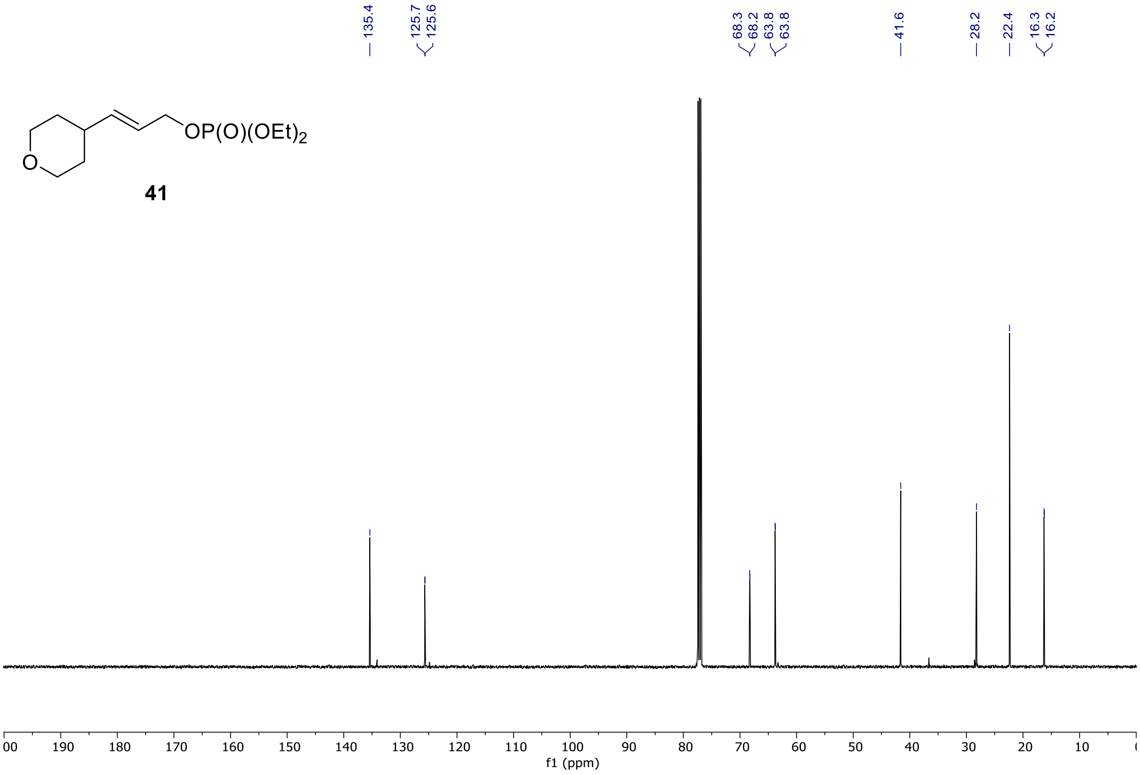

31P-NMR (CDCl3, 202 MHz)

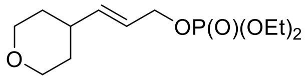

41

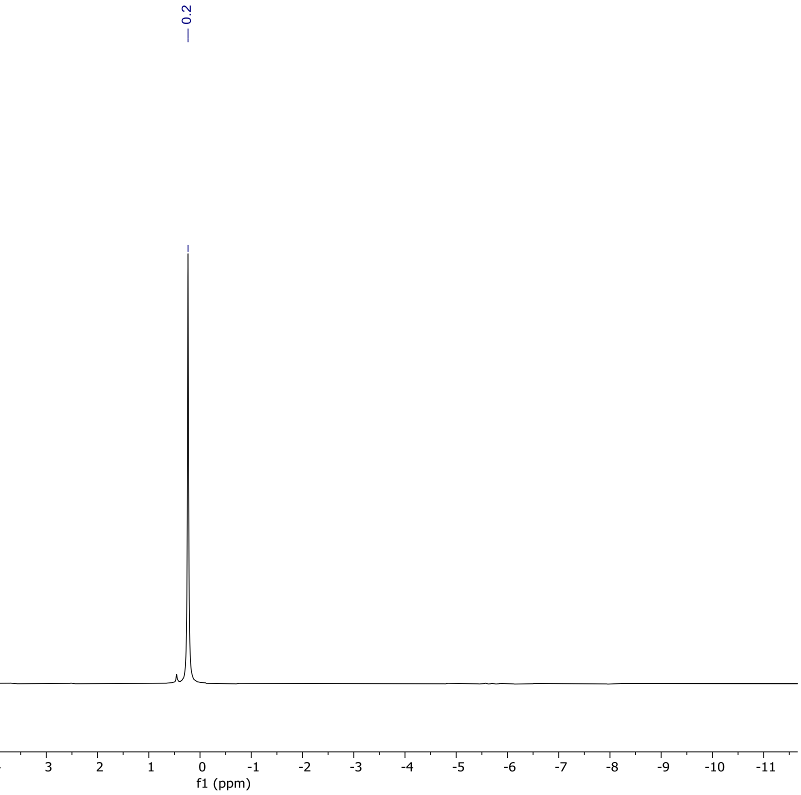

1H-NMR (CDCl3, 500 MHz)

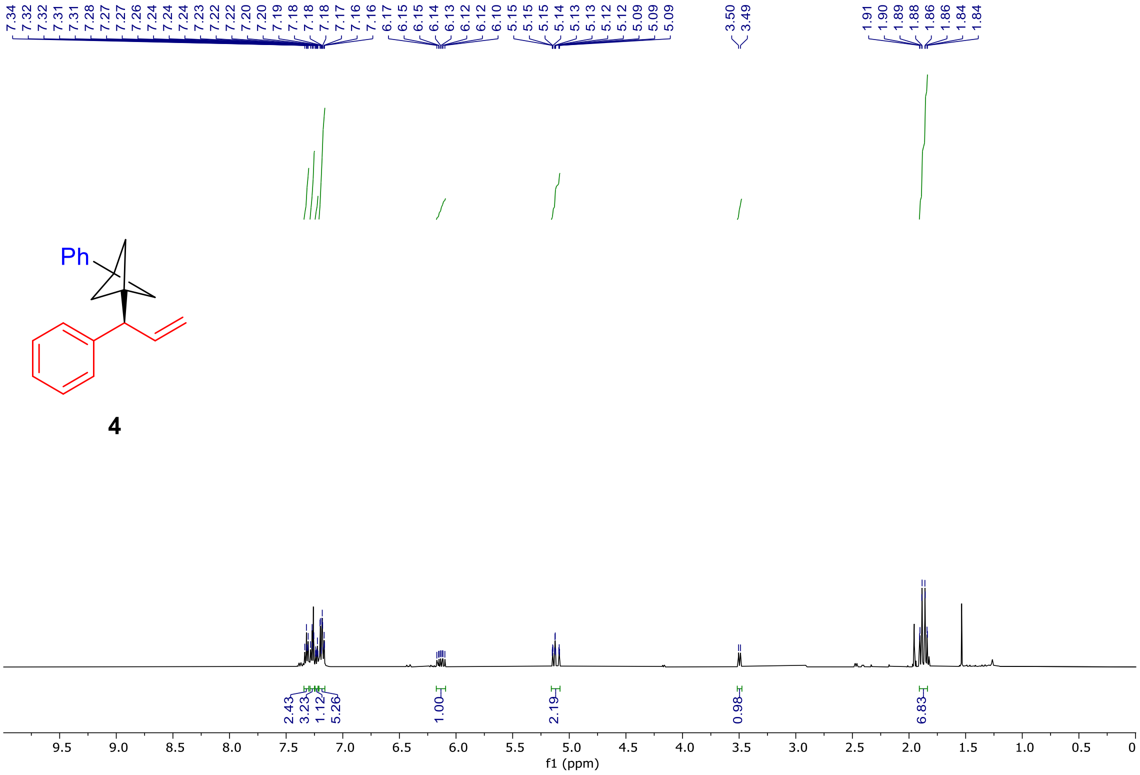

13-NMR (CDCl3, 126 MHz)

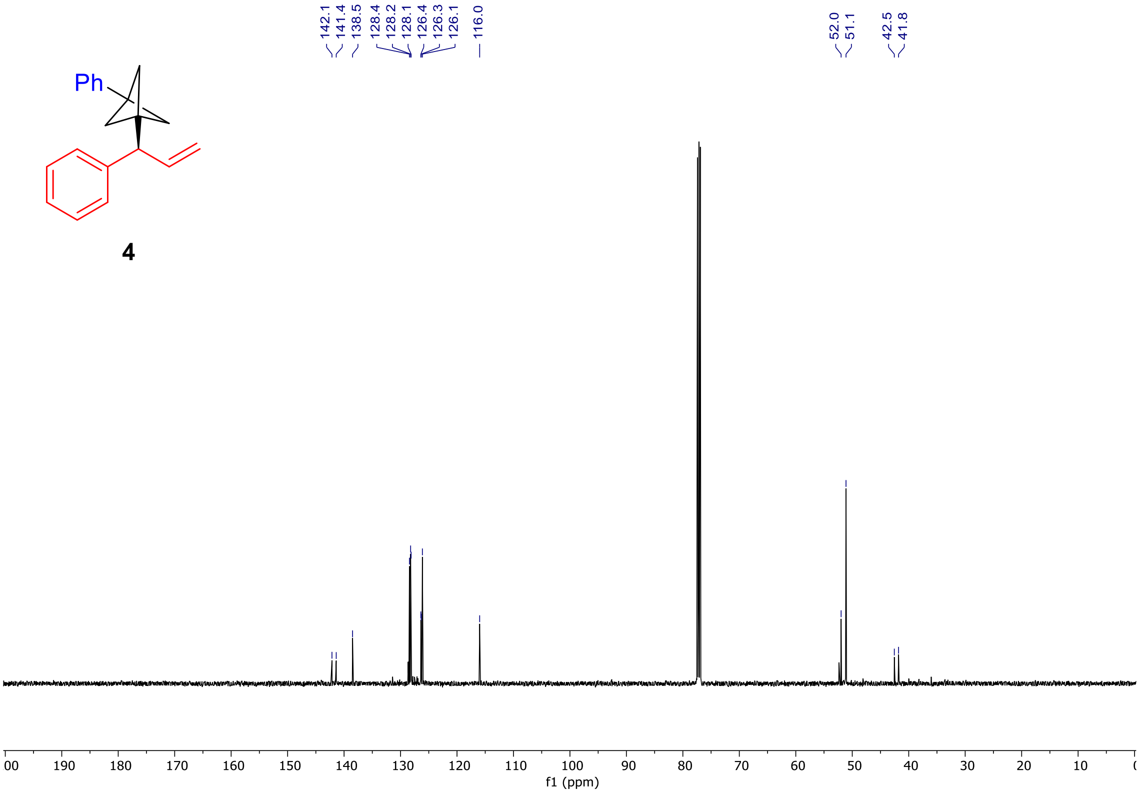

1H-NMR (CDCl3, 500 MHz)

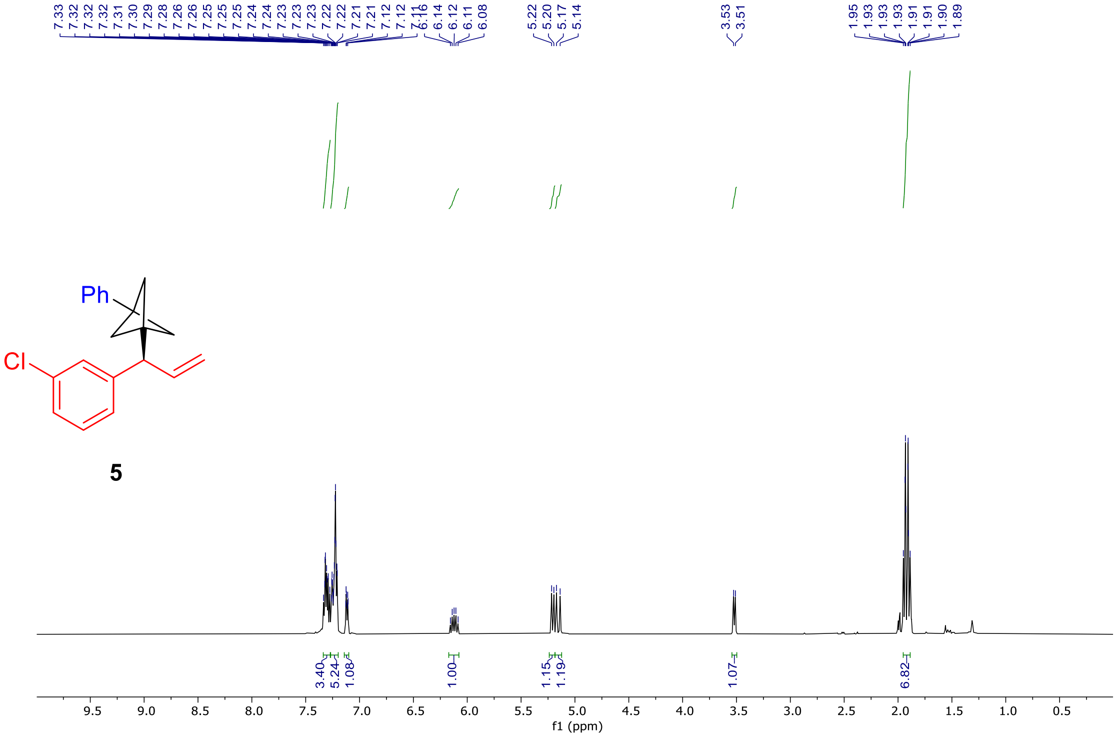

13C-NMR (CDCl3, 126 MHz)

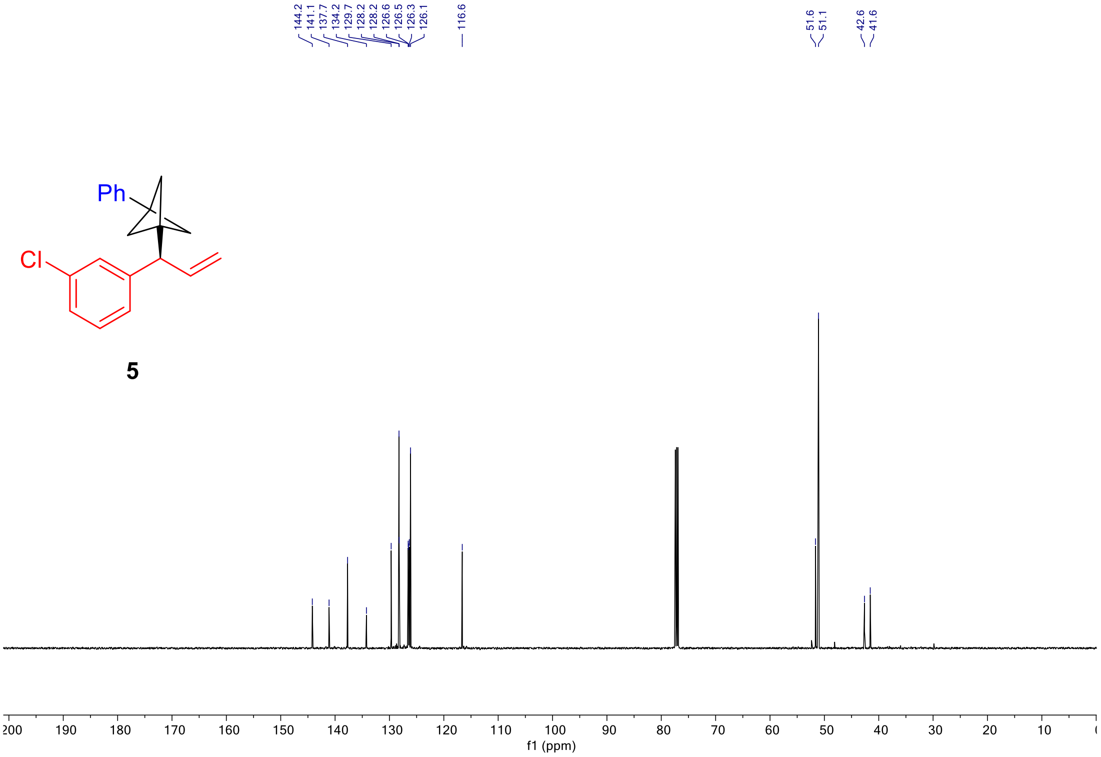

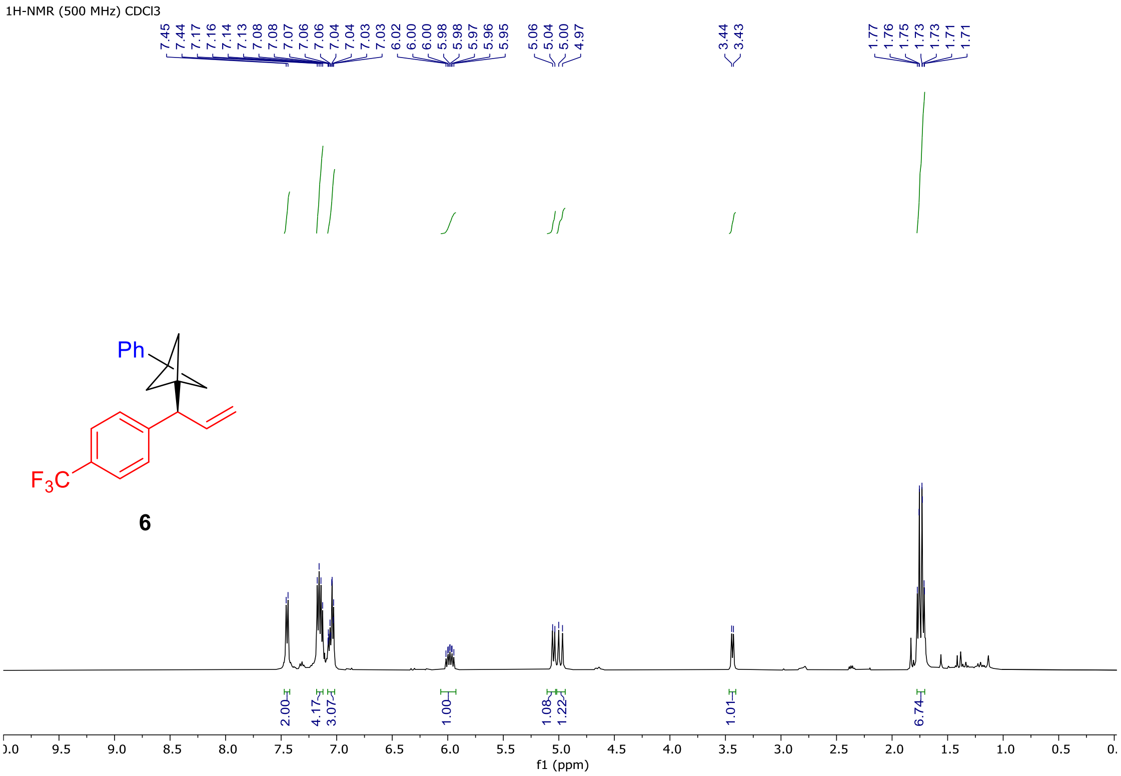

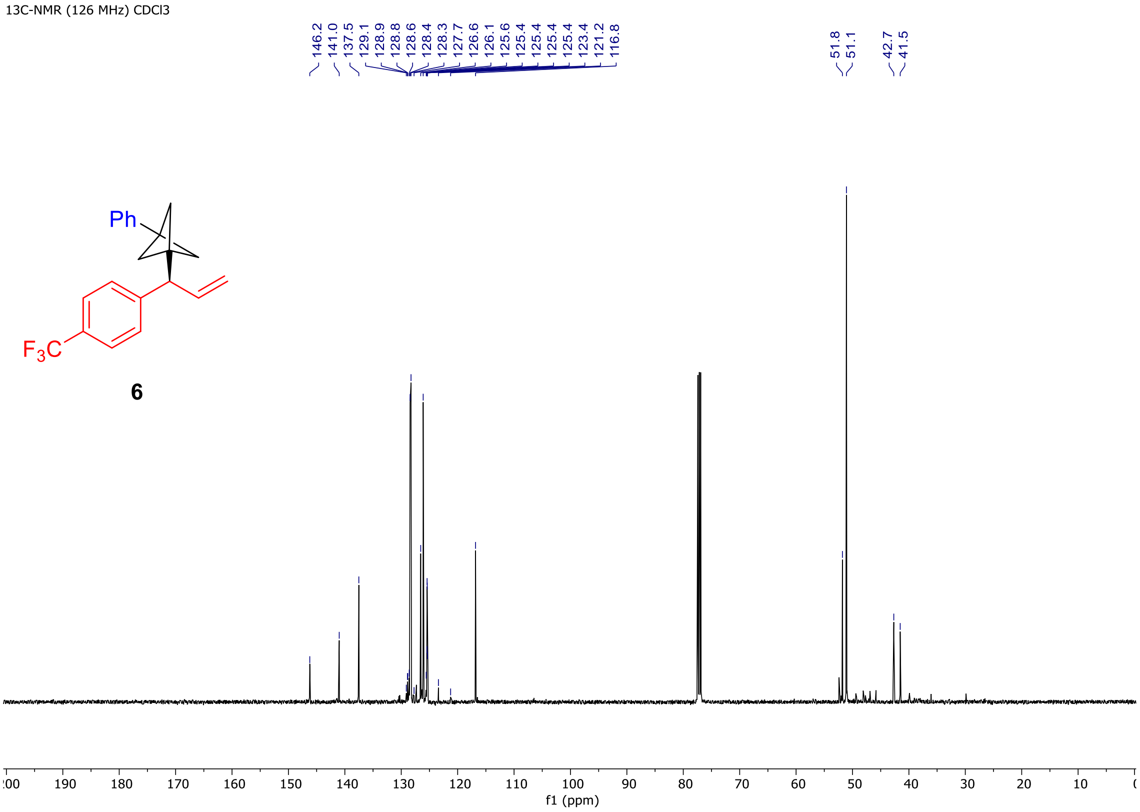

Note: extra peaks are related to the linear isomer (<5%)

19F-NMR (CDCl3, 202 MHz)

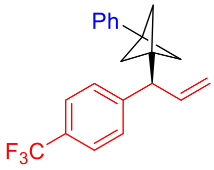

6

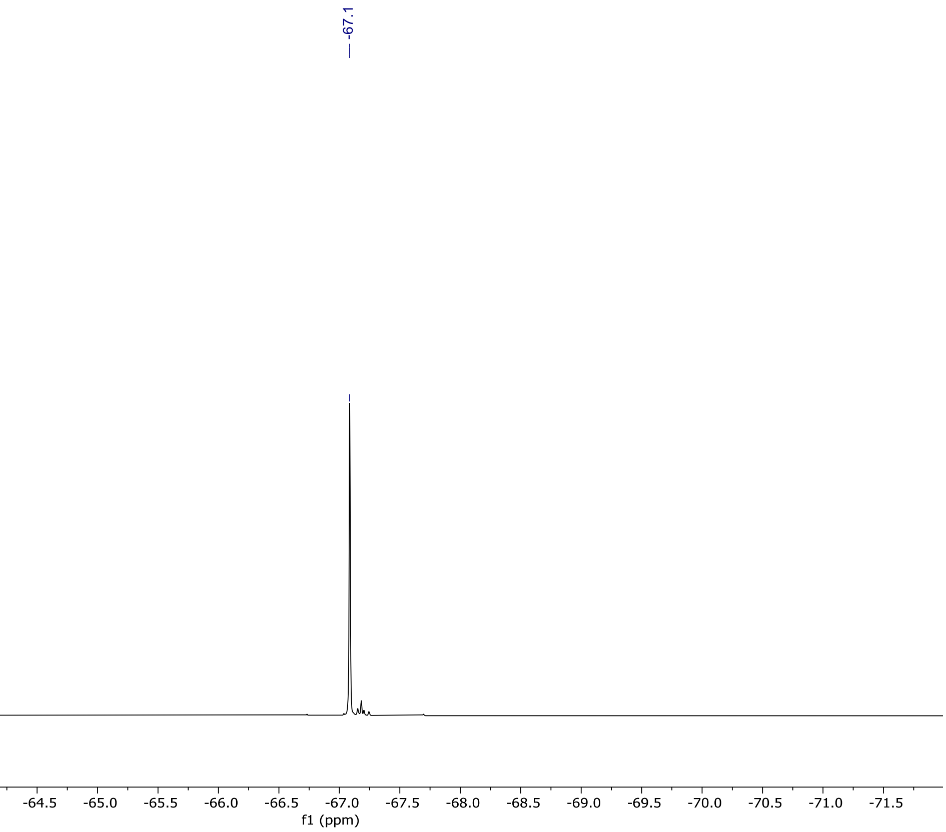

1H-NMR (CDCl3, 500 MHz)

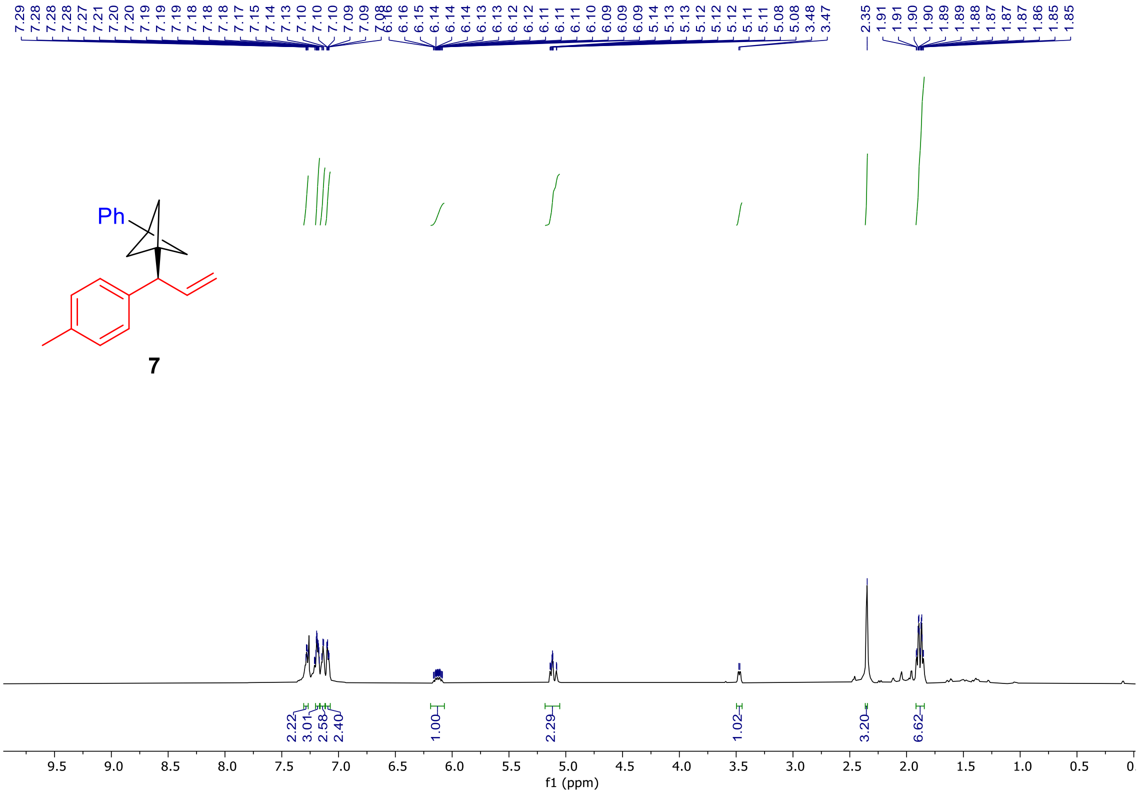

13C-NMR (CDCl3, 125 MHz)

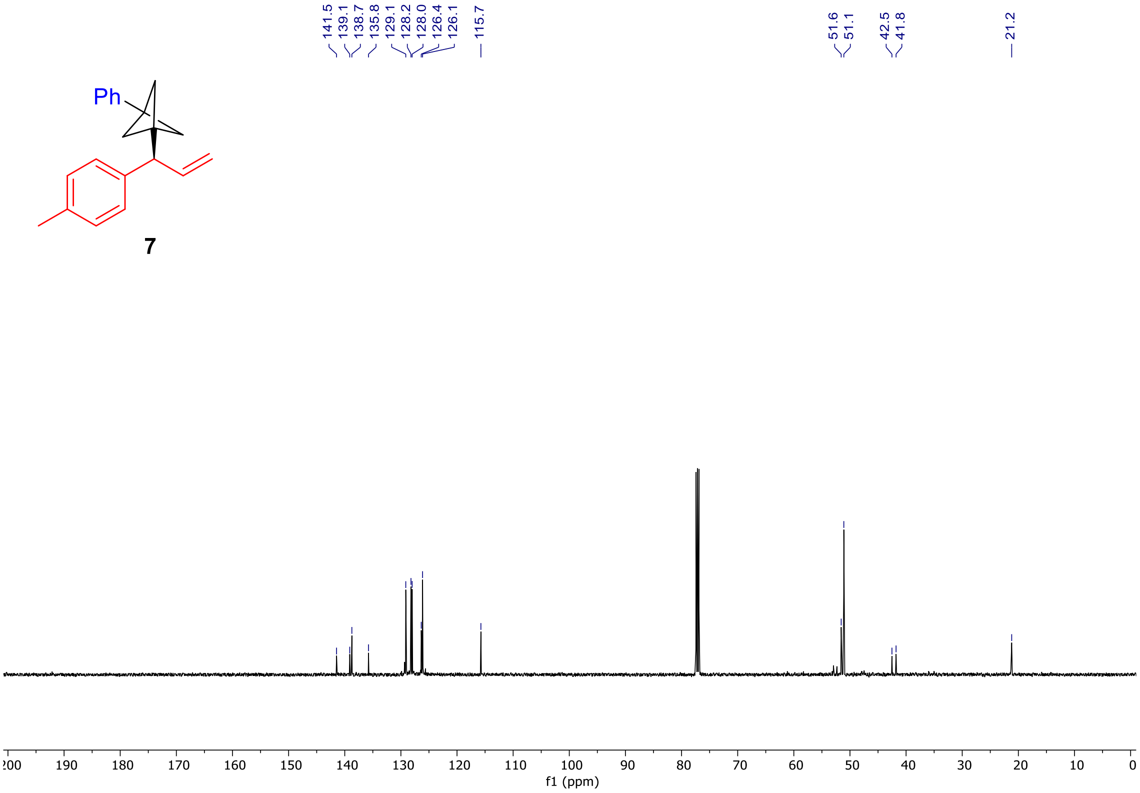

1H-NMR (CDCl3, 500 MHz)

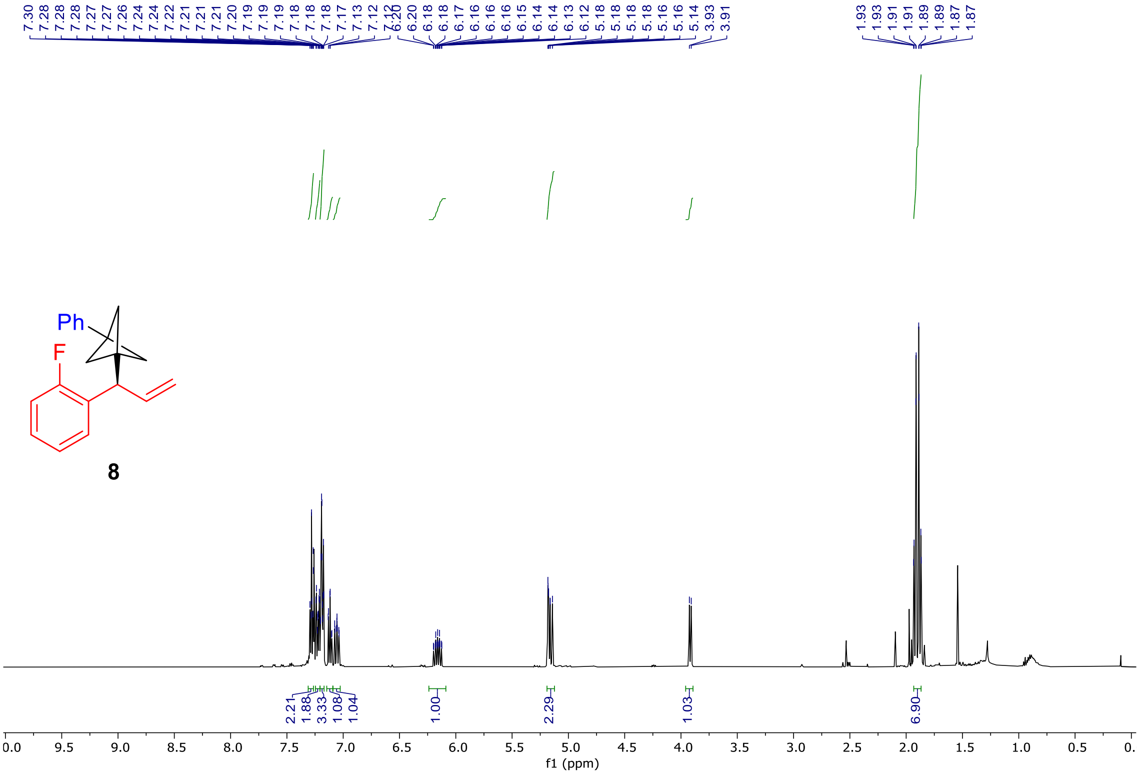

13C-NMR (CDCl3, 126 MHz)

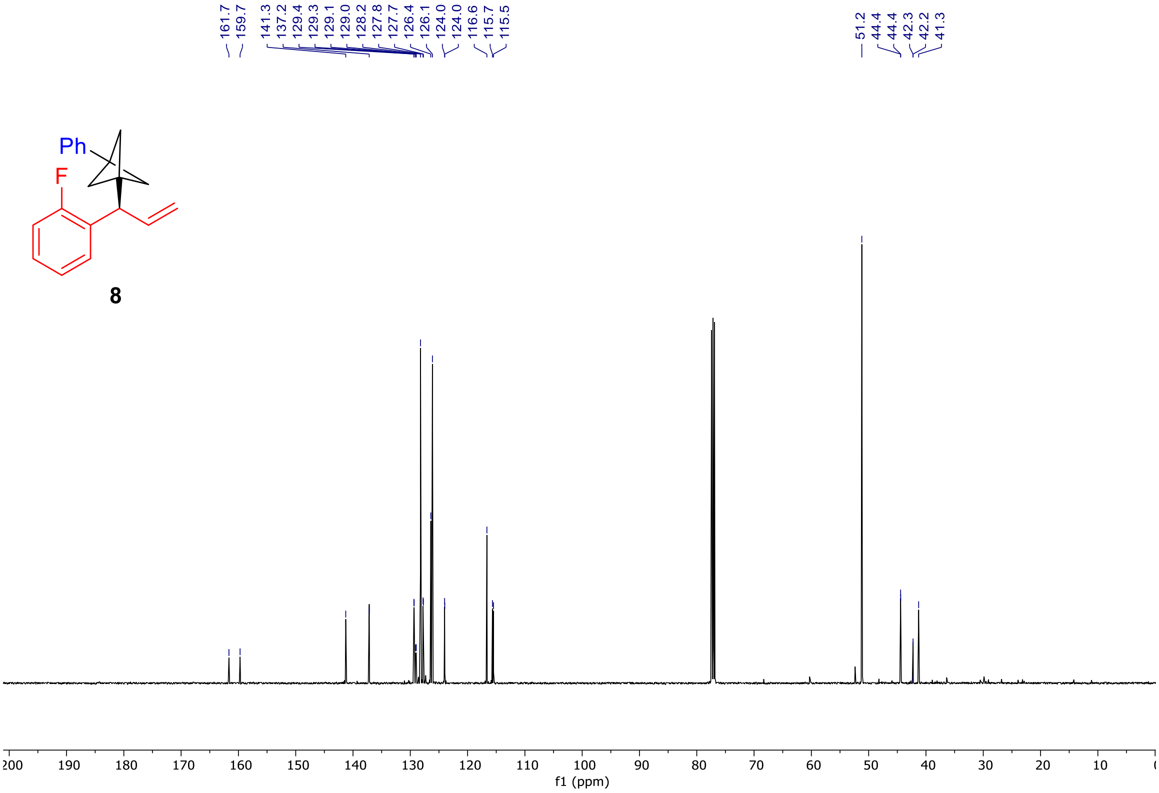

19F-NMR (CDCl3, 202 MHz)

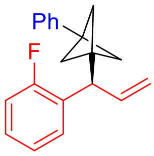

8

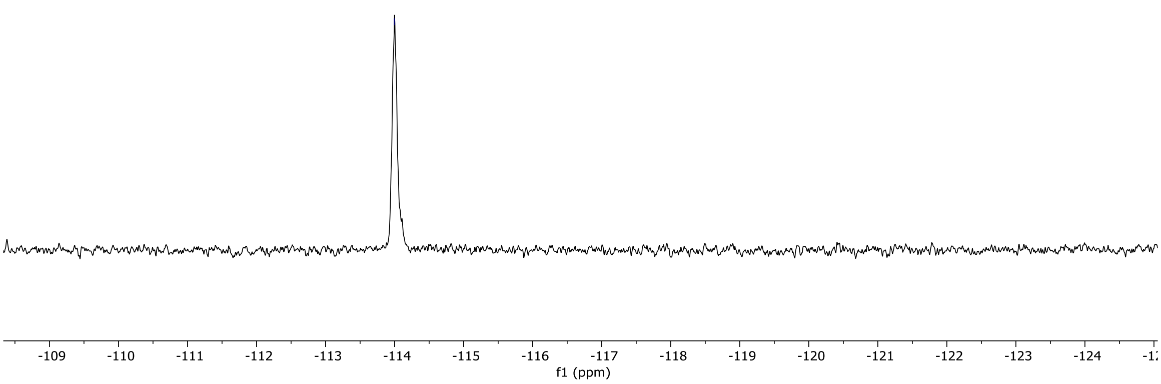

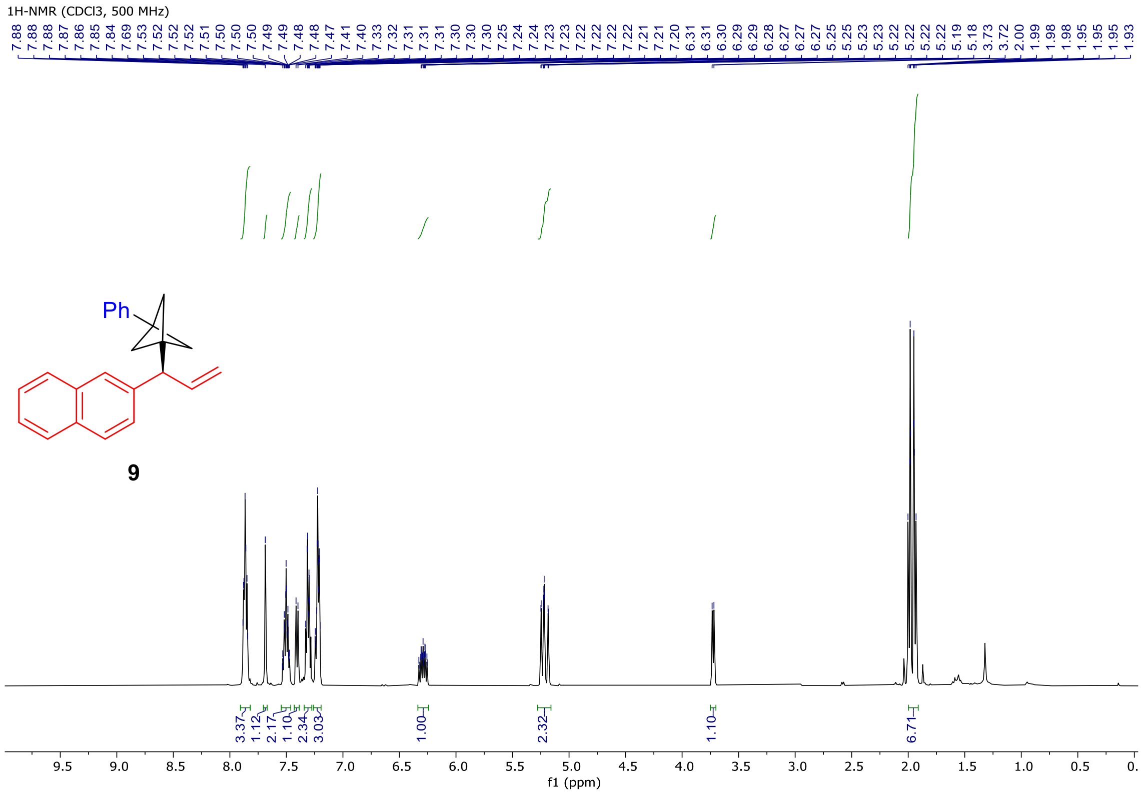
1H-NMR (CDCl3, 500 MHz)
Ph
9
f1 (ppm)

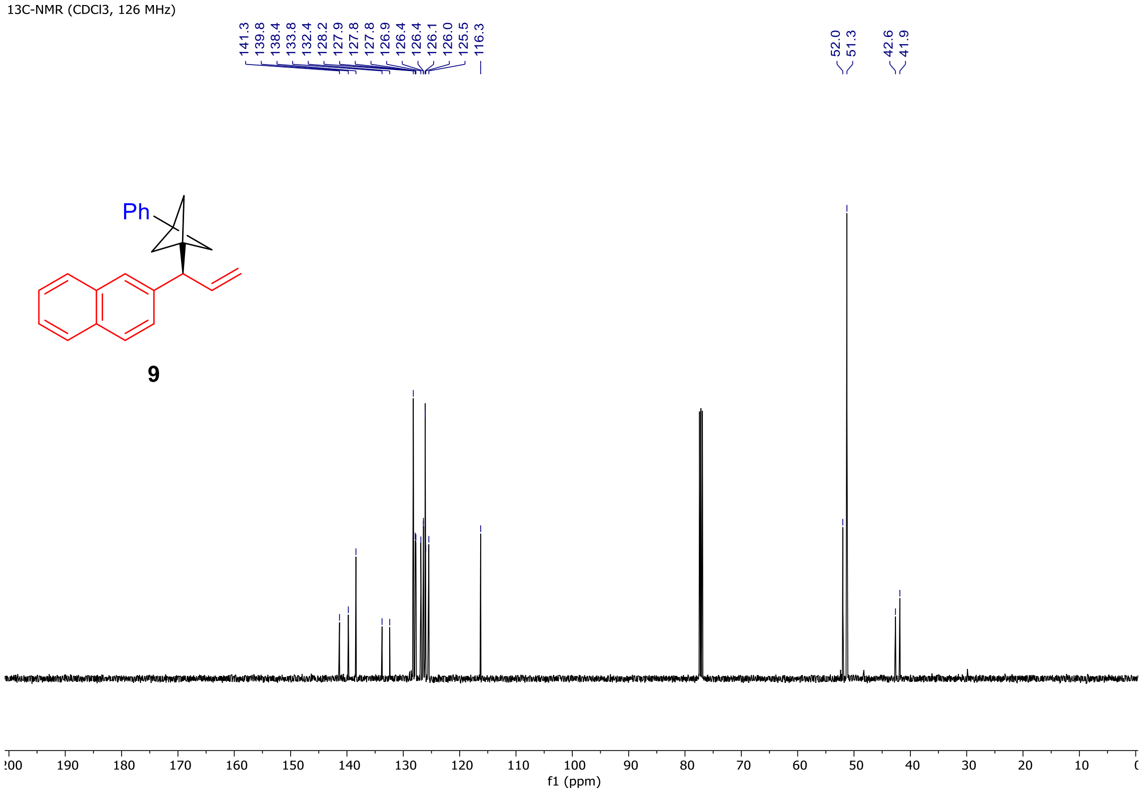
13C-NMR (CDCl3, 126 MHz)
141.3
139.8
138.4
133.8
132.4
128.2
127.9
127.8
127.8
126.9
126.4
126.4
126.1
126.0
125.5
116.3
52.0
51.3
42.6
41.9
Ph
9
f1 (ppm)

1H-NMR (CDCl3, 500 MHz)

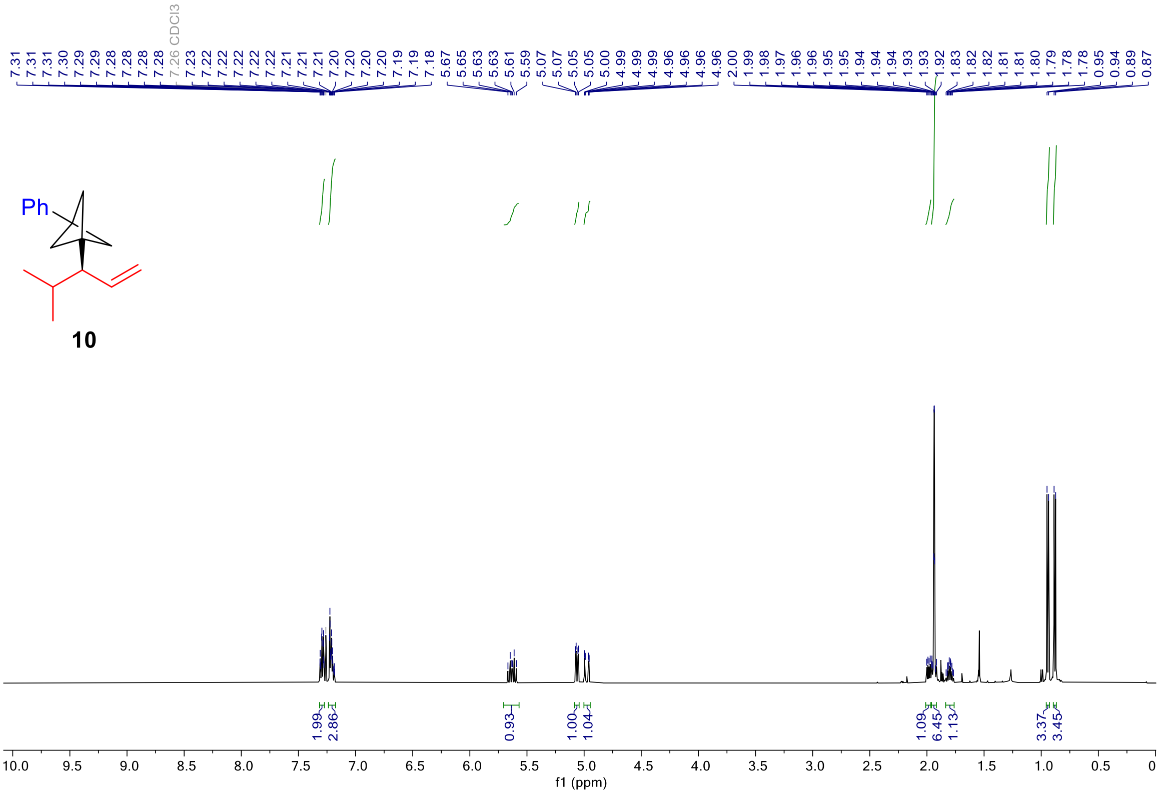

13C-NMR (CDCl3, 126 MHz)

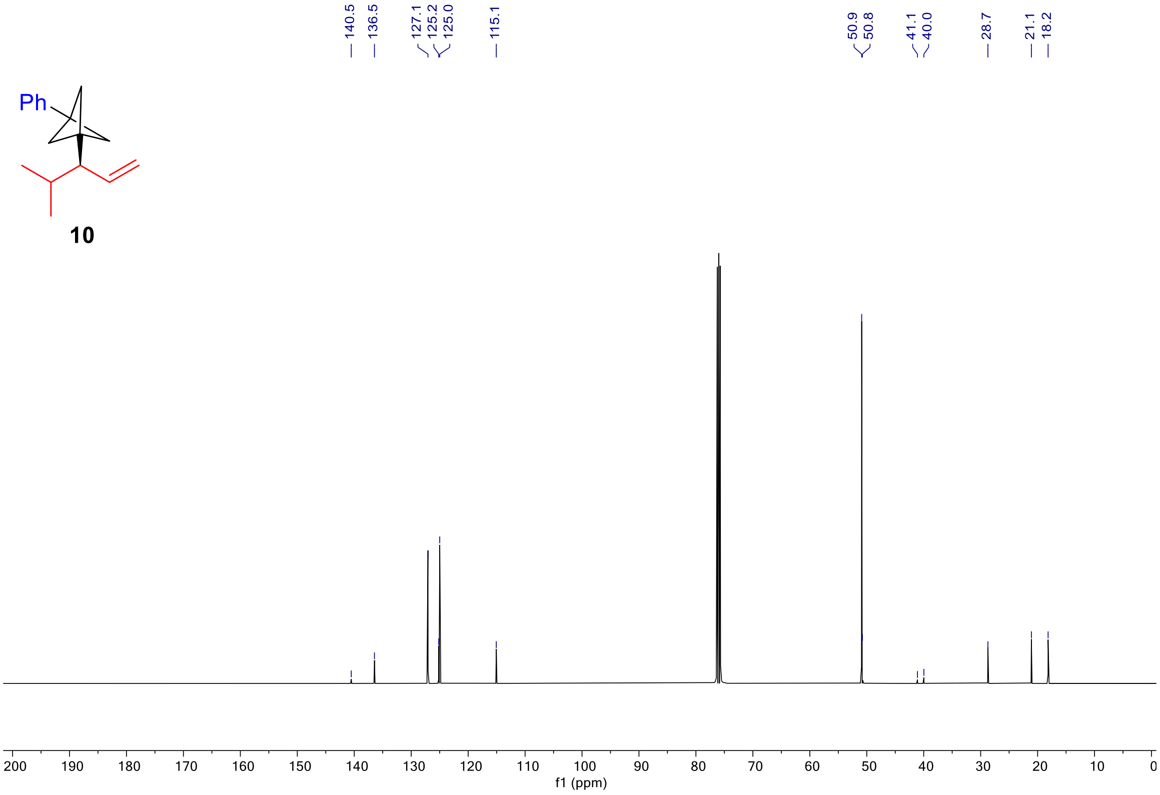

1H-NMR (CDCl3, 500 MHz)

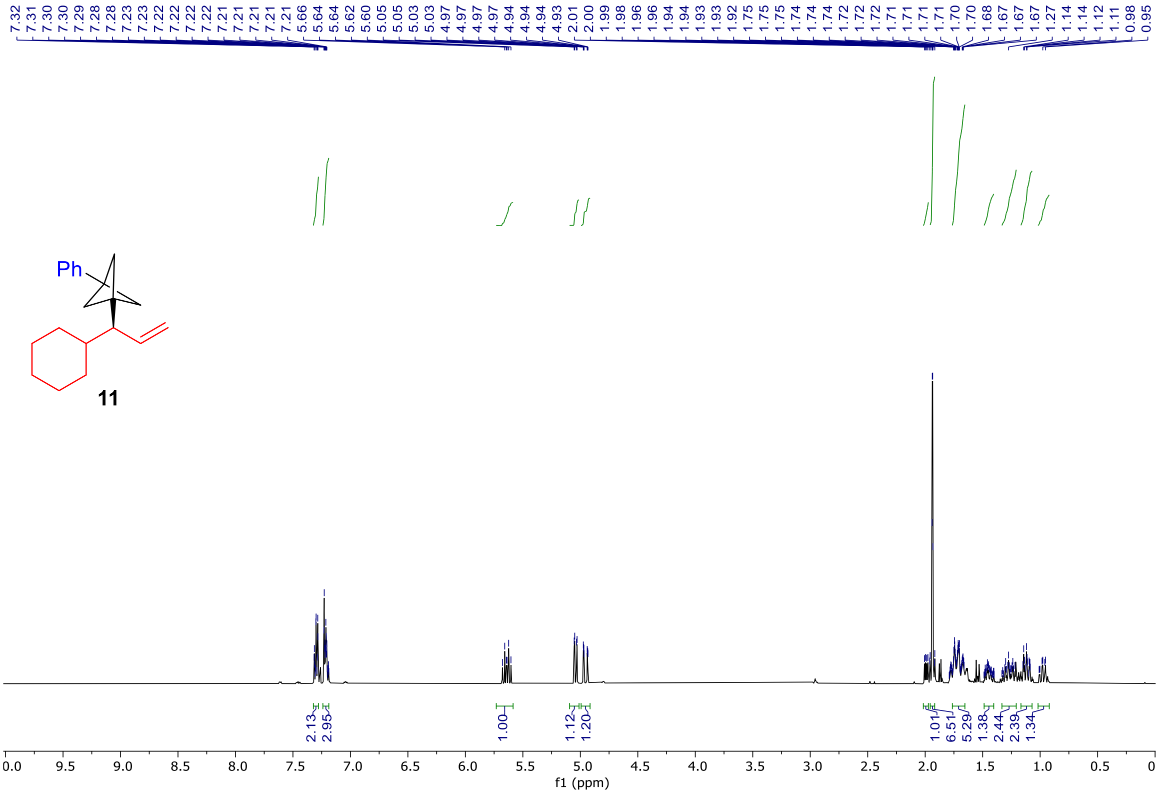

13C-NMR (CDCl3, 125 MHz)

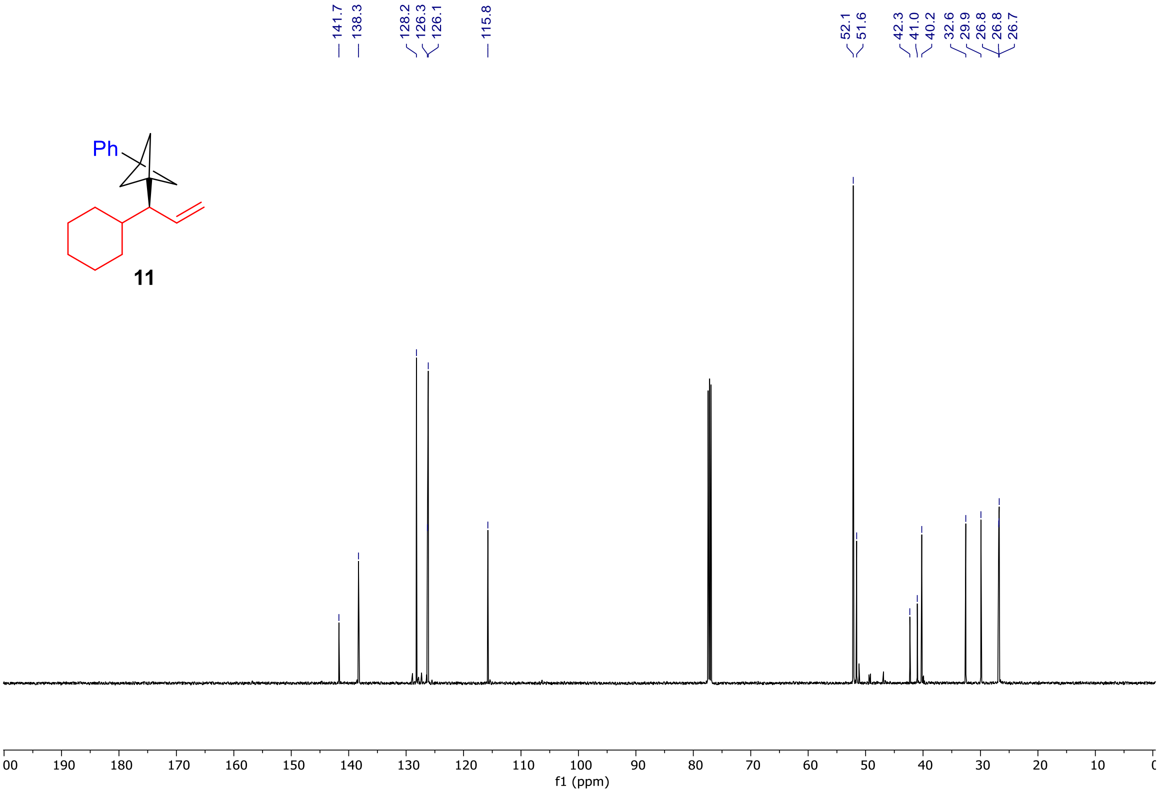

1H-NMR (CDCl3, 500 MHz)

12

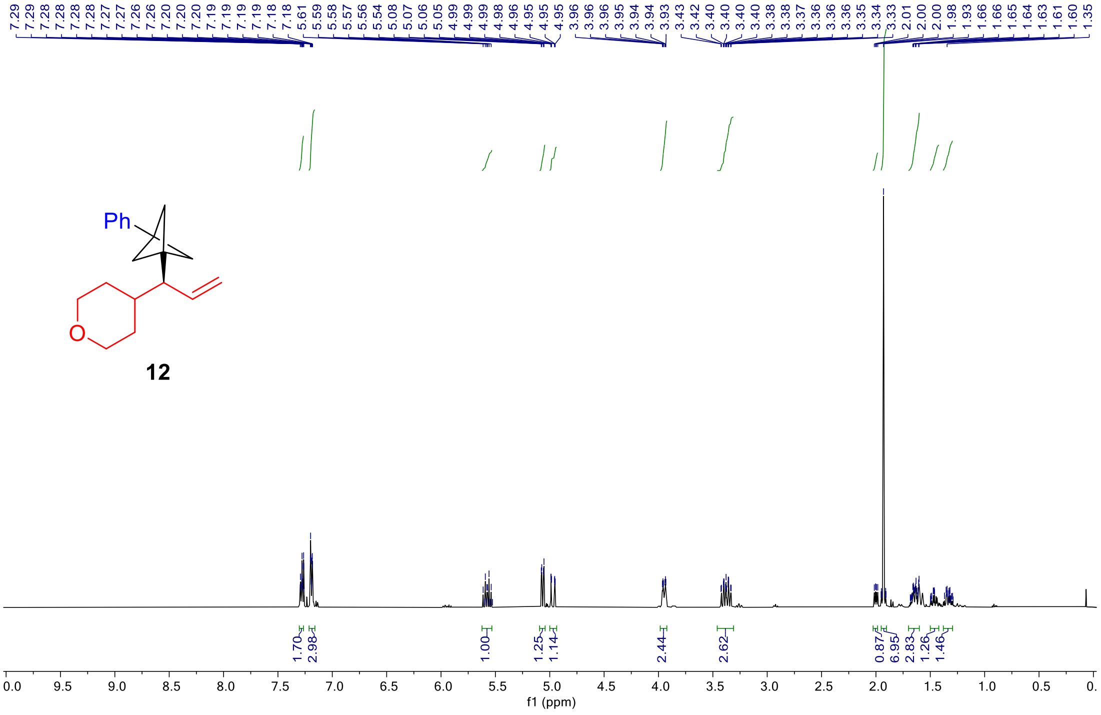

13C-NMR (CDCl3, 126 MHz)

12

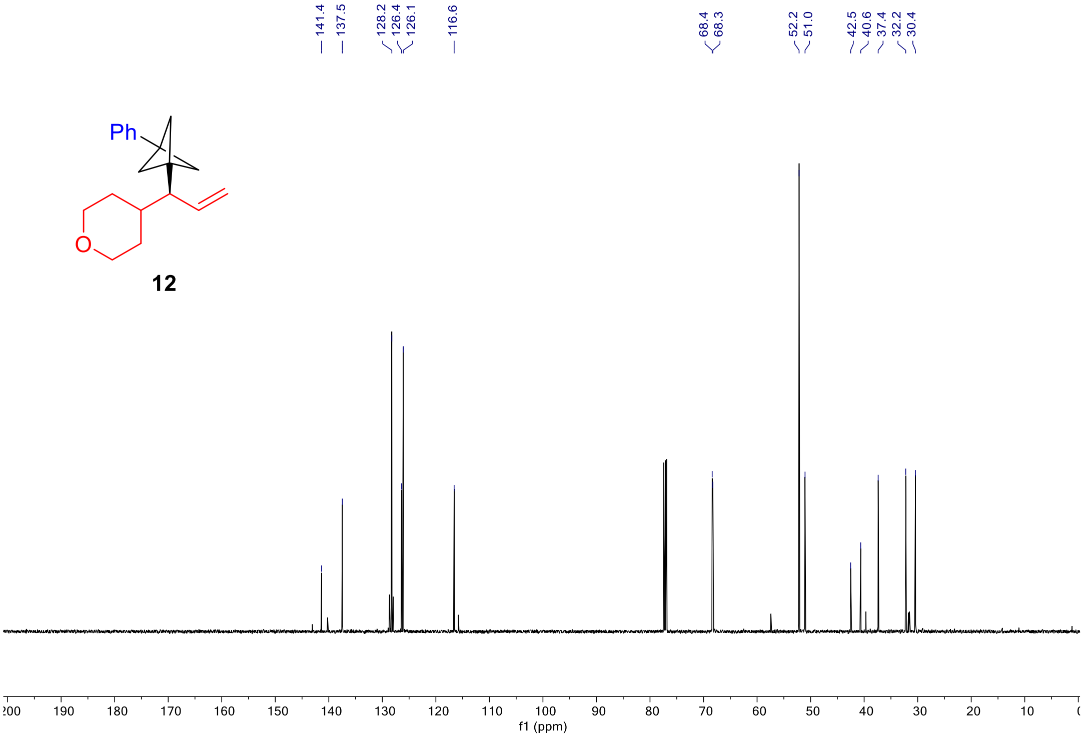

Note: extra peaks are related to the linear isomer (<5%)

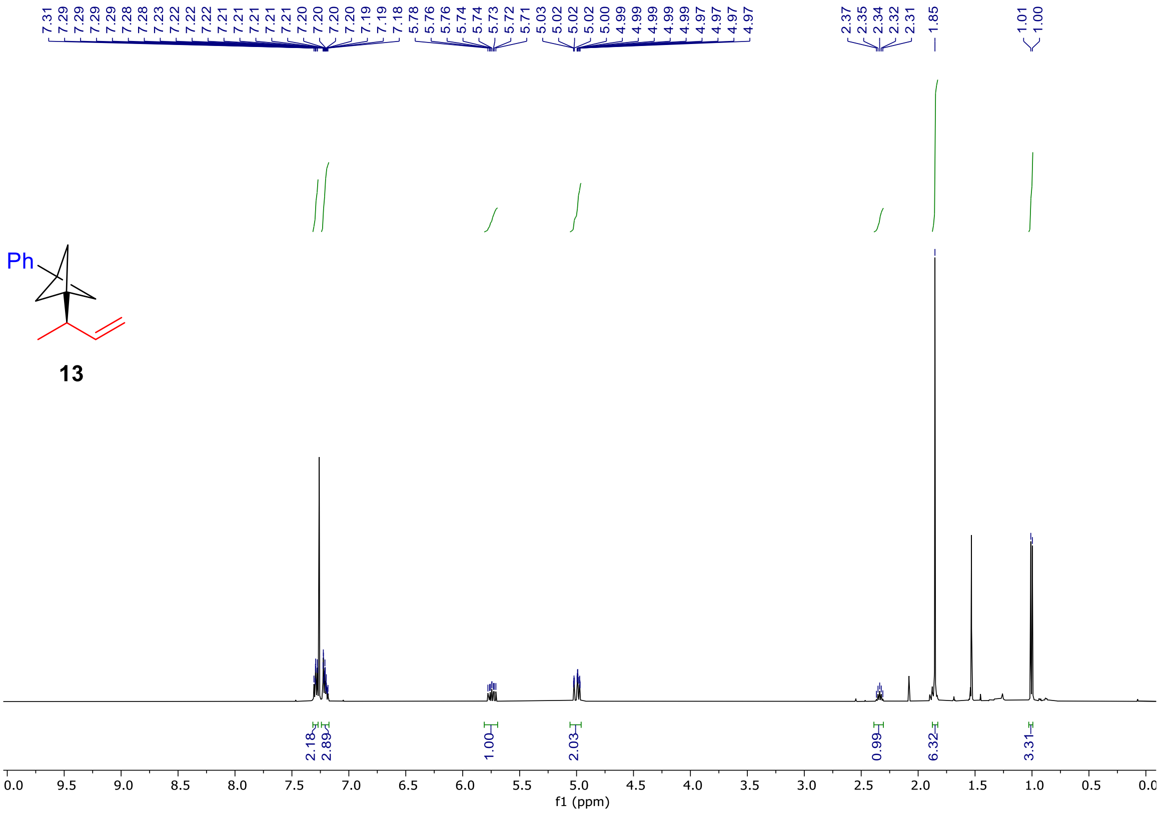

13C-NMR (CDCl3, 126 MHz)

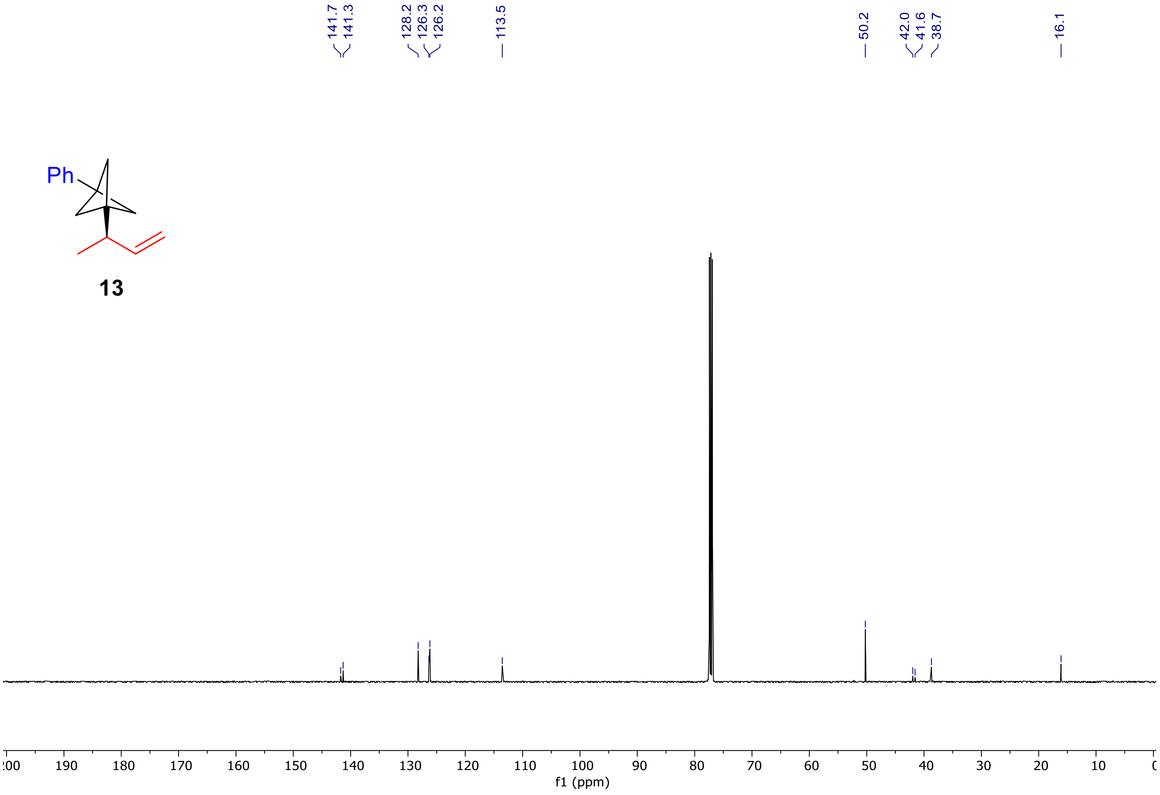

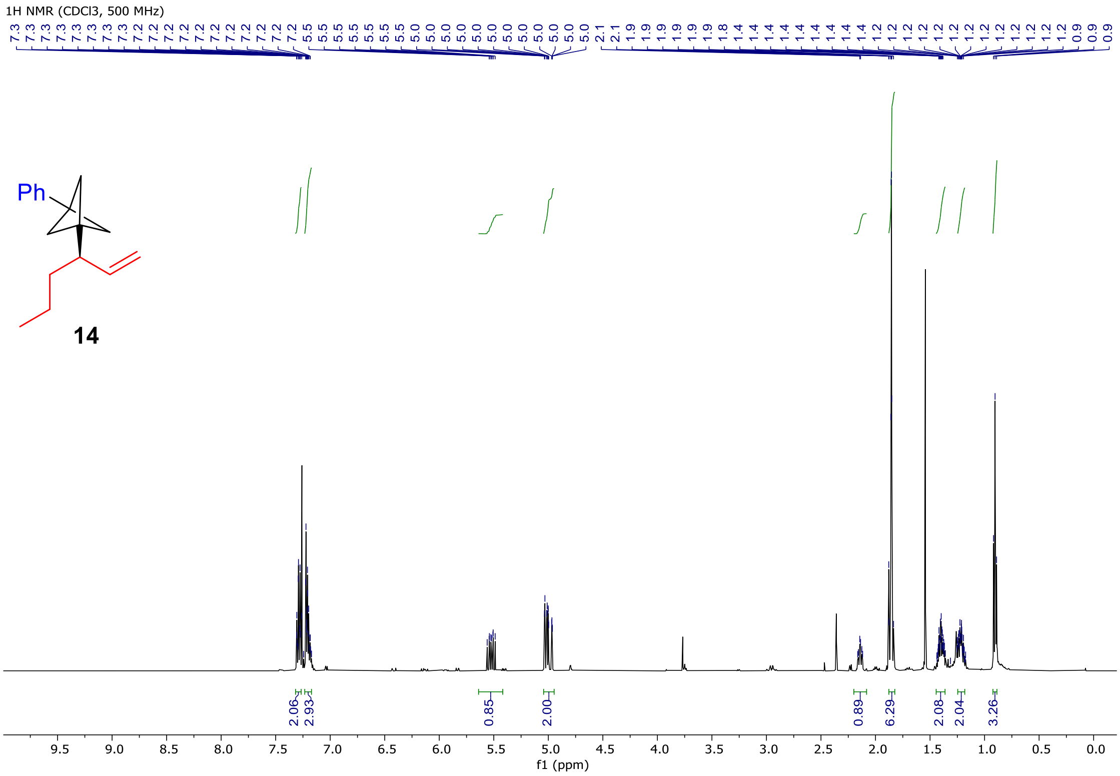

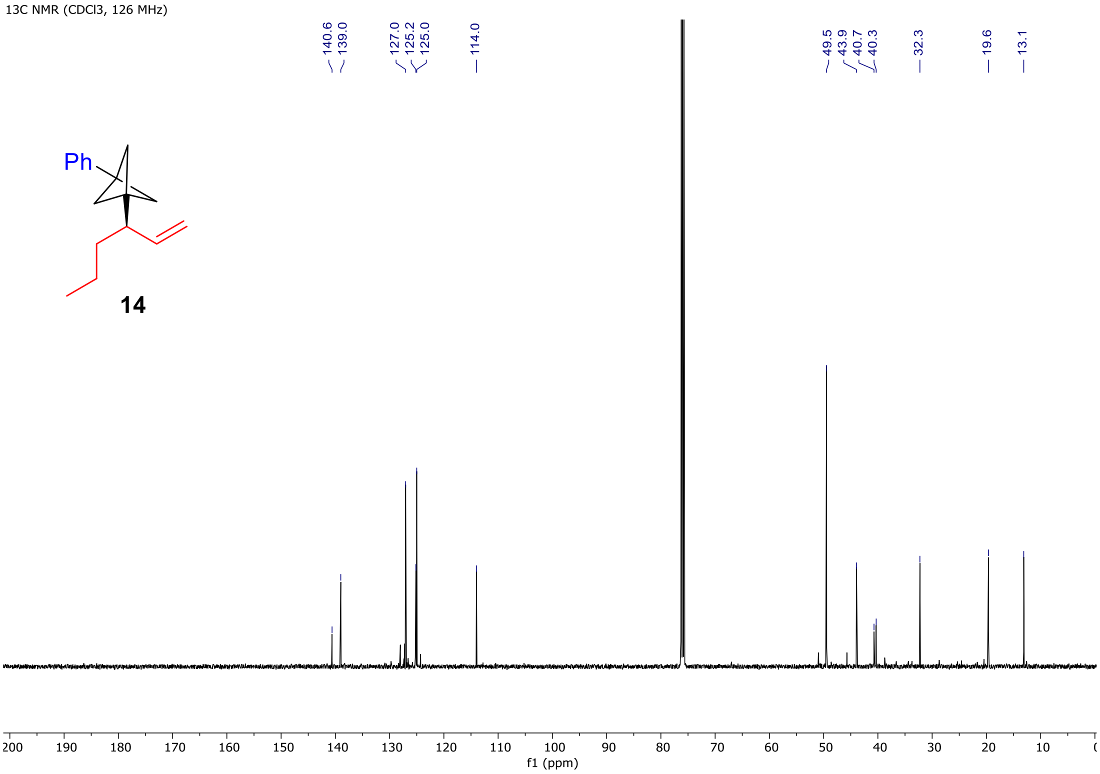

Note: extra peaks are related to the linear isomer (<5%)

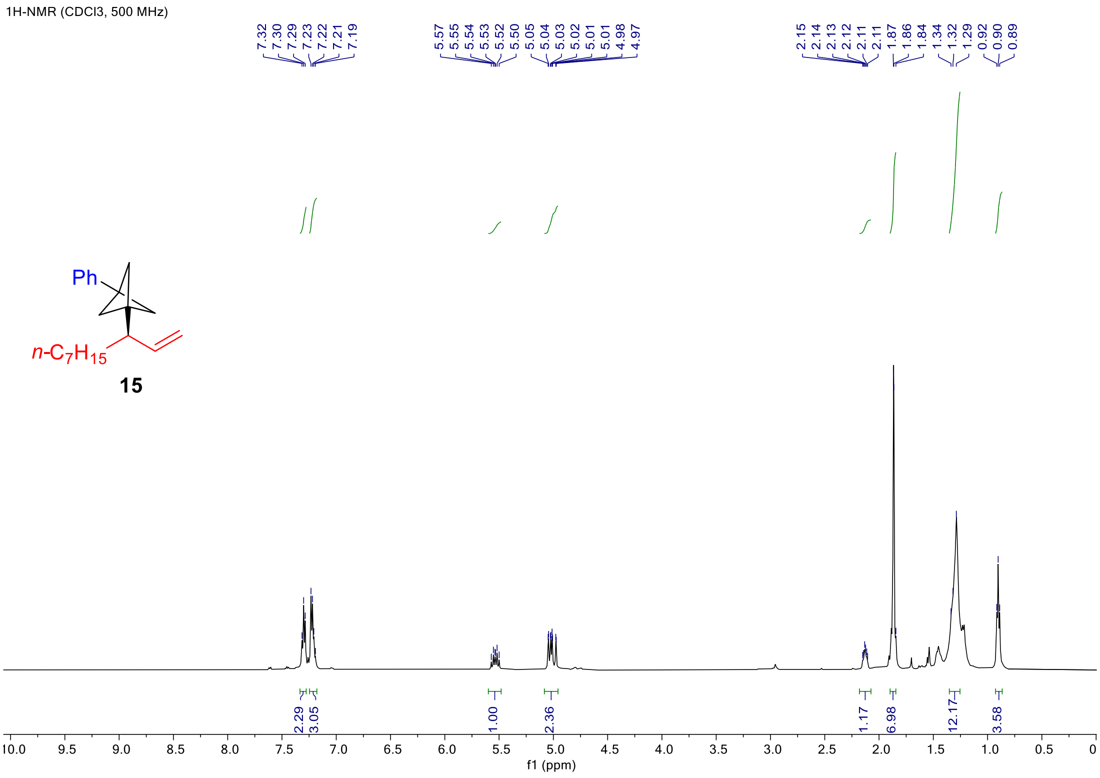

1H-NMR (CDCl3, 500 MHz)
7.32
7.30
7.29
7.23
7.22
7.21
7.19
5.57
5.55
5.54
5.53
5.52
5.50
5.05
5.04
5.03
5.02
5.01
5.01
4.98
4.97
2.15
2.14
2.13
2.12
2.11
2.11
1.87
1.86
1.84
1.34
1.32
1.29
0.92
0.90
0.89
Ph
n-C7H15
15
2.29
3.05
1.00
2.36
1.17
6.98
12.17
3.58
f1 (ppm)

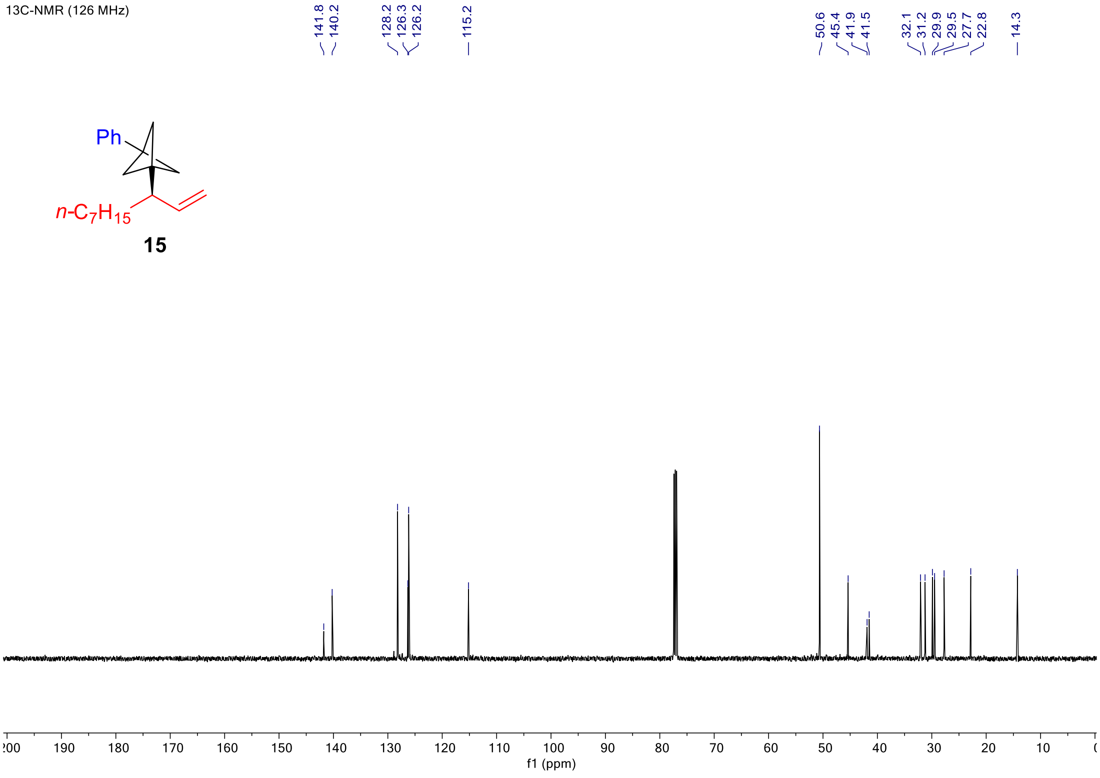

13C-NMR (126 MHz)
141.8
140.2
128.2
126.3
126.2
115.2
50.6
45.4
41.9
41.5
32.1
31.2
29.9
29.5
27.7
22.8
14.3
Ph
n-C7H15
15
f1 (ppm)

1H-NMR (CDCl3, 500 MHz)

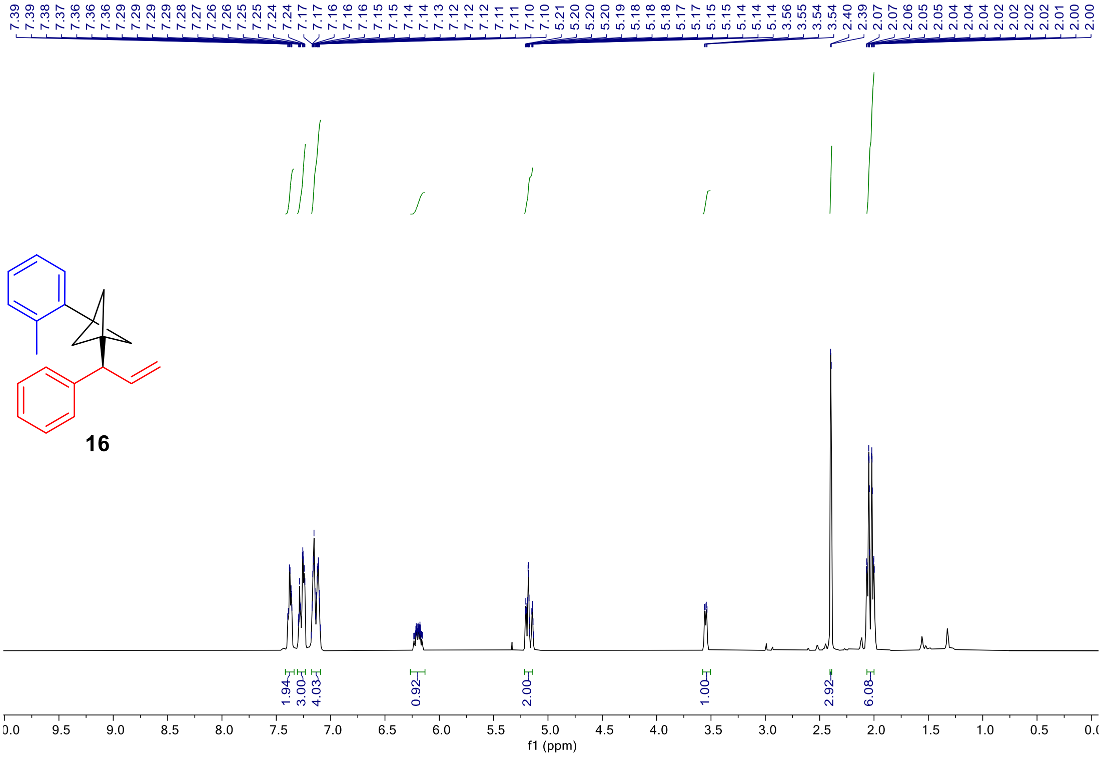

13C-NMR (CDCl3, 126 MHz)

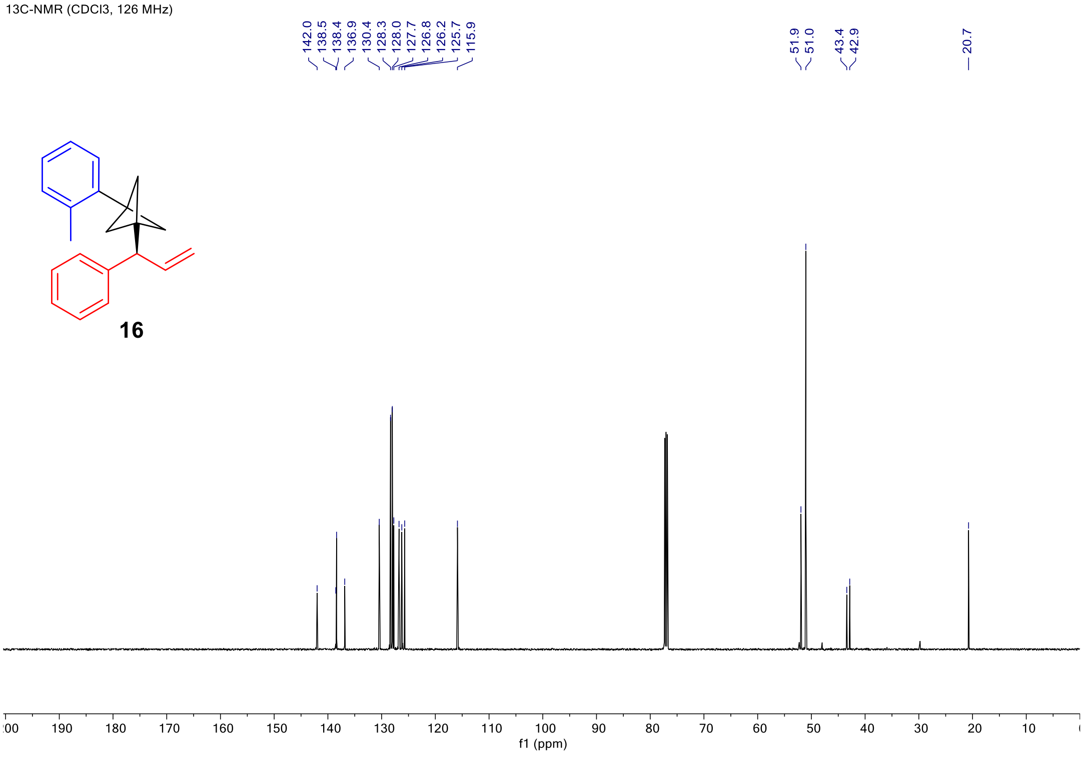

13C-NMR (CDCl3, 126 MHz)

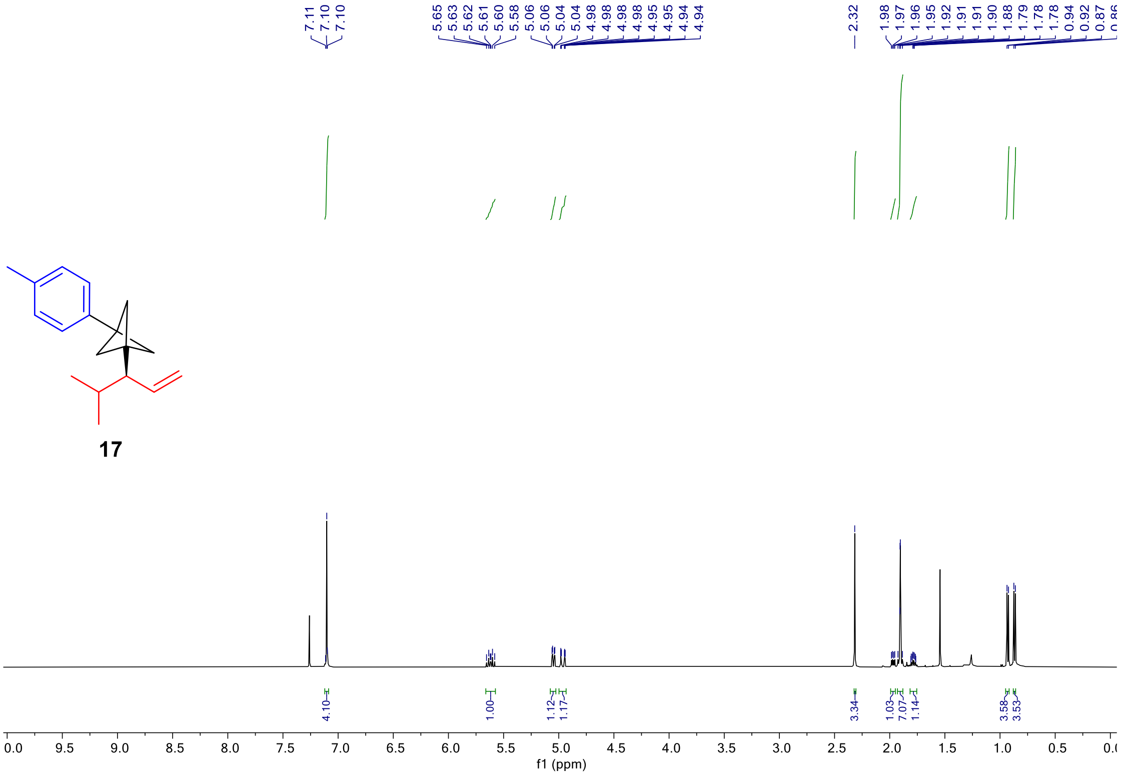

13C-NMR (CDCl3, 126 MHz)

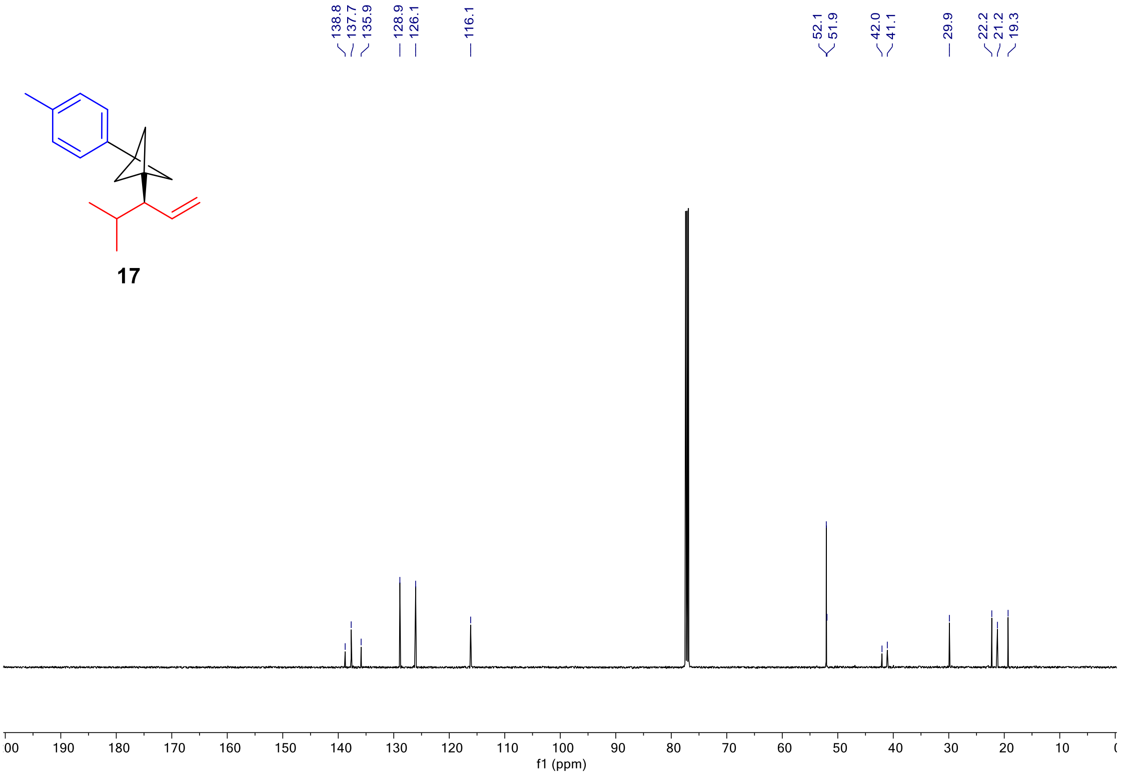

1H NMR (CDCl3, 500 MHz)

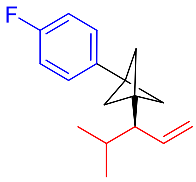

18

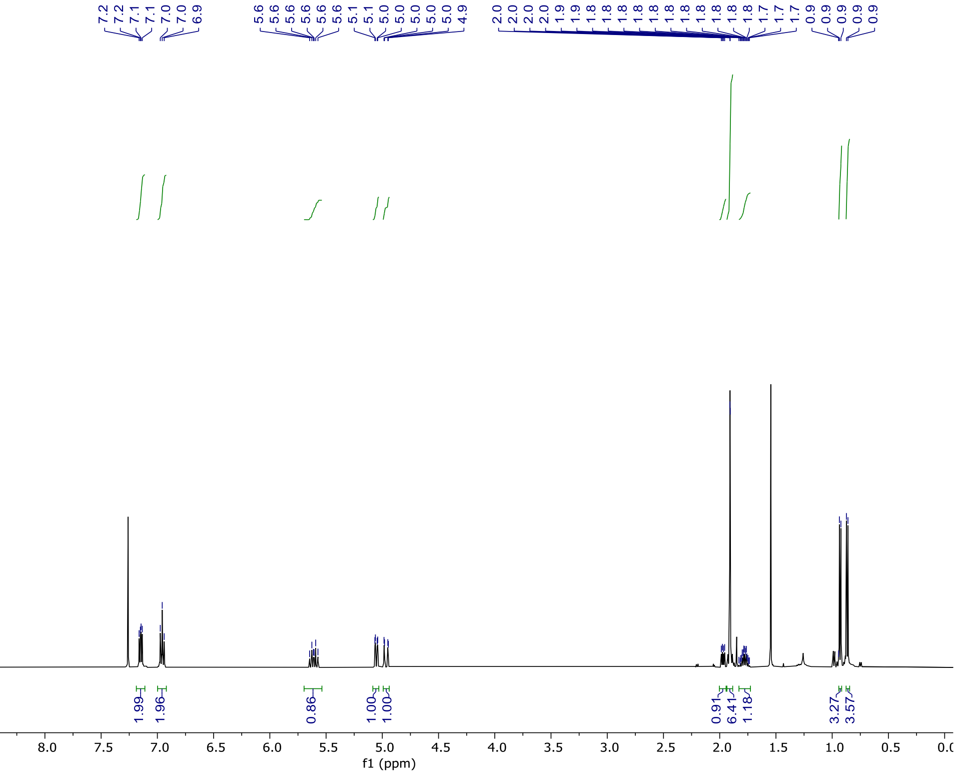

13C NMR (CDCl3, 126 MHz)

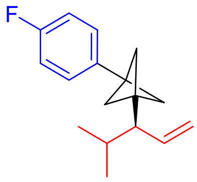

18

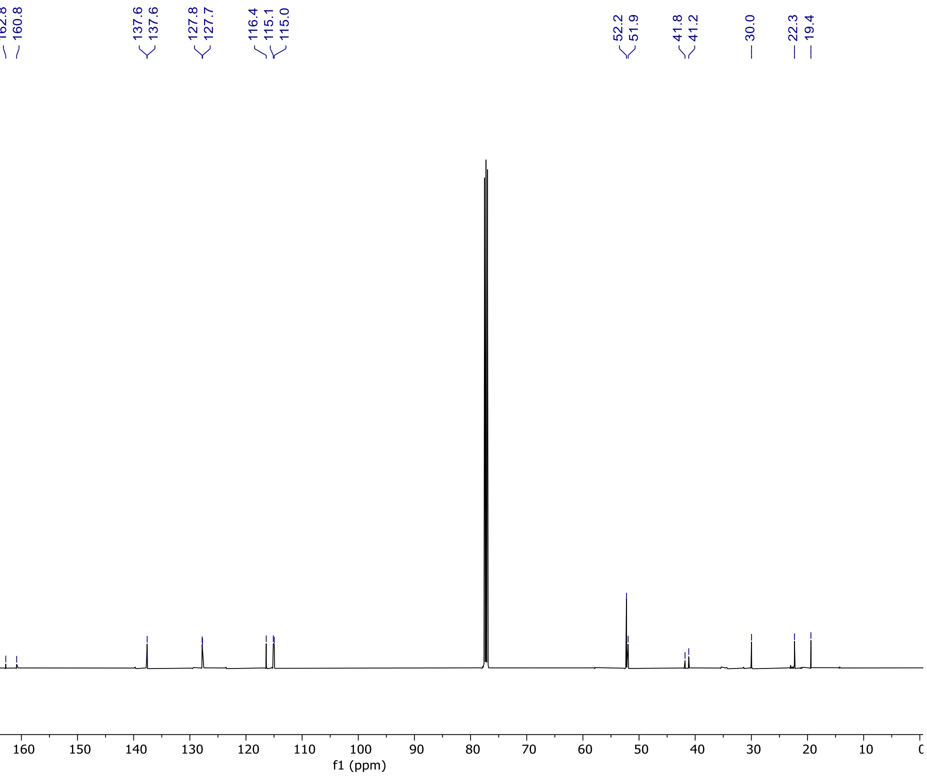

19F NMR (CDCl3, 282 MHz)

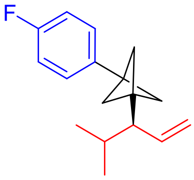

18

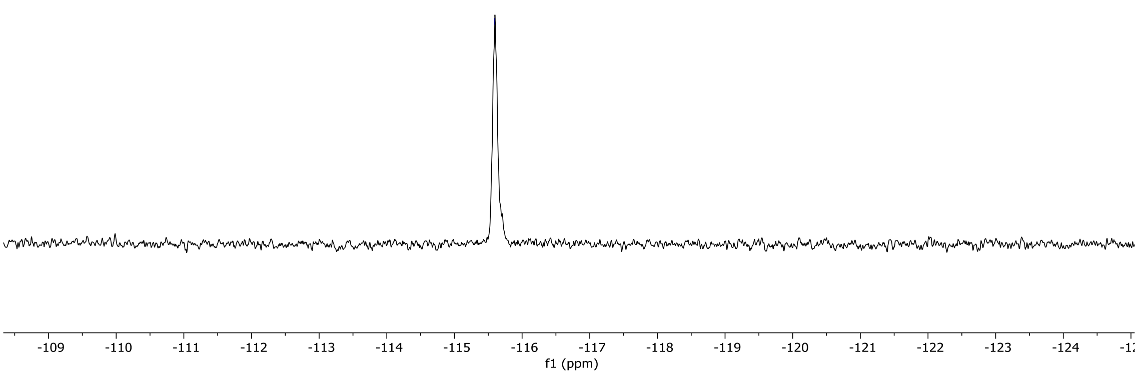

1H-NMR (CDCl3, 300 MHz)

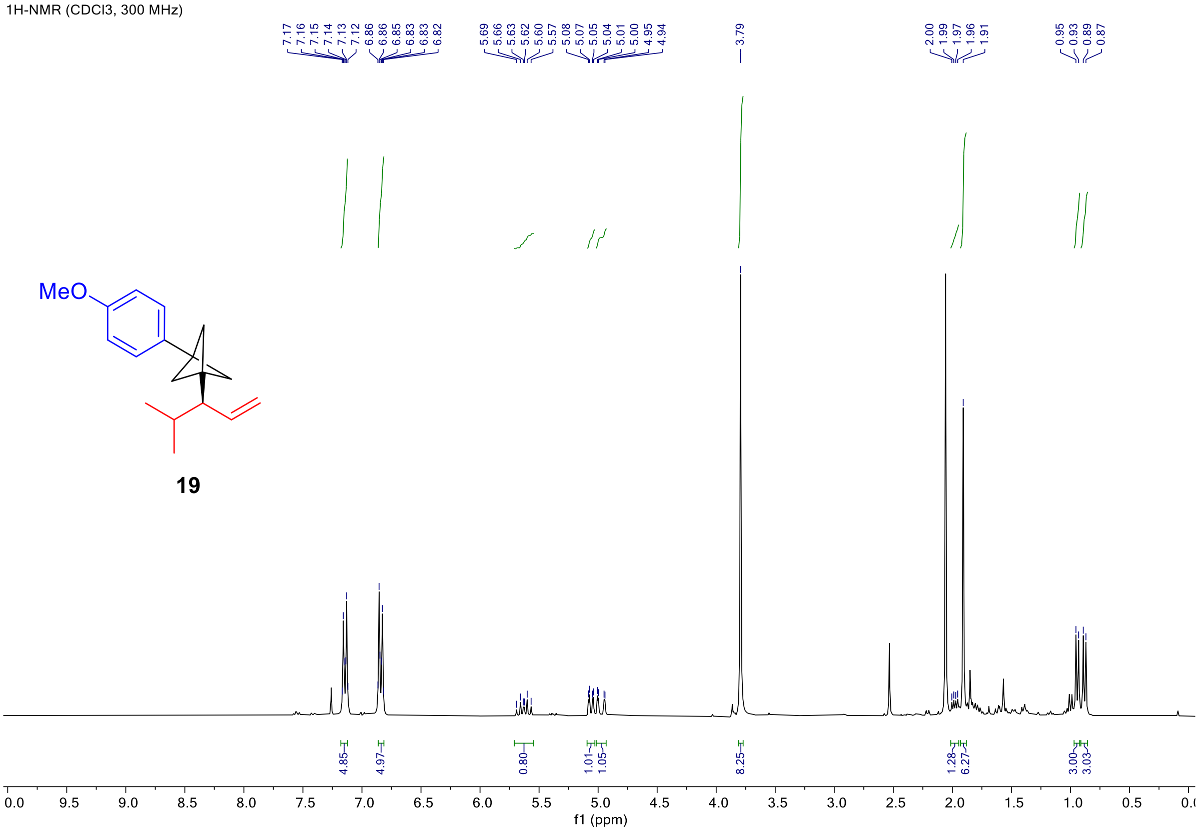

13C-NMR (CDCl3, 126 MHz)

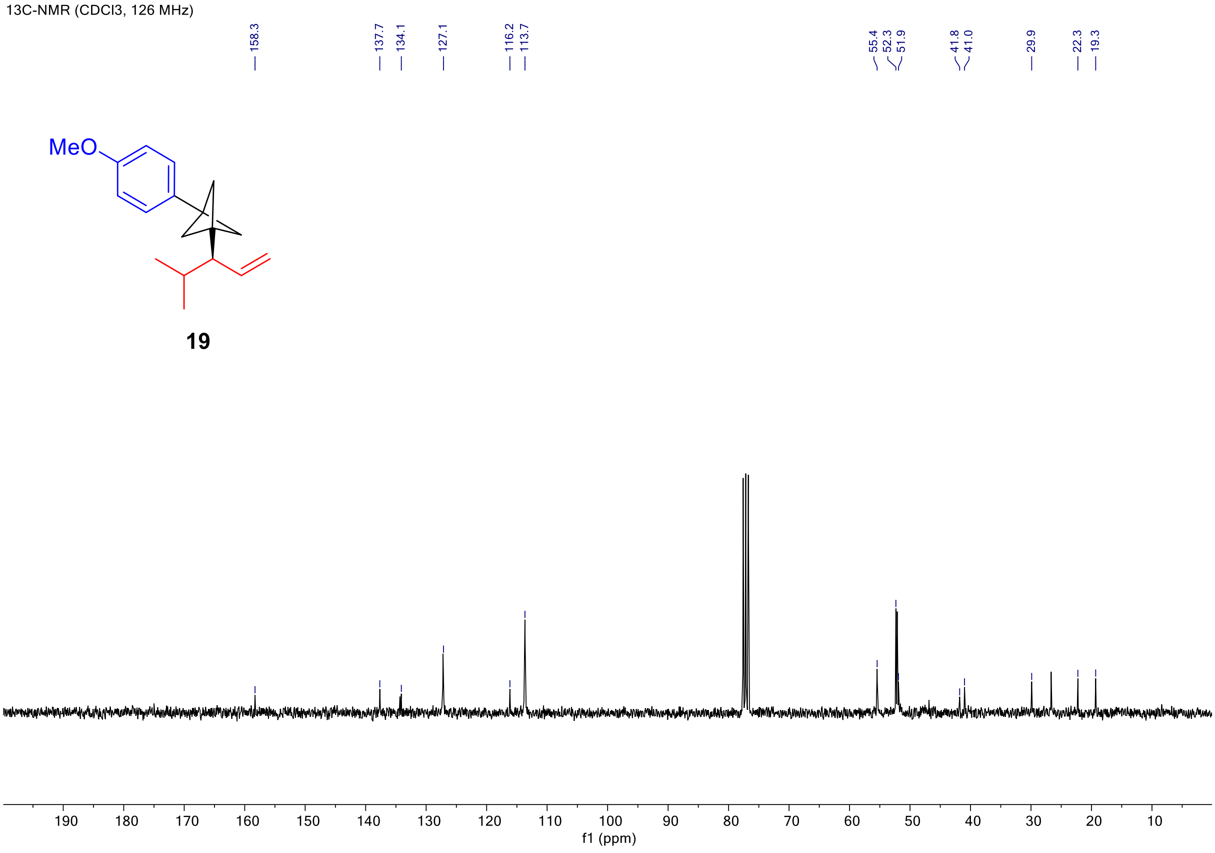

Note: extra peaks are related to the protonated BCP-Grignard

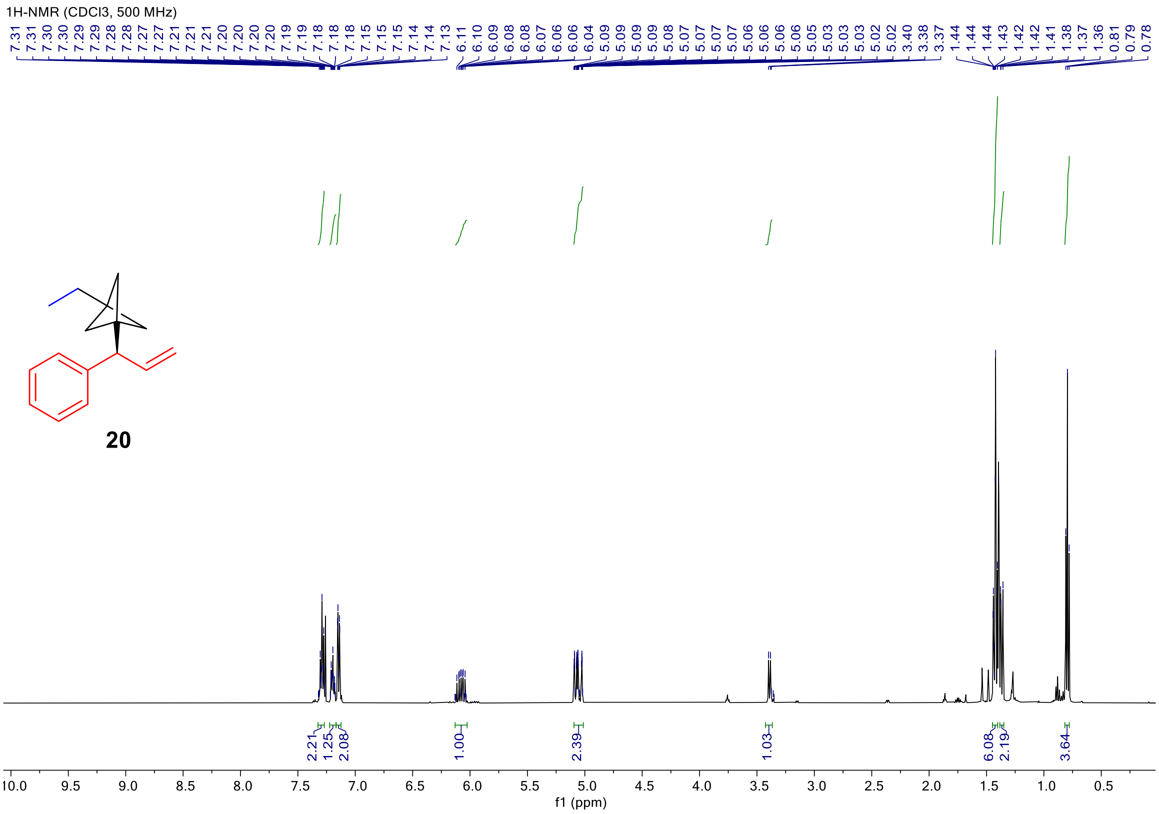
1H-NMR (CDCl3, 500 MHz)
7.31 7.31 7.30 7.29 7.29 7.28 7.28 7.27 7.27 7.21 7.21 7.21 7.20 7.20 7.20 7.20 7.19 7.19 7.18 7.18 7.18 7.15 7.15 7.15 7.14 7.14 7.13 6.11 6.10 6.09 6.08 6.08 6.07 6.06 6.06 6.04 5.09 5.09 5.09 5.09 5.08 5.07 5.07 5.07 5.07 5.06 5.06 5.06 5.05 5.03 5.03 5.03 5.02 5.02 3.40 3.38 3.37 1.44 1.44 1.43 1.42 1.42 1.41 1.38 1.37 1.36 0.81 0.79 0.78
20
2.21 1.25 2.08 1.00 2.39 1.03 6.08 2.19 3.64
10.0 9.5 9.0 8.5 8.0 7.5 7.0 6.5 6.0 5.5 5.0 4.5 4.0 3.5 3.0 2.5 2.0 1.5 1.0 0.5
f1 (ppm)

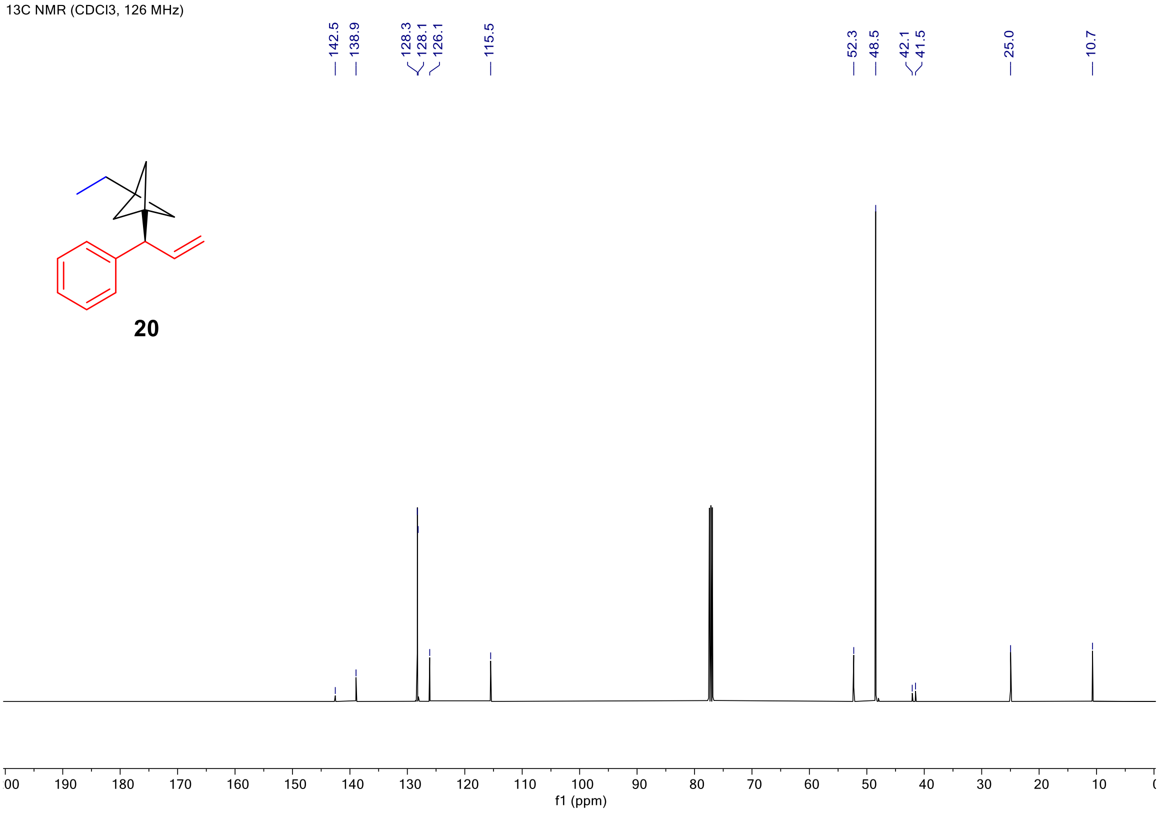
13C NMR (CDCl3, 126 MHz)
142.5 138.9 128.3 128.1 126.1 115.5 52.3 48.5 42.1 41.5 25.0 10.7
20
00 190 180 170 160 150 140 130 120 110 100 90 80 70 60 50 40 30 20 10
f1 (ppm)

1H NMR (CDCl3, 500 MHz)

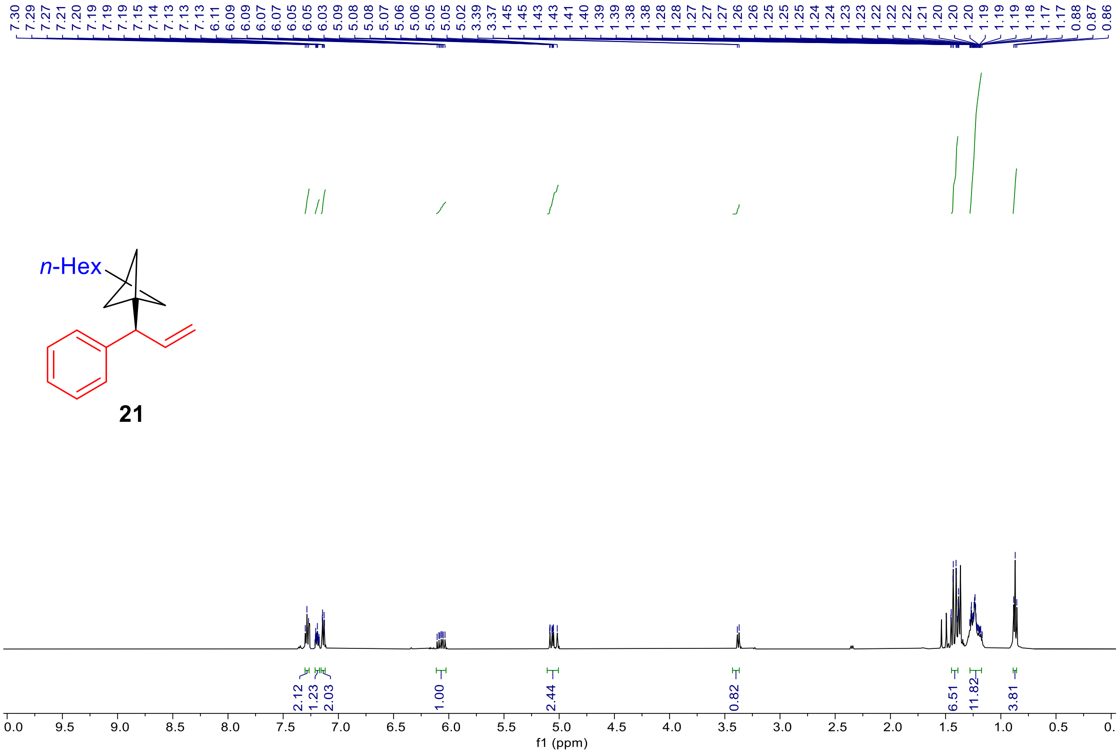

13C NMR (CDCl3, 500 MHz)

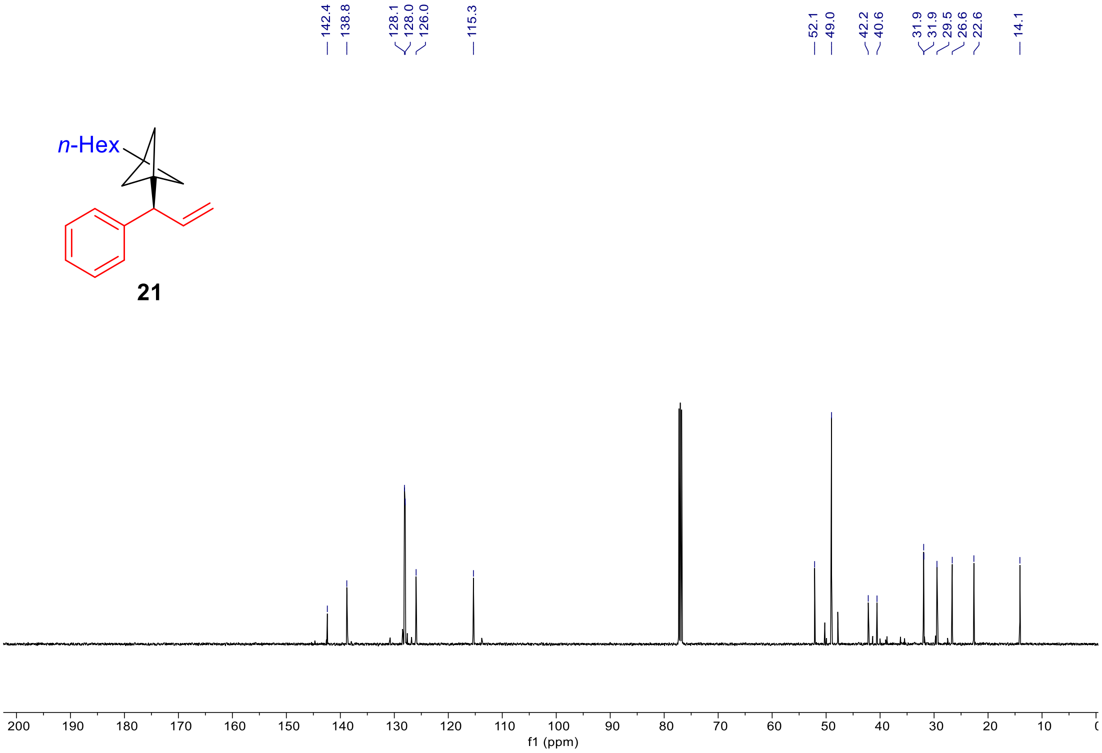

1H-NMR (CDCl3, 500 MHz)

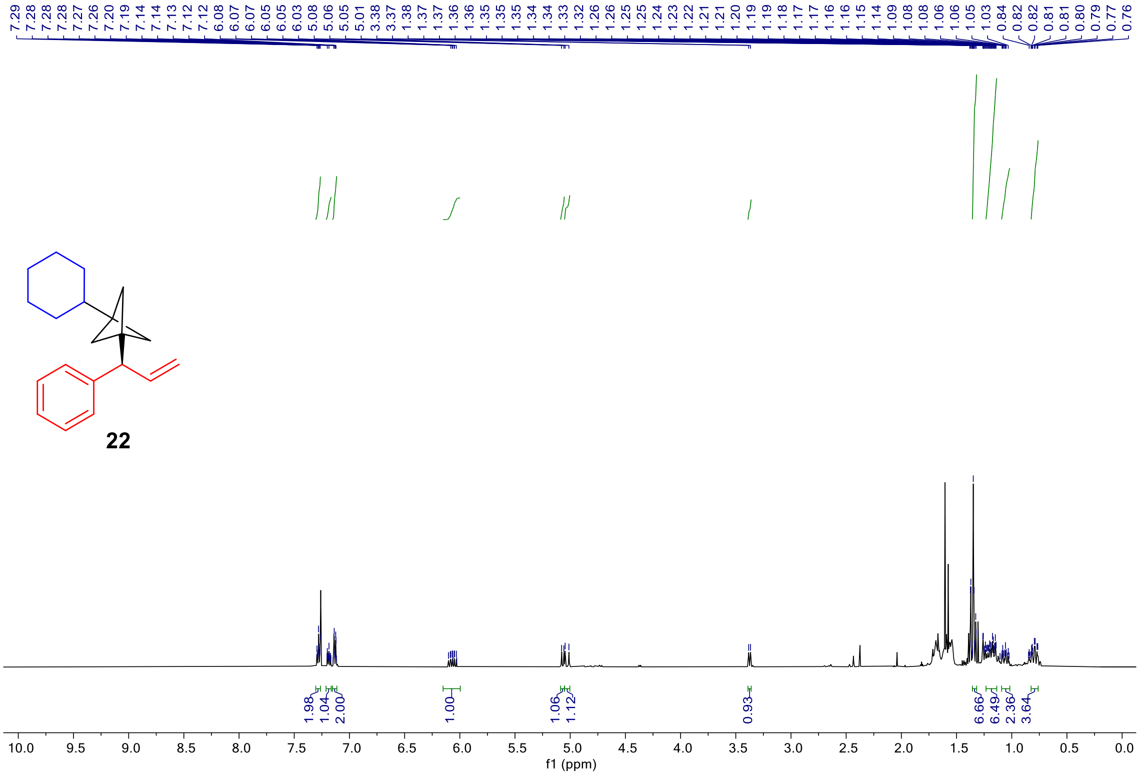

13C-NMR (CDCL3, 126 MHz)

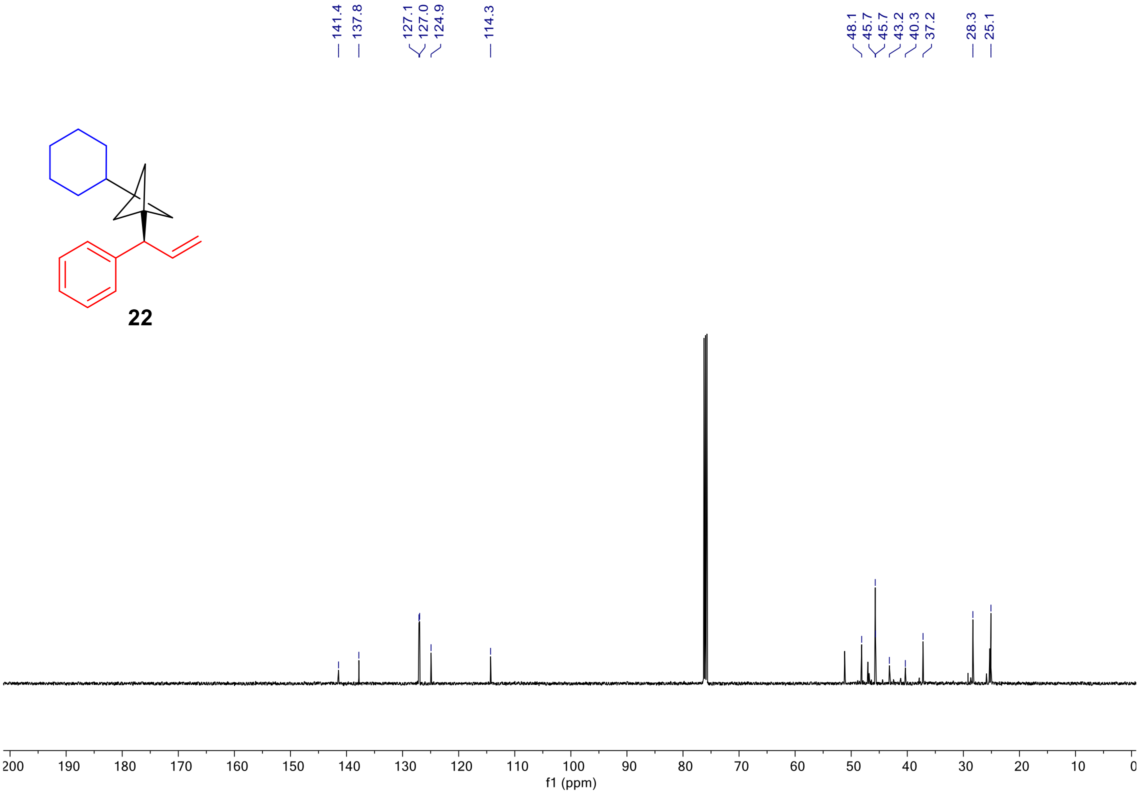

Note: extra peaks are related to the protonated BCP-Grignard

1H NMR (CDCl3, 500 MHz)

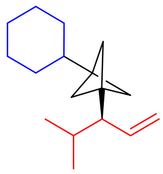

23

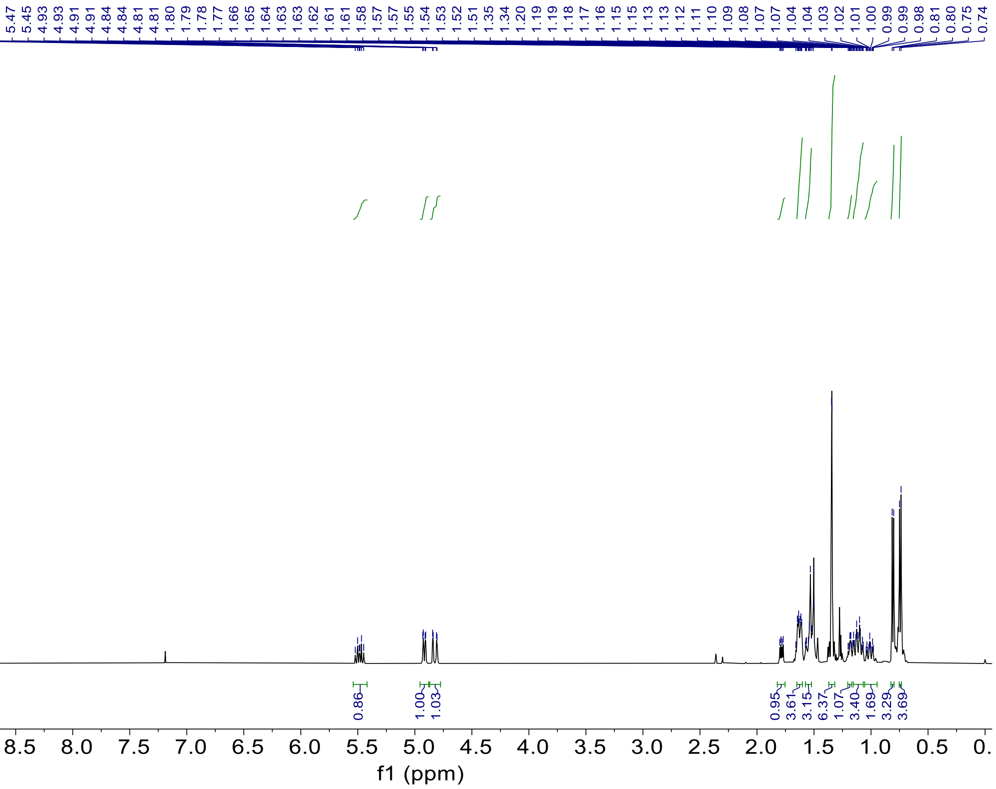

13C NMR (CDCl3, 126 MHz)

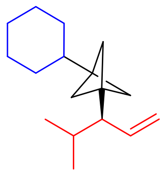

23

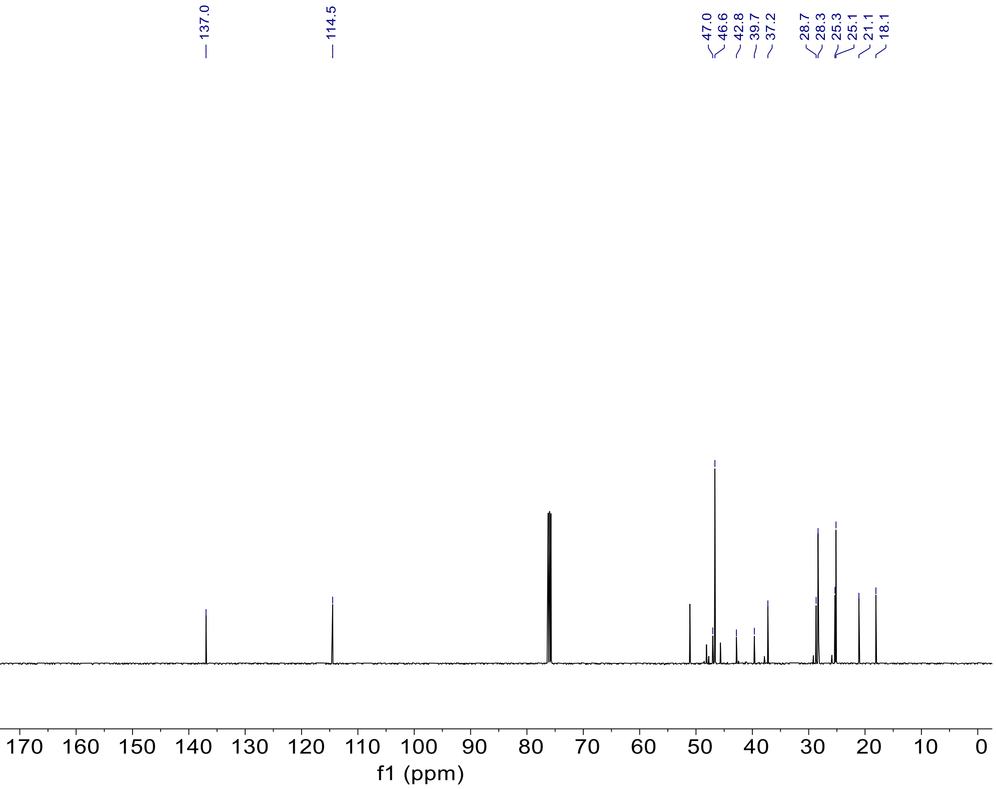

Note: extra peaks are related to the protonated BCP-Grignard

1H-NMR (CDCl3, 500 MHz)

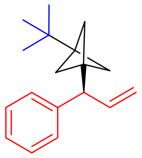

24

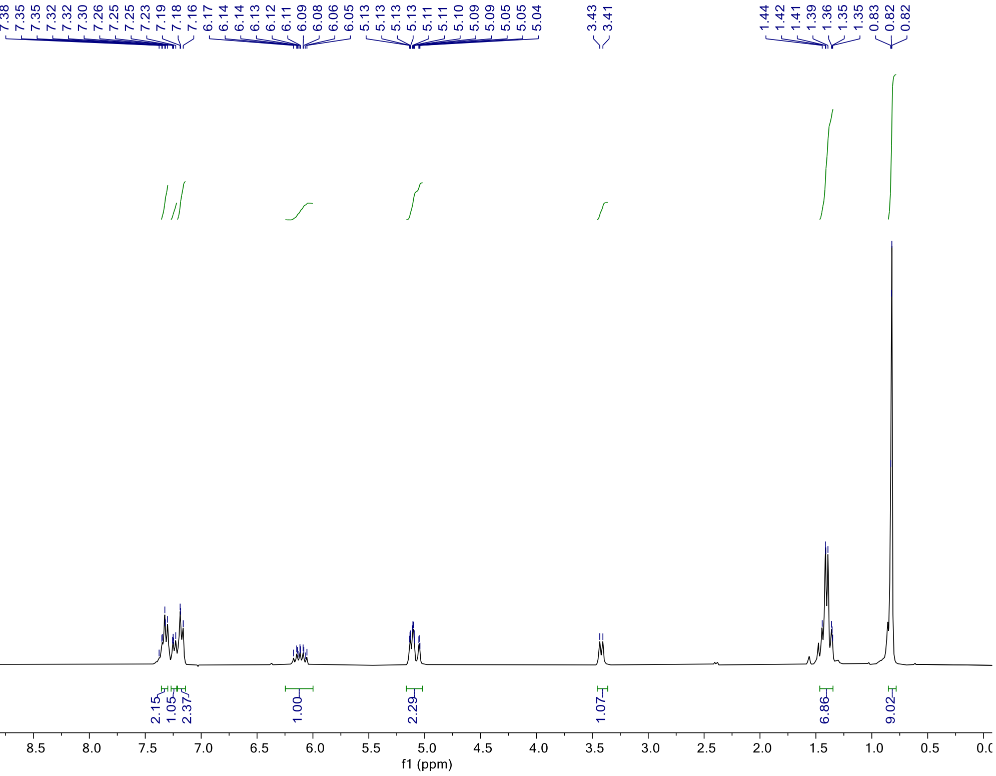

13C-NMR (CDCl3, 126 MHz)

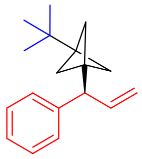

24

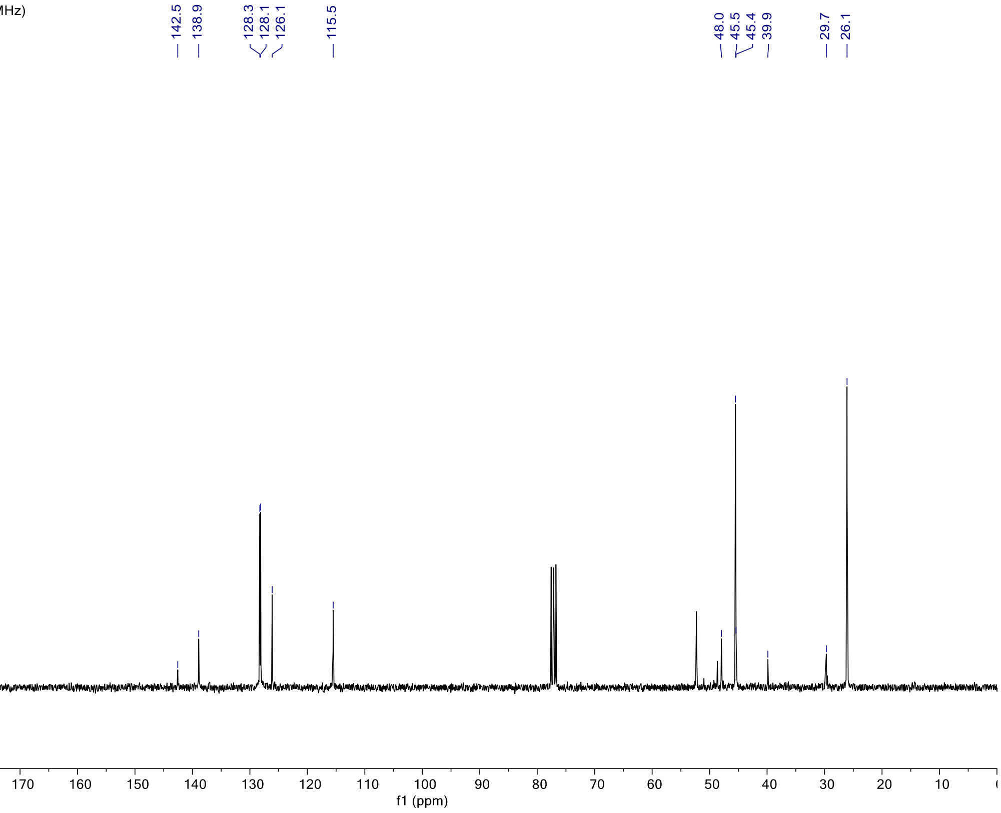

1H-NMR (500 MHz)

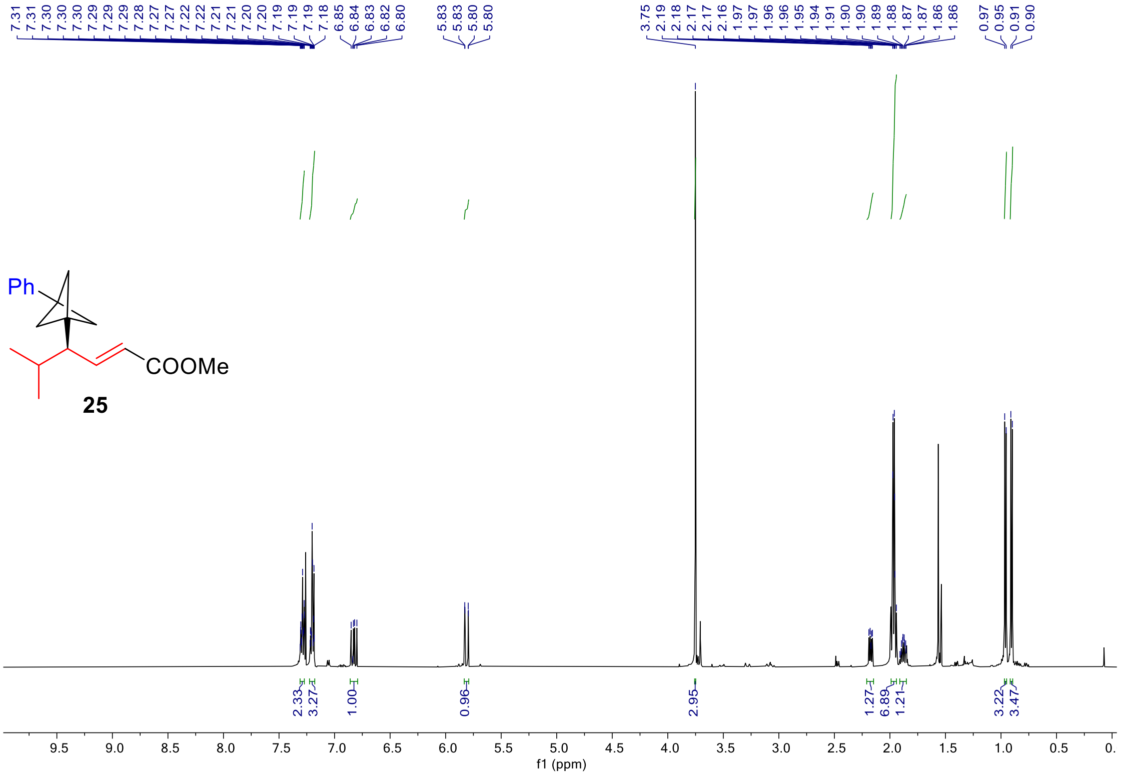

13C-NMR (CDCl3, 126 MHz)

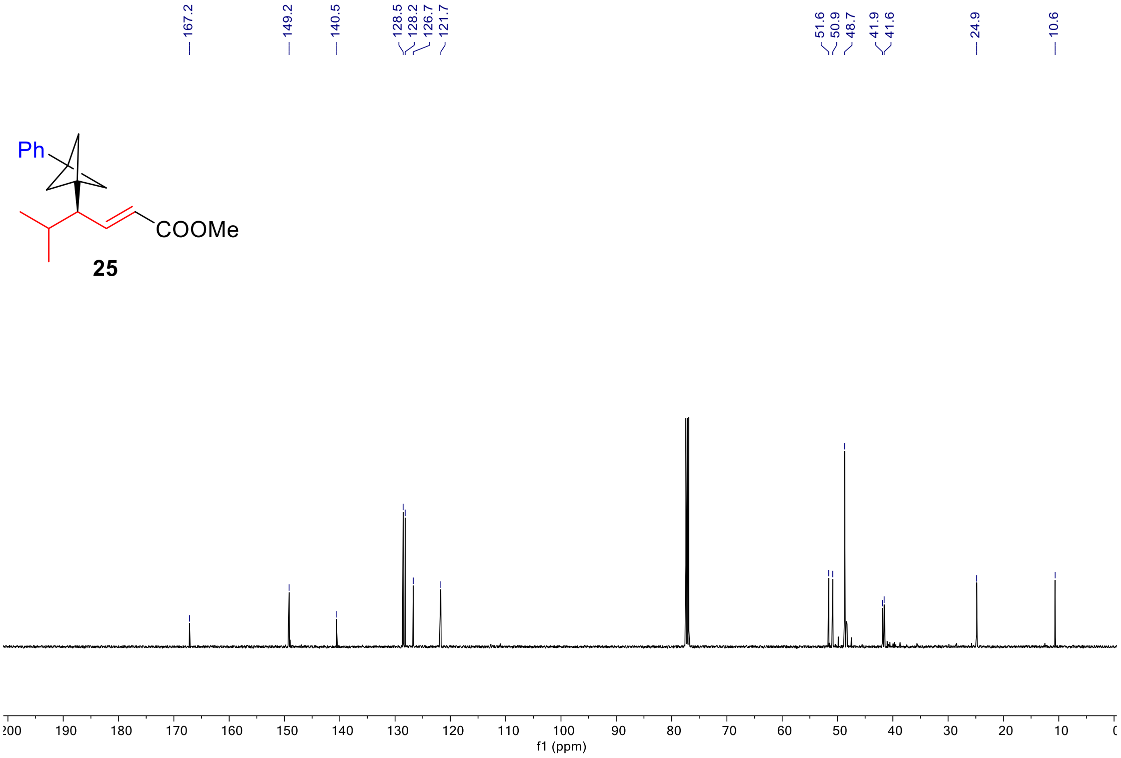

1H-NMR (CDCl3, 500 MHz)

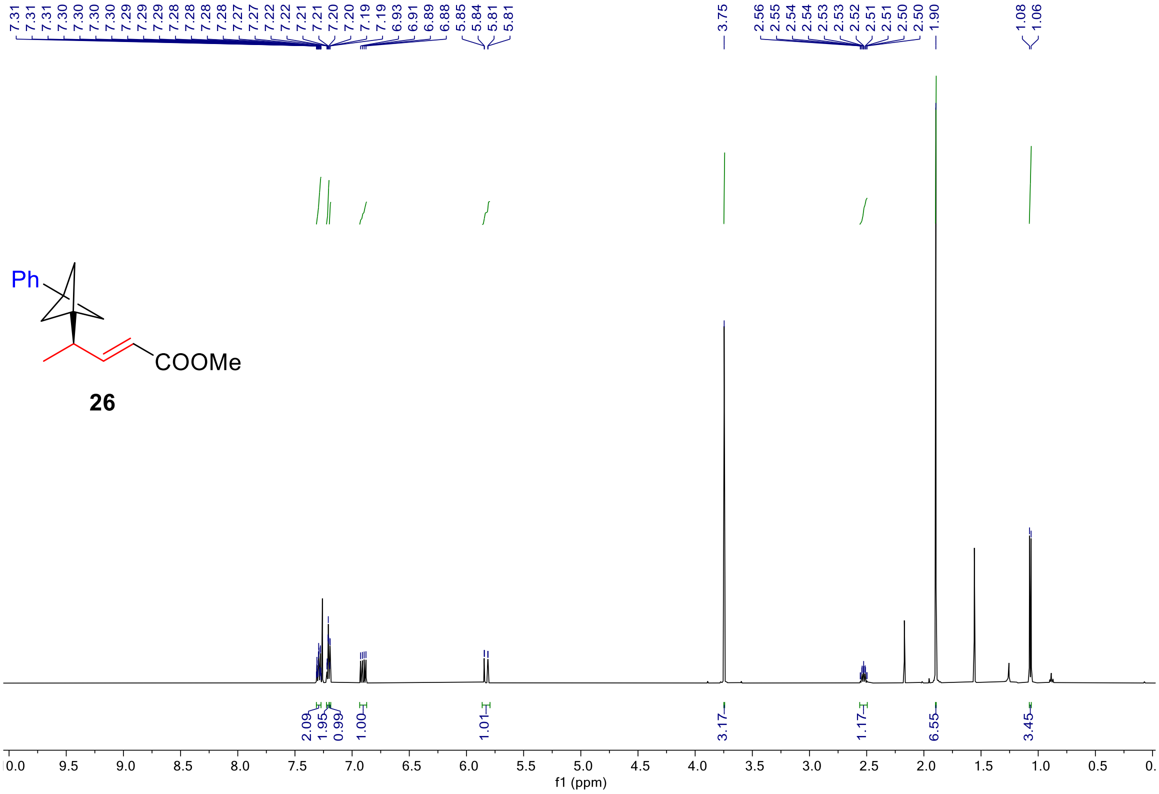

13C-NMR (CDCl3, 126 MHz)

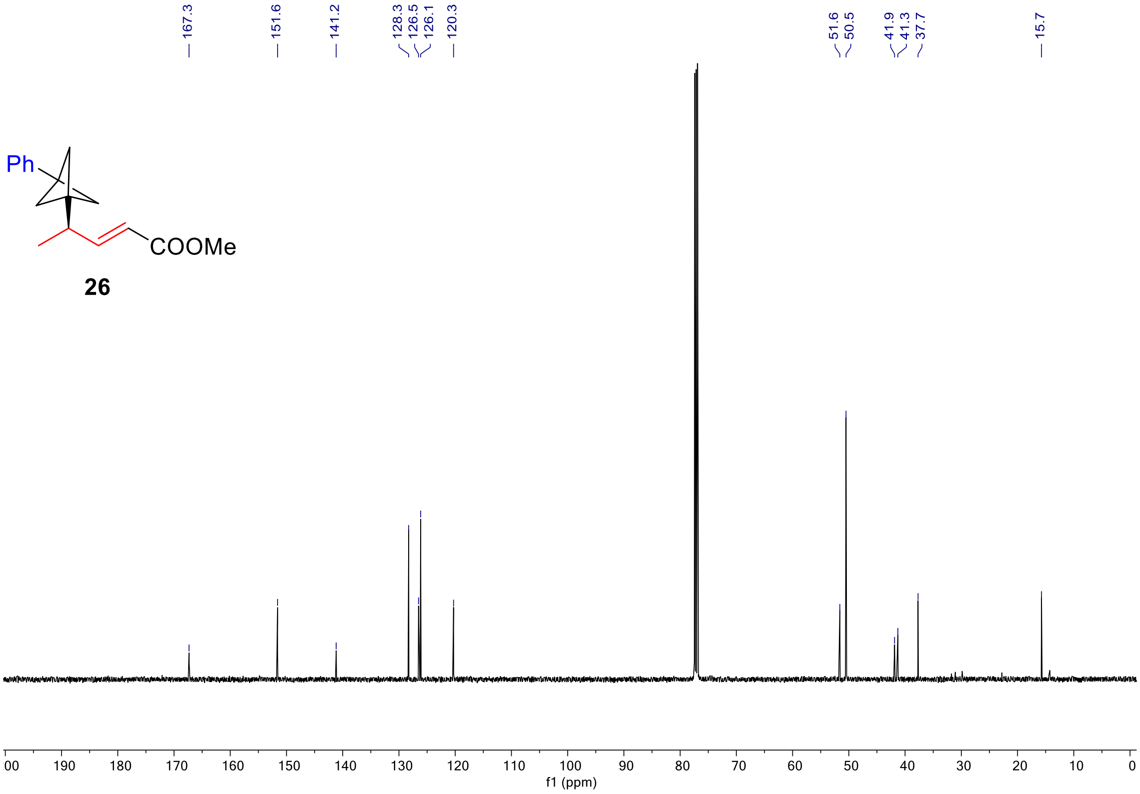

1H-NMR (CDCl3, 500 MHz)

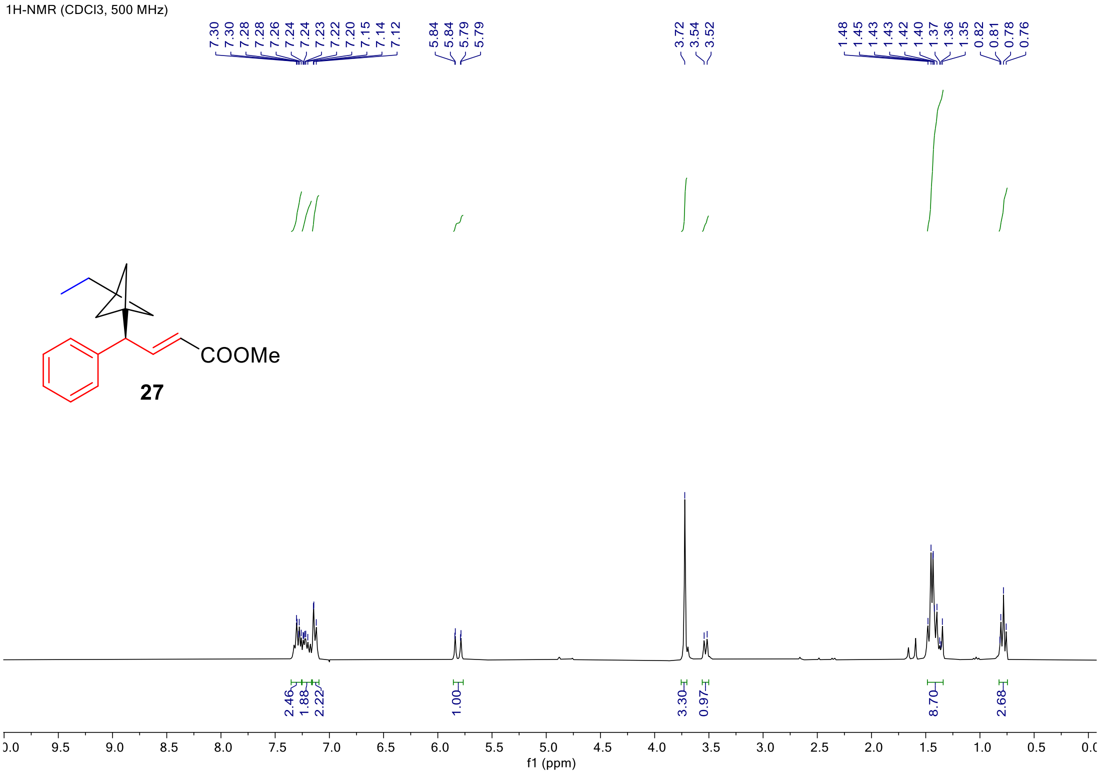

13C-NMR (CDCl3, 126 MHz)

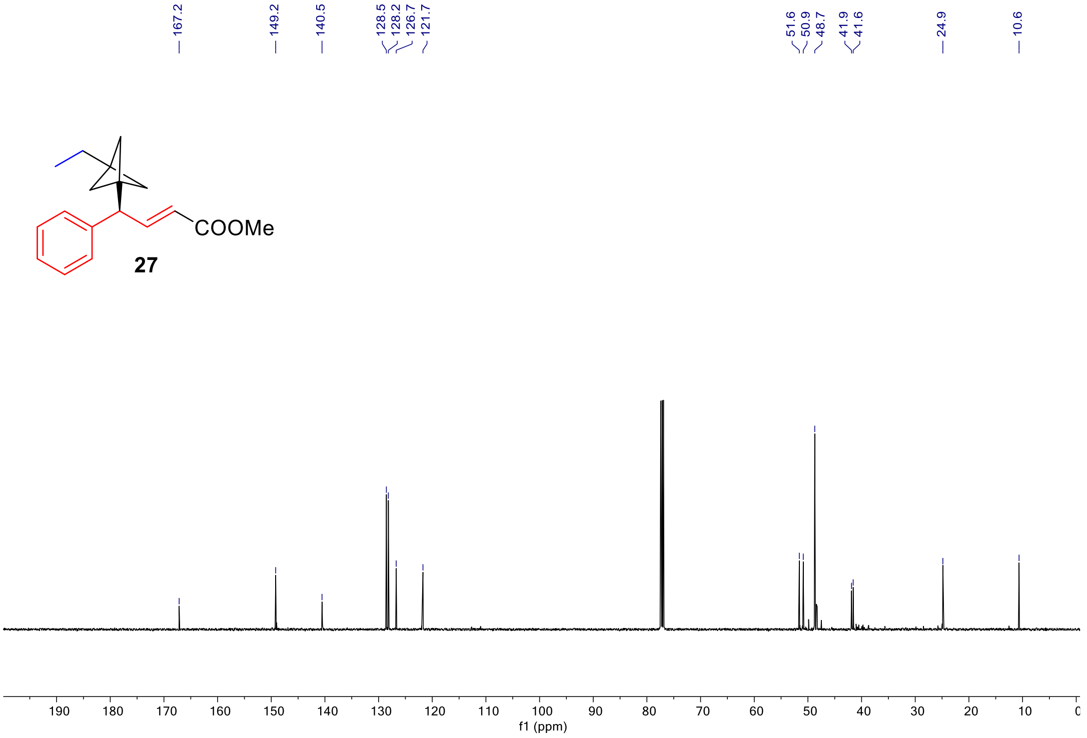

1H NMR (CDCl3, 500 MHz)

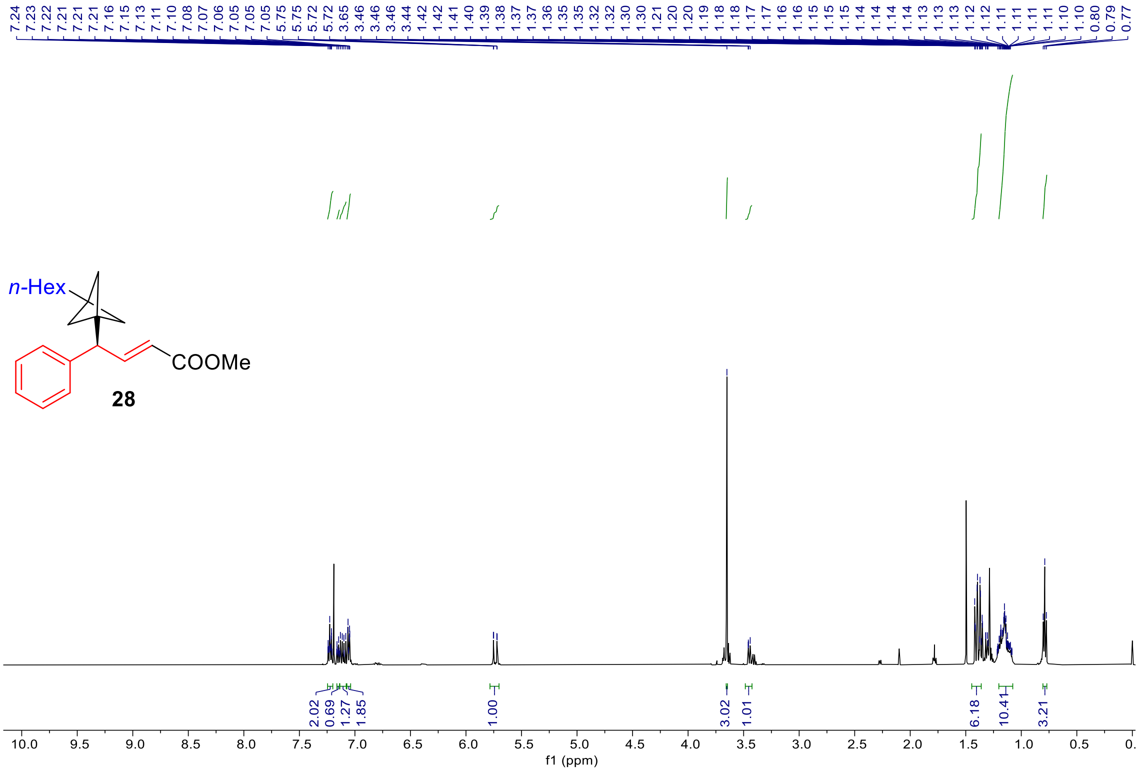

13C NMR (CDCl3, 126 MHz)

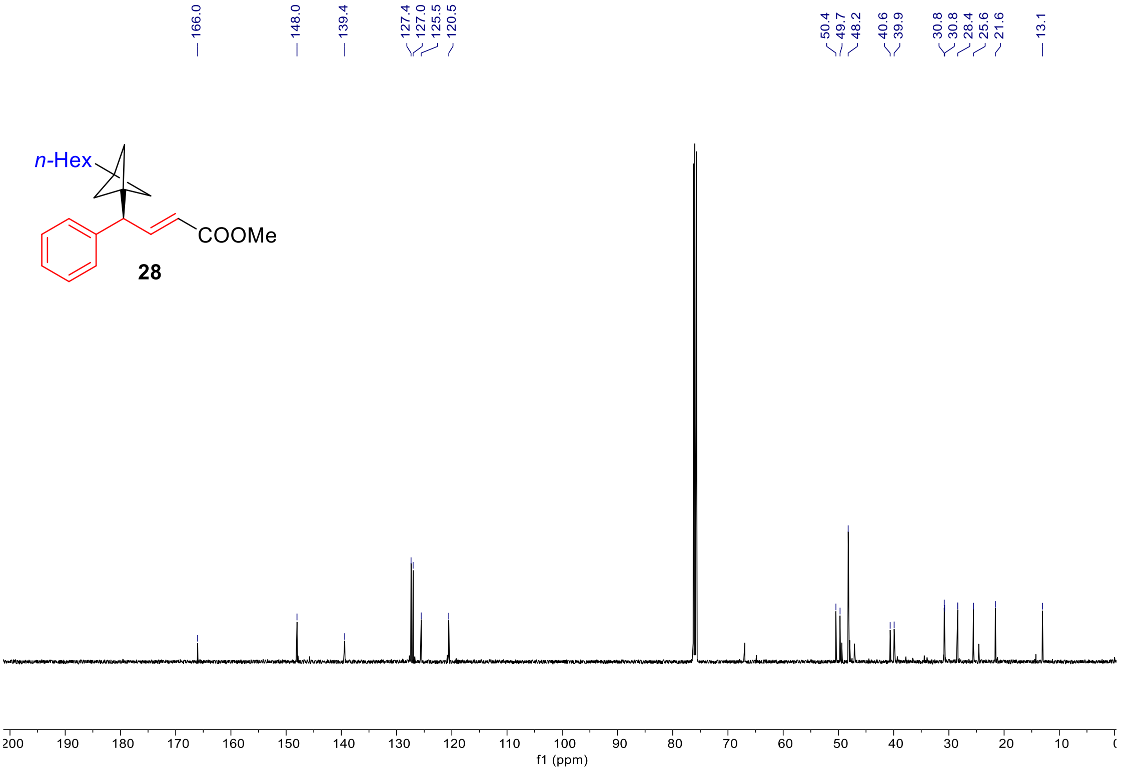

1H-NMR (CDCl3, 500 MHz)

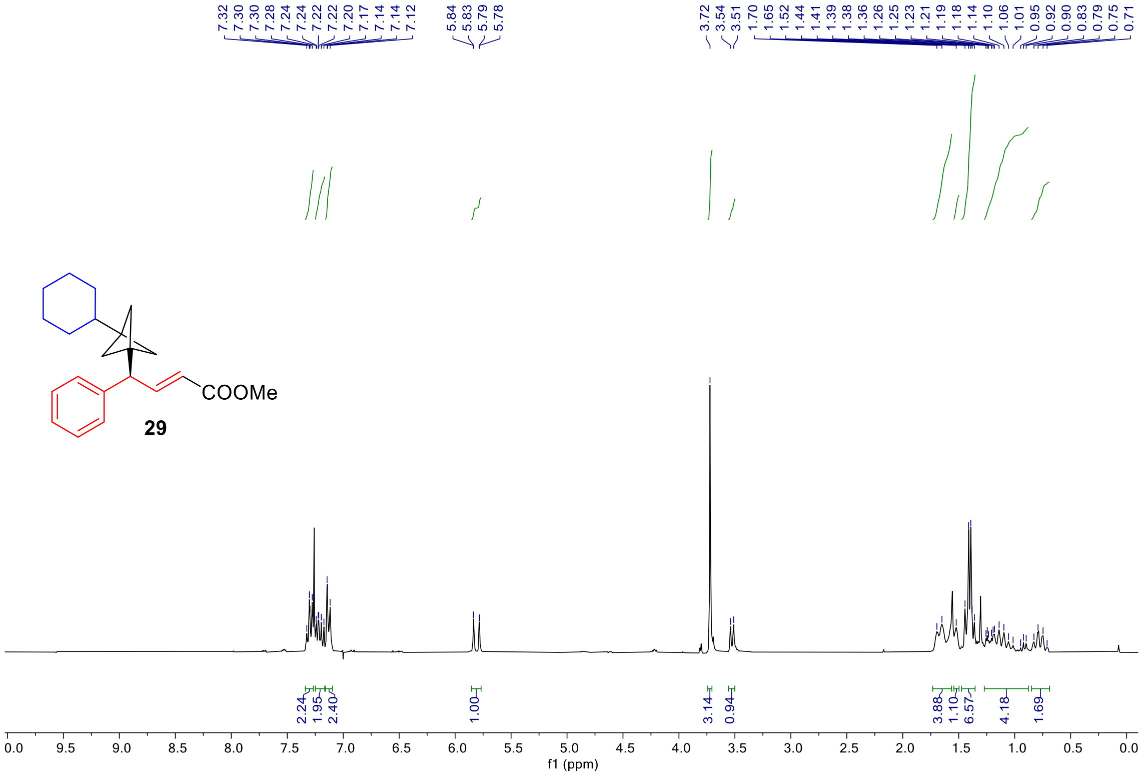

13C-NMR (CDCl3, 126 MHz)

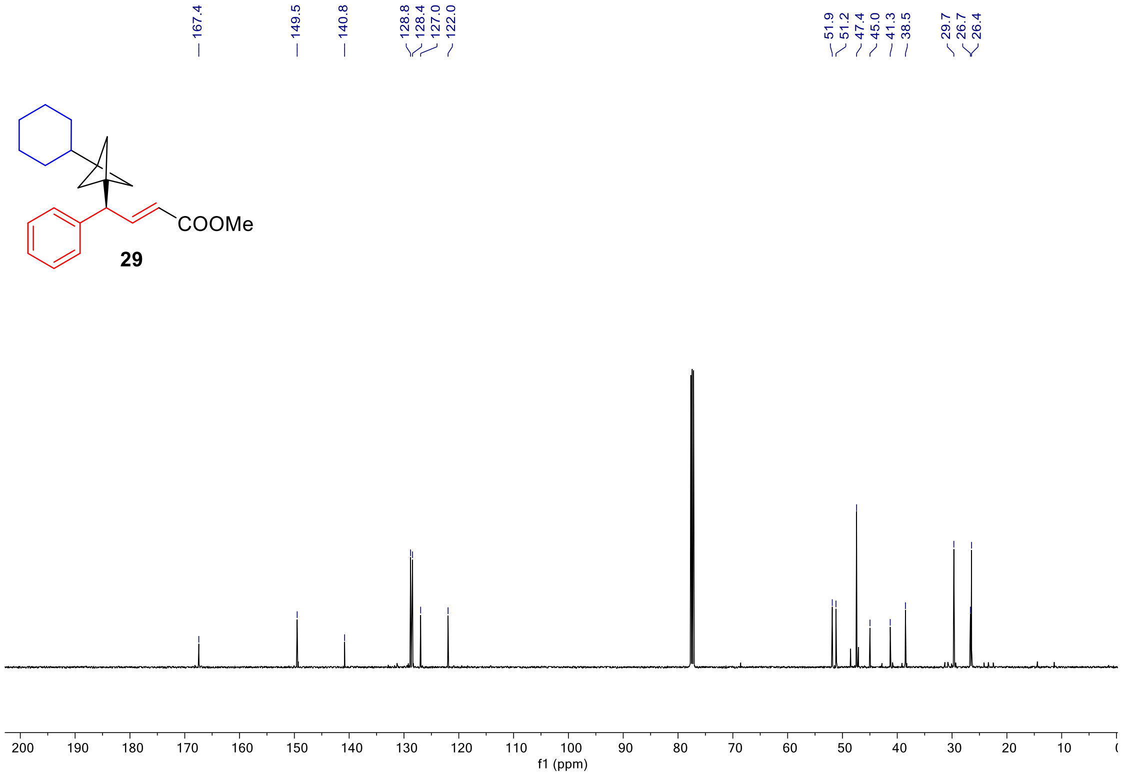

1H NMR (CDCl3, 500 MHz)

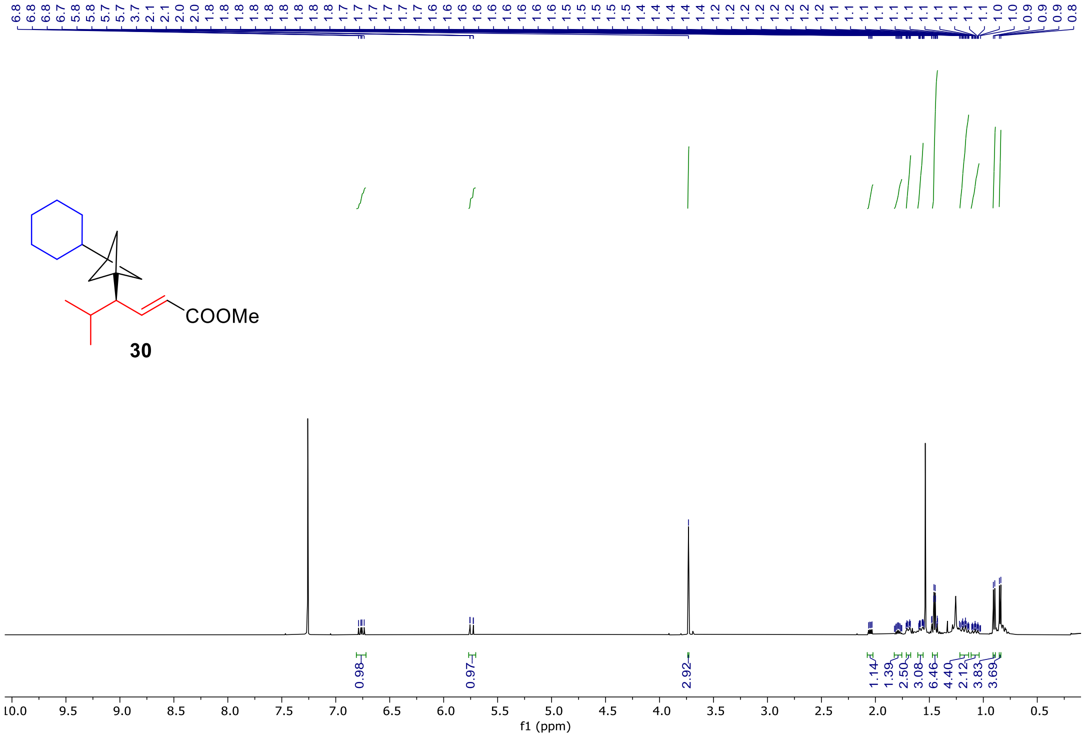

13C NMR (CDCl3, 126 MHz)

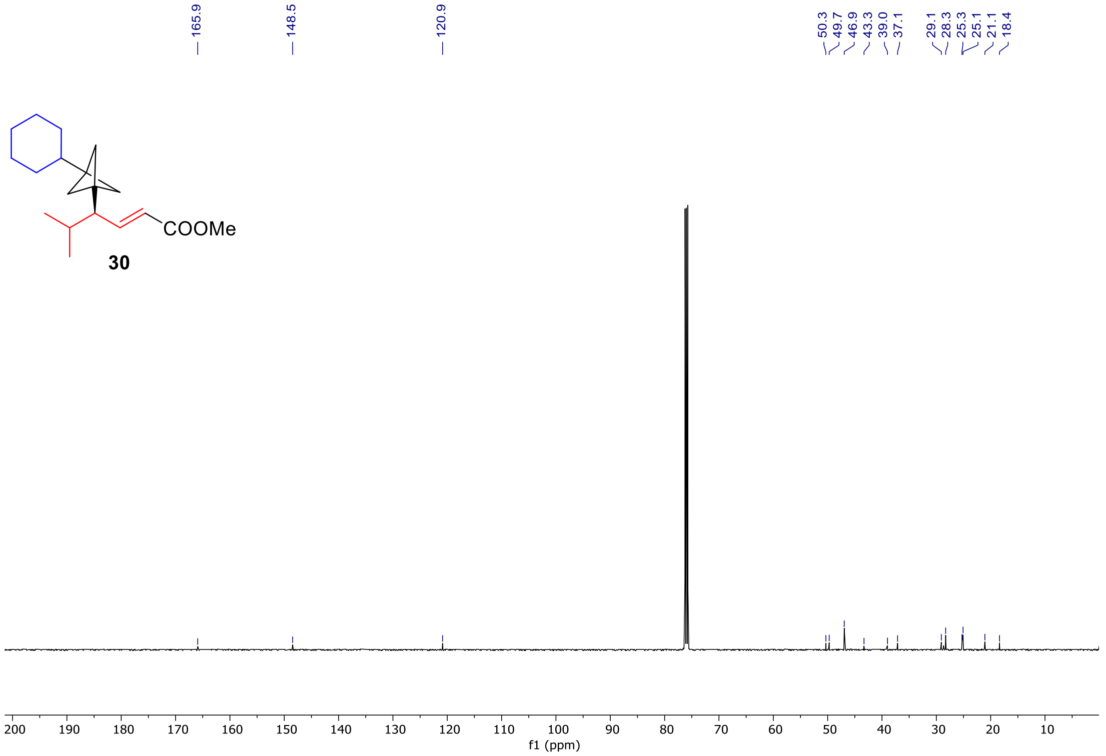

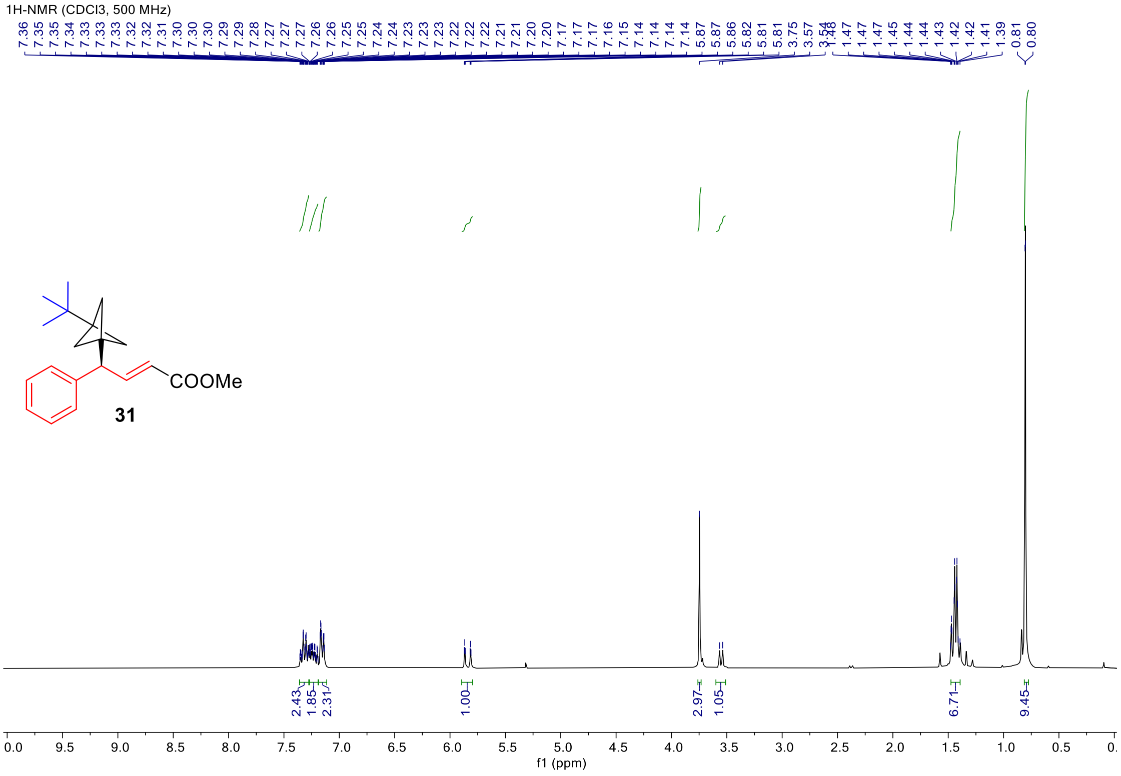
1H-NMR (CDCl3, 500 MHz)
7.36 7.35 7.35 7.34 7.33 7.33 7.32 7.32 7.31 7.30 7.30 7.29 7.29 7.28 7.27 7.27 7.27 7.26 7.26 7.25 7.25 7.24 7.24 7.23 7.23 7.23 7.22 7.22 7.22 7.21 7.21 7.20 7.20 7.17 7.17 7.16 7.15 7.14 7.14 7.14 7.14 5.87 5.87 5.86 5.82 5.81 5.81 3.75 3.57 3.54 1.48 1.47 1.47 1.45 1.44 1.44 1.43 1.42 1.42 1.41 1.39 0.81 0.80
COOMe
31
2.43 1.85 2.31 1.00 2.97 1.05 6.71 9.45
f1 (ppm)

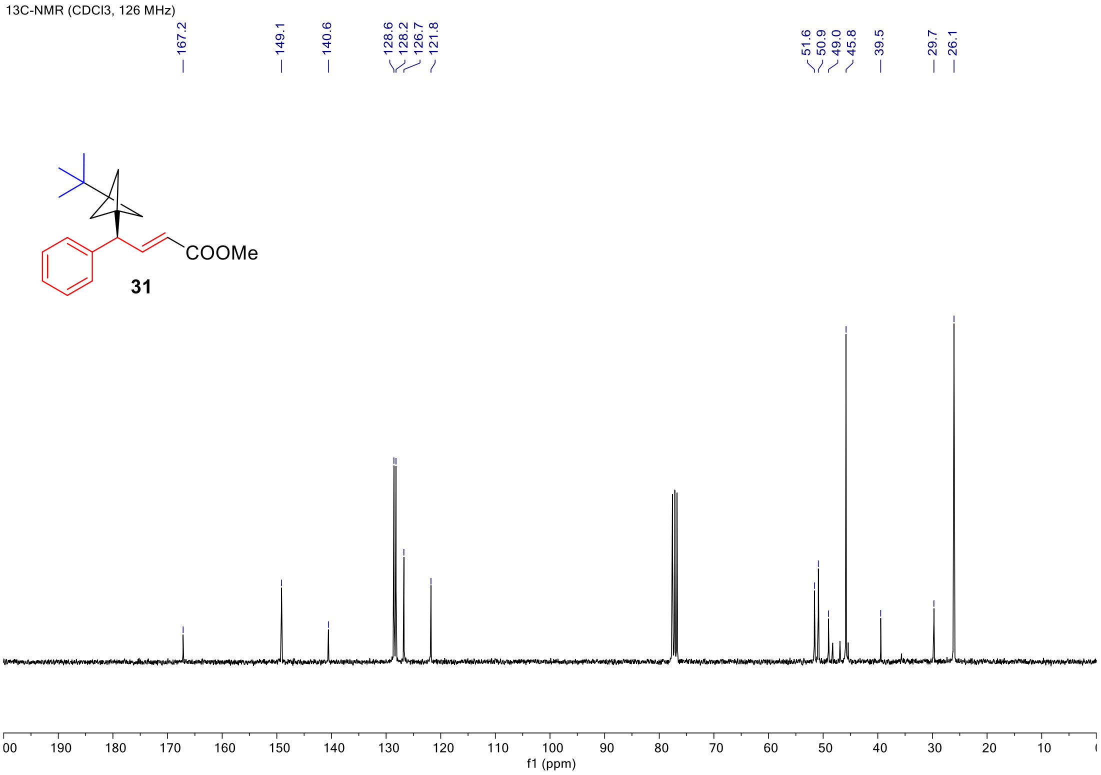
13C-NMR (CDCl3, 126 MHz)
167.2 149.1 140.6 128.6 128.2 126.7 121.8 51.6 50.9 49.0 45.8 39.5 29.7 26.1
COOMe
31
f1 (ppm)

1H NMR (CDCl3, 500 MHz)

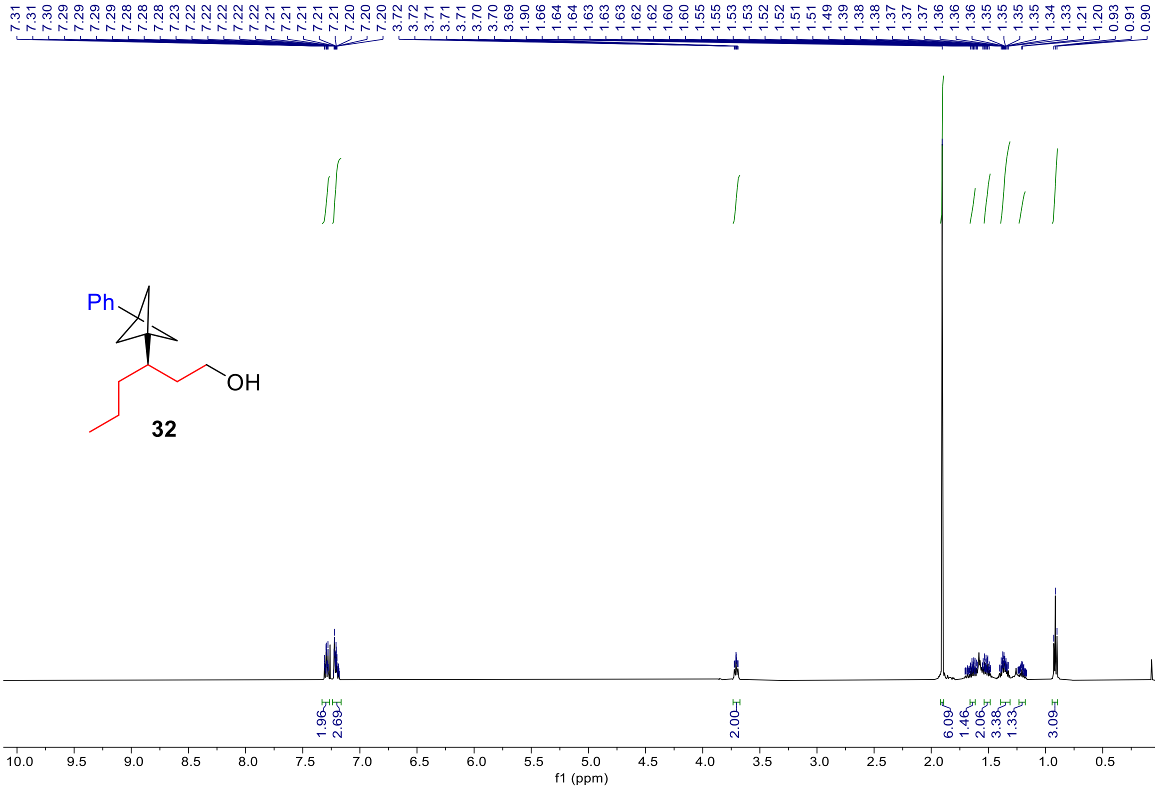

13C NMR (CDCl3, 126 MHz)

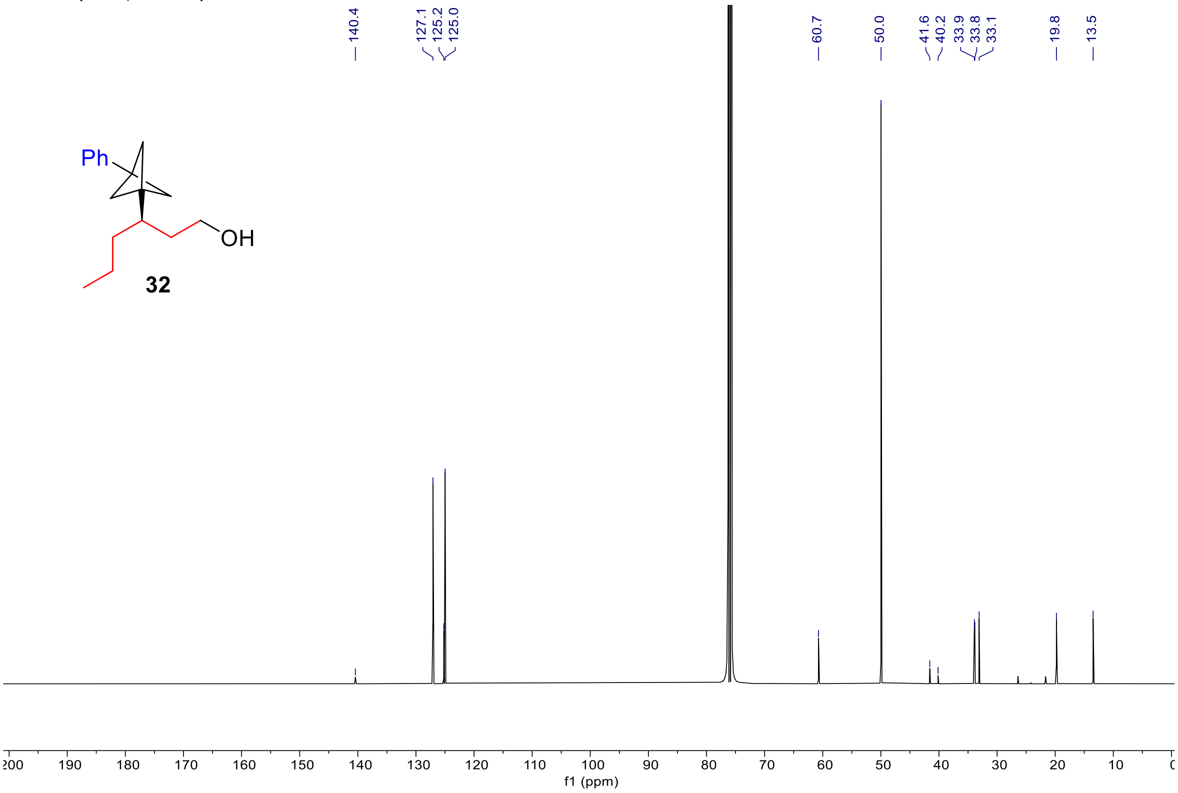

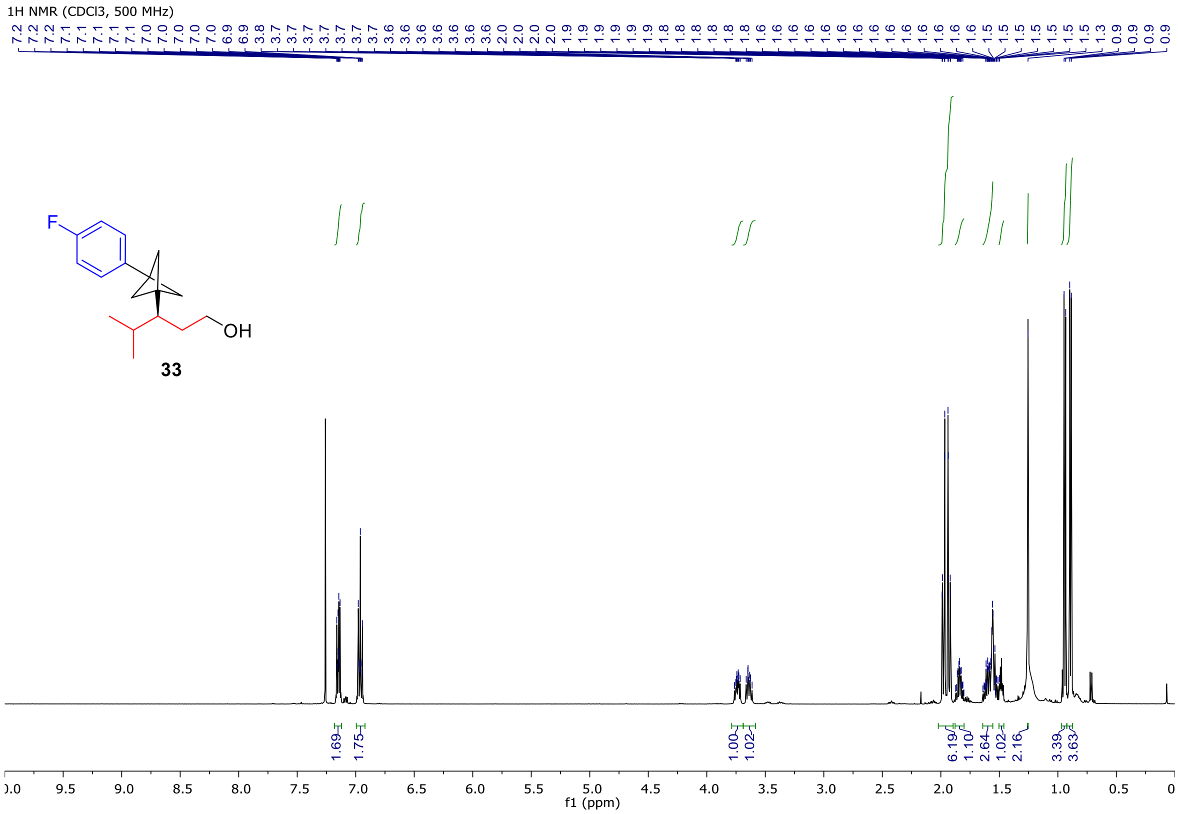
1H NMR (CDCl3, 500 MHz)
F
OH
33
f1 (ppm)

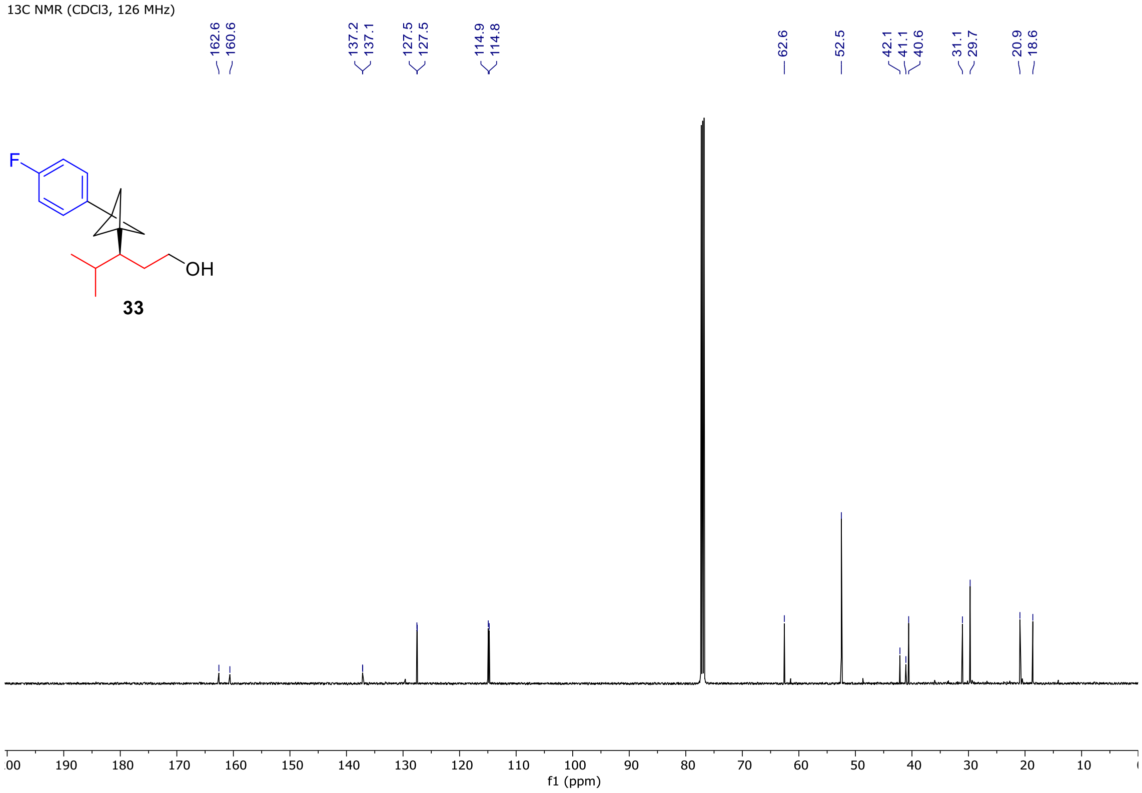
13C NMR (CDCl3, 126 MHz)
162.6
160.6
137.2
137.1
127.5
127.5
114.9
114.8
62.6
52.5
42.1
41.1
40.6
31.1
29.7
20.9
18.6
F
OH
33
f1 (ppm)

19F NMR (CDCl3, 282 MHz)

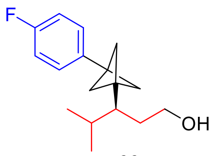

33

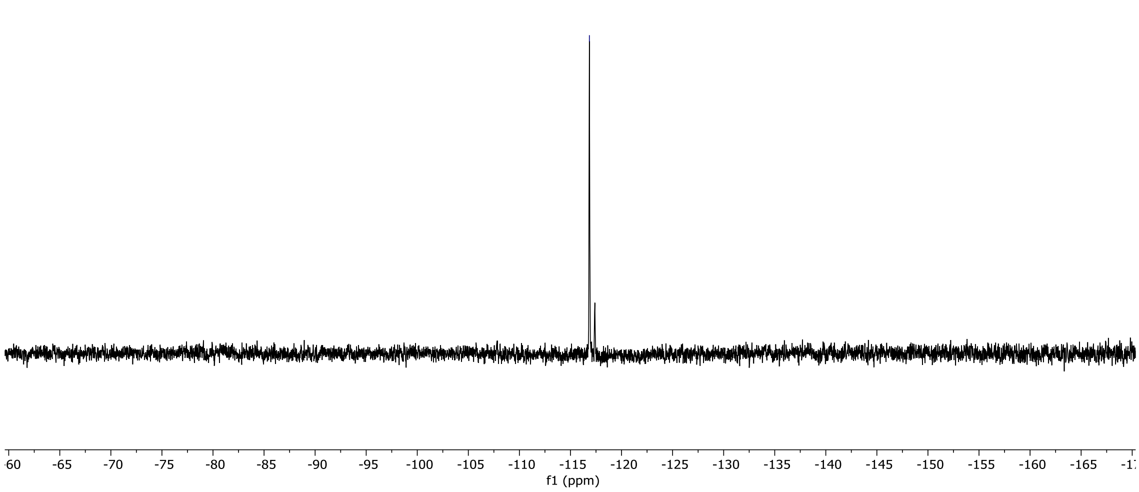

14. References

1. Yu, S.; Jing, C.; Noble, A. Aggarwal, V. K., *Org. Lett*. **2020**, *22*, 5650.

2. Gianatassio, R.; Lopchuk, J. M.; Wang, J.; Pan, C.-M.; Malins, L. R.; Prieto, L.; Brandt, T. A.; Collins, M. R.; Gallego, G. M.; Sach, N. W.; Spangler, J. E.; Zhu, H.; Zhu, J.; Baran, P. S. *Science* **2016**, *351*, 241.

3. Makarov, I.S.; Brocklehurst, C. E.; Karaghiosoff, K.; Koch, G.; Knochel, P. *Angew. Chem. Int. Ed.* **2017**, *56*, 12774.

4. Delvos, L. B.; Vyas, D. J., Oestreich, M. *Angew. Chem. Int. Ed.* **2013**, *52*, 4650.

5. Lee, J.; Torker, S.; Hoveyda, A. H. *Angew. Chem. Int. Ed.* **2017**, *56*, 821.

6. Yurino, T.; Tani, R.; Ohkuma, T. *ACS Catal*. **2019**, *9*, 4434.

7. Brown, M. K.; May, T. L.; Baxter, C. A.; Hoveyda, A. H. *Angew. Chem. Int. Ed.* **2007**, *46*, 1097.

8. Sánchez-Sordo, I.; Chaves-Pouso, A.; Mateos-Gil, J.; Rivera-Chao, E.; Fañanás-Mastral, M., *Chem Catalysis* **2023**, *3*, 100730.

9. Gong, T. J.; Yu, S. H.; Li, K.; Lu, X.; Xiao, B.; Fu, Y. *Chem. Asian J.* **2017**, *12*, 2884.

10. Prasad, B. A. B.; Gilbertson, S. R.; *Org. Lett.* **2009**, *11*, 3710.
